# Genetic architecture of a Circadian Imbalance Index: genome-wide association, phenome-wide association, and Mendelian randomisation analyses

**DOI:** 10.1016/j.ebiom.2026.106380

**Published:** 2026-07-21

**Authors:** Magdalena Żebrowska, Matthias Wielscher, Jing Zhang, Ingvild Saksvik-Lehouillier, Lee DiMilia, Angus Burns, Jesse Valliere, Leonardo Vincenzi, Susan Redline, Olivia Okereke, Richa Saxena, Rebecca Richmond, Martin K. Rutter, Eva S. Schernhammer

**Affiliations:** a Department of Epidemiology, Center for Public Health, Medical University of Vienna, Vienna, Austria; b Channing Division of Network Medicine, Department of Medicine, Brigham and Women's Hospital and Harvard Medical School, Boston, MA, USA; c Department of Epidemiology, Harvard T.H. Chan School of Public Health, Boston, MA, USA; d Division of Sleep Medicine, Harvard Medical School, Boston, MA 02115, USA; e Division of Sleep and Circadian Disorders, Brigham and Women's Hospital, Boston, MA 02115, USA; f Center for Genomic Medicine, Massachusetts General Hospital, Boston, MA 02114, USA; g Department of Anesthesia, Critical Care and Pain Medicine, Massachusetts General Hospital and Harvard Medical School, Boston, MA 02114; h Department of Dermatology, Medical University of Vienna, Vienna, Austria; i Department of Psychology, Norwegian University of Science and Technology, NO-7491 Trondheim, Norway; j NIHR Oxford Health Biomedical Research Centre, University of Oxford, Oxford, UK; k School of Business and Law, Central Queensland University, Rockhampton, Queensland, Australia; l Turner Institute for Brain and Mental Health, School of Psychological Sciences, Faculty of Medicine, Nursing and Health Sciences, Monash University, Melbourne, VIC, Australia; m Program in Medical and Population Genetics, Broad Institute of MIT and Harvard, Cambridge, MA, USA; n Centre for Biological Timing, Division of Endocrinology, Diabetes and Gastroenterology, School of Medical Sciences, Faculty of Biology, Medicine and Health, Manchester Academic Health Science Centre, University of Manchester, Manchester, UK; o Diabetes, Endocrinology and Metabolism Centre, NIHR Manchester Biomedical Research Centre, Manchester University NHS Foundation Trust, Manchester, UK; p MRC Integrative Epidemiology Unit at the University of Bristol, Bristol, UK; q Population Health Sciences, Bristol Medical School, University of Bristol, Bristol, UK; r Department of Psychiatry, Massachusetts General Hospital and Harvard Medical School, Boston, MA, 02215, USA

**Keywords:** Circadian imbalance index, Circadian disruption, GWAS, Shift work

## Abstract

**Background:**

Circadian disruption affects multiple aspects of human health, but the genetic architecture of individual susceptibility remains unclear. We examined the genetics of the Circadian Imbalance Index (CII), an additive 0–5 score combining evening chronotype, short/long sleep, high neuroticism, atypical caffeinated coffee intake, and low vitamin D.

**Methods:**

We ran a genome-wide association study (GWAS) of CII in 312,935 European-ancestry UK Biobank participants, compared signals with component-specific and leave-one-component-out (LOCO) GWASs, and tested sex and shift work interactions. CII polygenic score (PRS) PheWAS analyses were evaluated in Mass General Brigham Biobank (N = 50,908) and All of US (N = 98,182), with component-weighted PRS sensitivity analyses. Replication was assessed in Nurses’ Health Study II women (N = 11,344). We estimated genetic correlations and performed bidirectional two-sample Mendelian randomisation (MR) with MR-Egger, weighted median and LOCO sensitivity analyses.

**Findings:**

We identified 27 loci mapping to 72 genes, including genes with reported links to circadian regulation or circadian-related pathways, such as CALCA, DHCR7, KDM5A, HAL, and CRX. Five genes (EPHB1, SERPING1, C12orf74, PLEKHG7, and EEA1) were observed in the CII analysis but not in component-specific analyses. LOCO analyses indicated that the CII genetic signal was not reducible to any single component. The CII PRS was associated with metabolic and psychiatric phenotypes. CII was genetically correlated with insomnia, mood swings, body mass index, type 2 diabetes, coronary artery disease, and myocardial infarction. MR analyses provided suggestive directional evidence, strongest for reverse associations of mood swings and coronary artery disease with CII.

**Interpretation:**

The CII captures a polygenic composite susceptibility signal associated with cardiometabolic and mood outcomes, with suggestive evidence of directional relationships.

**Funding:**

European Research Council Advanced Grant CLOCKrisk (101053225).

**Research in context**

**Evidence before this study**

We performed a targeted literature search to identify genetic studies of composite or integrative measures of circadian susceptibility. We searched PubMed, Web of Science, Google Scholar, and the GWAS Catalogue from database inception to June 5, 2026, with no language restrictions, using terms related to “circadian imbalance index”, “circadian disruption”, “circadian misalignment”, “circadian susceptibility”, “composite score”, “composite index”, “integrative measure”, “GWAS”, “genome-wide association study”, “polygenic score”, “polygenic risk score”, “PheWAS”, and “Mendelian randomisation”. The search was designed to identify studies of composite circadian susceptibility measures rather than studies of individual CII components. After manual screening, we did not identify a genetic study evaluating a composite CII-like measure integrating sleep, behavioural, psychological, caffeine-related, and vitamin D-related domains. In contrast, existing genetic literature has examined individual contributors to circadian disruption, including chronotype, sleep duration, neuroticism, caffeine intake, and vitamin D, generally treating these traits as separate phenotypes.

**Added value of this study**

We conducted a genome-wide association study of a Circadian Imbalance Index (CII) in more than 300,000 European ancestry UK Biobank participants and identified multiple genetic regions and mapped genes, including circadian-related genes. Most signals overlapped with individual CII components, while a small number of genes were detected in the CII analysis but not in component-specific analyses, consistent with potential added information from the composite phenotype. Leave-one-component-out analyses indicated that the CII genetic signal was not reducible to a single component, although vitamin D-related loci contributed strongly to several leading associations. We also evaluated differences by biological sex and shift work, with stronger support for sex-specific genetic effects and exploratory evidence for shift-work modification. Using a polygenic score in two independent biobanks, we observed broad links between CII genetic liability and metabolic, psychiatric, and cardiovascular phenotypes. Component-weighted PRS PheWAS analyses showed narrower component-related associations. Finally, genetic correlation and MR analyses supported connections between the CII and insomnia, cardiometabolic disease, and mood swings, with MR findings interpreted as suggestive given multiple testing, composite phenotype structure, and possible pleiotropy.

**Implications of all the available evidence**

Individual susceptibility to circadian disruption is not driven by a single pathway but reflects the combined effects of multiple behavioural, psychological, and biological factors with a shared genetic basis. The CII may provide a useful framework for studying this multidimensional susceptibility alongside, rather than instead of, component-specific measures. Further validation in diverse populations, together with refinement using objective sleep, rest-activity, and light exposure measures, will be needed before the CII or its PRS can be used for risk stratification. Such integrative approaches may help identify individuals whose vulnerability to metabolic, cardiovascular, or mental health outcomes related to circadian disruption is not fully captured by single traits such as sleep duration or chronotype alone.

## Introduction

The human circadian system is an internal timekeeping network, with the suprachiasmatic nucleus (SCN) acting as the master pacemaker^1^ that synchronises physiological and behavioural functions to the 24-h light–dark cycle via rhythmic expression of clock genes. Together with the homoeostatic sleep process, it forms the two-process model of sleep,^2^^,^^3^ which regulates sleep timing and duration. When these circadian and homoeostatic processes are synchronised, sleep-wake cycles remain aligned, whereas if they are desynchronised—circadian misalignment occurs.^2^^,^^3^

Circadian misalignment has been linked to diverse adverse health outcomes, including impairments in sleep, metabolism, mental health, cognition, cardiovascular functions and immune response.^3^, ^4^, ^5^, ^6^ These disruptions arise from interacting environmental, behavioural, and genetic influences,^3^ contributing to substantial inter-individual variability in susceptibility. To create a comprehensive measure of susceptibility to circadian disruption, we recently developed the Circadian Imbalance Index (CII),^7^ an additive score combining five partly heterogeneous but related domains: evening chronotype, short or long sleep duration, low serum vitamin D, no or high caffeine consumption, and high neuroticism. The CII is therefore intended as an empirically motivated cumulative susceptibility index rather than as a direct measure of circadian phase, circadian timing, or a single unified biological mechanism. In UK Biobank, higher CII was associated in a dose–response manner with cardiovascular-kidney-metabolic (CKM) disease risk, particularly among European-ancestry participants engaged in shift or night-shift work, consistent with the possibility that the composite score captures vulnerability relevant to external circadian stressors.

The five CII domains have partially overlapping genetic and biological links with sleep, psychiatric, metabolic, behavioural, and light-exposure-related traits. Chronotype-associated variants have been linked to psychiatric traits, including schizophrenia, depressive symptoms, and major depressive disorder, and broader genetic analyses have shown substantial shared architecture between sleep-related phenotypes and psychiatric disorders.^8^ Chronotype-associated loci include circadian-related genes (e.g., PER2, RGS16) and have also been linked with cardiometabolic traits, including obesity and type 2 diabetes.^8^ Variants influencing short sleep duration show causal links to chronic pain and adverse cardiometabolic outcomes.^9^ Neuroticism is also genetically correlated with insomnia and related sleep traits, supporting overlap between psychological vulnerability and sleep-related biology.^10^ Vitamin D may modulate circadian regulation through pathways involving clock gene synchronisation (e.g., BMAL1, PER2, NPAS2),^11^ and related transcriptional mechanisms. Caffeine can delay the human circadian clock^12^ and alter expression and synchronisation of core clock genes (Clock, Per2, Cry1, Cry2).

Together, these observations motivate evaluating the genetic architecture of the CII as a composite phenotype, while also comparing its signals with those of the individual components. Here, we conduct a large-scale GWAS of CII in a European-ancestry UK Biobank discovery cohort (N = 312,935), with replication in Nurses’ Health Study II (NHS2; N = 11,344).^13^ We assess effect modification by sex and shift-work status, perform PheWAS of the CII polygenic risk score in the Mass General Brigham Biobank (MGBB; N = 50,908)^14^ and All of US (AoU; N = 98,182),^15^ and follow PheWAS signals with genetic correlation and MR to assess potential directional relationships. We focus on the genetic architecture of the CII and its broader phenome-wide and genetic associations, aiming to characterise the genetic architecture of the CII and its broader phenome-wide and genetic associations with disease outcomes related to circadian disruption. Throughout this paper, we use “circadian disruption” to describe the broader biological and behavioural phenomenon, “circadian imbalance” to refer to the multidimensional construct operationalised by the CII, and “circadian susceptibility” to denote an individual predisposition to circadian disruption.

## Methods

### UK Biobank

The genetic and phenotypic data for this study were obtained from the UK Biobank^16^ (UKB) under application 48,576. The UK Biobank is a large prospective cohort study that began in 2006 and enrolled approximately 500,000 UK residents aged 40–69 years. At baseline, participants provided comprehensive data on sociodemographic, psychosocial, physical, and lifestyle factors, along with biological samples. Health outcomes are tracked longitudinally through linkages to national datasets, including Hospital Episode Statistics and national death and cancer registries.

#### Ethics

The UKB study was approved by the UK National Research Ethics Service (ref. 11/NW/0382), and all participants provided written informed consent. The NHS2 protocol was approved by the Institutional Review Board of Brigham and Women's Hospital and the Committee on the Use of Human Subjects in Research of Harvard T.H. Chan School of Public Health (Boston, MA, USA). Voluntary return of questionnaires indicates informed consent. The MGBB protocol is approved by the Mass General Brigham Institutional Review Board (IRB protocol no. 2009P002312), and participants provided informed consent for use of their samples and health information in biological and genetic research. The AoU Research Program protocol was reviewed by the All of Us Institutional Review Board. All participants provided informed consent, either in person or through an electronic consent platform, including primary consent for research participation, HIPAA authorisation for research use of electronic health records and other external health data, and consent for return of genomic results. The present manuscript reports secondary analyses of previously collected de-identified data. No new participant recruitment, contact, intervention, or biological sample collection was undertaken, and no additional study-specific institutional approval was sought for these analyses, which were conducted under the existing cohort-specific ethics approvals and data-access governance frameworks.

### Circadian Imbalance Index definition and study population

In our recent study^7^ we developed a novel Circadian Imbalance Index (CII) in the UKB summing up five indicator components increasing individual's propensity of circadian disruption (1) evening chronotype, defined as self-reported identification as “definitely an evening person” or “more an evening than a morning person”; (2) short (<7 h/day) or long (≥9 h/day) sleep duration; (3) high neuroticism, defined as scoring ≥7 on the neuroticism scale^17^^,^^18^; (4) atypical caffeinated coffee consumption, defined as either no intake or high intake (≥5 cups/day); and (5) low serum vitamin D concentration (<50 nmol/L). Participants not meeting the criteria for a given trait received a score of zero for that component. The CII was calculated as the sum of points across these five domains, yielding a total score ranging from 0 to 5, with higher scores reflecting greater likelihood of circadian imbalance. Further methodological details are provided in Zhang et al.^7^

Each component captures a distinct but related dimension of circadian vulnerability. Chronotype reflects differences in circadian alignment and is associated with habitual sleep–wake timing and, in part, meal timing.^19^, ^20^, ^21^ Sleep duration is influenced by circadian phase, with both short and long sleep linked to greater circadian disruption.^22^ In the UKB sleep duration was assessed based on the question asking about total sleep in every 24 h, including naps. Accordingly, the long-sleep component (≥9 h/day) should be interpreted as a marker of extended total daily sleep, which may in some participants reflect daytime napping or multiple sleep bouts and thus potentially capture circadian instability rather than nocturnal sleep need alone. Although neuroticism is not a circadian-specific trait and may partly reflect broader psychological distress, it was included in the CII as one dimension of susceptibility to circadian disruption because of its reported links to shift-work tolerance and sleep-wake variability.^6^^,^^23^ The caffeine component was restricted to caffeinated coffee intake, as information on tea consumption and timing of coffee intake was not available in the data version used for this study. Caffeine directly delays the human circadian clock.^12^ In adolescents, its effects on slow-wave sleep (SWS) and melatonin onset vary widely with those showing greater SWS reduction or delayed melatonin being especially vulnerable to sleep–wake desynchronisation.^24^ As an adenosine receptor antagonist, caffeine's impact depends on adenosine A2A receptor function and individuals with altered adenosinergic signalling show greater impairment of alertness and readiness during sleep deprivation, linking caffeine sensitivity to circadian resilience. Genetically, caffein sensitivity depends partly on ADORA2A polymorphisms–people with certain variants (TT genotype of rs5751876) experience more anxiety, insomnia, or overstimulation at standard doses and are more likely to avoid coffee.^25^^,^^26^ Additionally, CYP1A2 variants slow down caffeine metabolism, keeping its levels elevated for longer and increasing symptoms such as palpitations or anxiety, which often leads to self-regulated coffee avoidance.^27^ Genetic factors overall explain around 36–58% of individual differences in caffeine consumption, predisposing some to drink less or no coffee.^28^ Based on these research findings, we used “0 cups of caffeinated coffee” as a behavioural proxy for potential caffeine intolerance or genetic hypersensitivity. Low serum vitamin D may reflect multiple determinants, including sunlight exposure,^29^ dietary intake, supplementation, assessment season and individual metabolic variation. Low levels have been associated with poorer sleep and sleep disorders, consistent with roles in sleep–wake regulation, melatonin-related pathways, and circadian gene expression.^11^^,^^30^^,^^31^

From the 409,460 UKB participants classified as Europeans using genetically inferred ancestry grouping, as defined by the Pan-UK Biobank,^32^ we excluded individuals with missing values (including “prefer not to answer”) at any of the factors used for construction of the CII, leading to a study sample of 312,935 subjects.

### Statistics

#### Genome wide association studies in the UKB

We performed six genome wide association studies (GWASs)–of the five CII components defined as binary phenotypes and the CII itself–with regenie v3.4.1.^33^ For quality control, we excluded SNPs with a genotyping efficiency below 80%, minor allele frequencies (MAF) below 1%, or Hardy–Weinberg equilibrium (HWE) values below 1 × 10^−8^. Further quality control details can be found in Supplementary Methods. We applied linear (for the CII), or logistic (for the CII components) regression models adjusted for age, sex, batch, assessment centre and the first 20 genetic principal components. The GWAS model for low vitamin D levels was additionally adjusted for assessment season. We applied a conventional genome wide significance threshold of p < 5 × 10^−8^. For functional annotation we used Functional Mapping and Annotation (FUMA^34^) v1.5.2 (Supplementary Methods). To assess whether the constructed CII captures a meaningful genetic signal, we estimated the SNP-based heritability using Linkage Disequilibrium Score Regression (LDSC).^35^

#### Genetic correlation analysis

To evaluate the extent to which the CII captures information that is shared with or distinct from its underlying components, we estimated both phenotypic and genetic correlations between the CII and each of its five component traits. Genetic correlations were calculated using LDSC.^35^

#### Genome-wide SNP × shift work interaction analysis

Shift work status was defined among genotyped European ancestry UKB participants with available CII components (N = 312,935) who replied to the baseline questionnaire (2006–2010) and answered the question if their primary job involved shift work (N = 183,238), defined as work schedules outside typical daytime hours (9am–5pm). Those who answered with ‘sometimes’, ‘usually’ or ‘always’ (N = 28,976) were categorised as ‘shift workers’, whereas those who answered ‘never/rarely’ (N = 154,262) were categorised as ‘no shift workers'. Participants who answered with ‘prefer not to answer’ and ‘do not know’ were excluded from this analysis.

To evaluate whether the genetic effects on the CII differ according to the shift work status, we further conducted a genome-wide gene–environment (GxE) interaction analysis. Specifically, we extended the GWAS model to include an interaction term between each SNP and shift work status (SNP × shift work), while adjusting for the same set of covariates as in the primary analysis. For loci reaching genome-wide significance (p < 5 × 10^−8^), we further characterised associations by performing stratified GWAS in shift workers and non-shift workers separately, thereby estimating group-specific effect sizes and directions. In addition, we quantified cross-stratum heterogeneity using a Wald test of the difference in effects and examined the SNP × SW interaction p-values from the joint model.

#### Replication in the Nurses’ Health Study II

For the aims of replication, we constructed the CII in the women-only, independent study cohort—he Nurses’ Health Study II (NHS2)^13^ – which contained relevant phenotype data. Information on all the five CII components was available for 50,900 European ancestry women. Of them 11,351 had genetic data available. Shift workers in the NHS2 were defined as ever performing shift work (before assessment year 2009). Among 11,351 genotyped women there were 11,344 with shift work assessments; of them N = 8124 (71.6%) nurses were shift workers and N = 3220 (28.4%) never performed shift work before 2009. Further details on the NHS2 cohort, CII construction, its quality check and description of genetic data in the NHS2 are available in Supplement 2.

Since our replication cohort contained only women, we first conducted a genome-wide SNP × sex interaction analysis in the UKB to test whether the genetic effects on the CII differed between men and women. Sex was defined using the UK Biobank genetic sex variable (Data Field 22,001). The interaction model included the main effects of the SNP and sex, as well as their product term (SNP × sex) to capture sex-specific modifications of genetic associations. Association testing was carried out genome-wide, and statistical significance of sex-interaction effects was determined based on the p-value of the interaction coefficient. Genome-wide significance was defined at the conventional threshold of p < 5 × 10^−8^. For loci reaching genome-wide significance, we further characterised the associations by conducting follow-up stratified GWASs in men (N = 148,473) and women (N = 164,462) separately. We further quantified cross-stratum heterogeneity using a Wald test of the difference in effects and examined the SNP × sex interaction p-values from the joint model.^36^

Replication of CII GWAS in the NHS2 was performed with PLINK 1.9 by assessing effect estimates and p-values from an association test with the CII for variants which attained significance in the UKB-women GWAS. Linear regression models were adjusted for age and 10 first principal components of ancestry.

#### PheWAS in a clinical and population-based biobanks

Further we aimed to determine which external traits and disease phenotypes show association with the CII. We constructed polygenic risk score (PRS) for the CII, overall and stratified by genetic sex, using the PRS-CS^37^ and conducted a phenome-wide association studies (PheWAS) for CII-PRSs in a clinical biobank (MGBB^14^) and in a population-based cohort (AoU^15^). PheWAS analyses in both MGBB and AoU were conducted among European-ancestry participants. Associations were considered significant if their p-values were below the Bonferroni corrected significance levels (p_MGBB_ <0.05/1567 = 3.02E-5; p_AoU_ < 0.05/933 = 5.36E-5). Further details on the MGBB, AoU and the PheWAS are described in Supplement 2.

As sensitivity analyses, we generated component-weighted PRSs by reweighting the same CII-associated SNP set using effect estimates from each component-specific GWAS and evaluated their PheWAS associations in MGBB and AoU. This fixed-SNP approach was used to assess whether the phenome-wide associations carried by CII-associated variants were mainly explained by their effects on any individual CII component.

#### Specification of CII associated phenotypes

To identify phenotypes associated with CII, we considered (i) GWAS catalogue traits and phenotypes linked to gene sets identified in FUMA results and (ii) disease phenotypes identified as significantly associated with the CII-PRS in our PheWAS analyses. For these predefined traits we used UKB-based GWAS summary statistics and calculated their genetic correlation with the CII using LDSC. Those that showed a significant genetic correlation with CII were used in further causality verification.

#### Two sample Mendelian randomisation

To check causality and direction of associations between CII and the predefined list of phenotypes as described above, we performed bidirectional two-sample MR using the generalised summary-data–based MR (GSMR) framework implemented in GCTA^38^, ^39^, ^40^(v1.94.1). Summary statistics for the CII were obtained from our UKB GWAS, whereas summary statistics for the predefined phenotypes were obtained from non–UKB sources (Supplementary Table S23). The MR analysis was run in both directions (Exposure-to-Outcome and Outcome-to-Exposure). We implemented GSMR with HEIDI-outlier filtering (P < 0.01) to mitigate horizontal pleiotropy. Instruments were genome-wide significant variants (P < 5 × 10^−8^) clumped for independence (LD r^2^ < 0.05) using an LD reference of 425,406 individuals; SNPs with allele mismatches/missingness and large allele-frequency discrepancies versus the LD reference were excluded prior to GSMR. Analyses were required to retain at least 10 instruments after filtering. To further assess robustness to horizontal pleiotropy, we performed MR-Egger and weighted median sensitivity analyses for the key GSMR associations, comparing the direction and magnitude of effect estimates across methods and evaluating the MR-Egger intercept as a test of directional pleiotropy.

#### Leave-one-component-out GWAS and MR sensitivity analyses

To evaluate whether the genetic signal of the CII was strongly influenced by any single component, we performed leave-one-component-out analyses. Specifically, we constructed five alternative indices by excluding, one at a time, evening chronotype, short or long sleep duration, high neuroticism, atypical, caffeinated coffee consumption, or low vitamin D, and summing the remaining four binary components. Each leave-one-component-out index was then analysed in a separate GWAS using the same quality-control procedures, regression framework, and covariate adjustment as in the primary CII GWAS. For variants reaching genome-wide significance in the primary CII GWAS, corresponding effect estimates, and p values were extracted from each leave-one-component-out GWAS for comparative assessment. Because the leave-one-component-out indices ranged from 0 to 4 rather than 0 to 5, comparisons focused on consistency of effect direction, relative attenuation, and statistical significance rather than exact equality of effect sizes.

To determine whether the associations observed in our GSMR analyses were attributable to the CII itself or dominated by a single component trait, we additionally performed leave-one-component-out MR analyses by using each reduced CII GWAS as the exposure in GSMR for the key forward associations identified in the primary MR analysis, applying the same genome-wide significance threshold, LD clumping, HEIDI-outlier filtering, minimum instrument requirement, and GSMR settings as for the full CII.

### Role of funders

The funders had no role in study design, data collection, data analysis, data interpretation, writing of the manuscript, or the decision to submit the manuscript for publication.

## Results

Table 1 presents baseline characteristics of the study participants, stratified by levels of the CII and summaries for each of the CII's components. A total of 312,935 European ancestry individuals with complete genotype, CII, and covariates data were included. Participants had a mean age of 56.6 years (SD = 8.1), a mean body mass index (BMI) of 27.4 kg/m^2^ (SD = 4.7) and were 47.4% male. Those with higher CII were less likely to be men (40.5% within CII = 5 vs 53.5% in CII = 0), have higher BMI (29.2 (SD = 5.9) in CII = 5 vs 26.4 (SD = 3.9) in CII = 0) and more likely to be involved in shift work (26.3% in CII = 5 vs 13% in CII = 0). Individuals with higher CII were also more likely to have an average total household income of less than £18,000 (33.3% in CII = 5 vs 14.7 in CII = 0) and less likely had college or university degree (23.9% in CII = 5 vs 35.7% in CII = 0).

**Table 1:** Baseline characteristics of 312,935 genotyped, European ancestry UK Biobank participants with available CII assessments, by CII value and CII components.

	CII value						Total
	0	1	2	3	4	5	
N (%)	29,538 (9.4)	87,028 (27.8)	103,761 (33.2)	65,257 (20.9)	23,254 (7.4)	4097 (1.3)	312,935
Age^a^	58.1 ± 7.8	57.2 ± 8.0	56.5 ± 8.0	55.8 ± 8.1	55.2 ± 8.1	54.2 ± 8.1	56.6 ± 8.1
Male, N (%)	15,810 (53.5)	43,253 (49.7)	48,700 (46.9)	29,271 (44.9)	9781 (42.1)	1658 (40.5)	148,473 (47.4)
BMI^a^	26.4 ± 3.9	26.9 ± 4.3	27.4 ± 4.7	27.9 ± 5.1	28.5 ± 5.5	29.2 ± 5.9	27.4 ± 4.7
Average total household income before tax, N (%)							
Less than £18,000	4325 (14.7)	13,504 (15.5)	18,301 (17.7)	13,593 (20.9)	5979 (25.8)	1354 (33.3)	5,7056 (18.3)
£18,000–£31,000	6602 (22.4)	19,224 (22.1)	22,747 (22.0)	14,478 (22.3)	5065 (21.9)	861 (21.2)	68,977 (22.1)
£31,000–£52,000	7213 (24.4)	21,001 (24.2)	24,690 (23.8)	15,105 (23.2)	5020 (21.7)	774 (19.0)	73,803 (23.6)
£52,000–£100,000	6066 (20.6)	17,882 (20.6)	20,307 (19.6)	11,522 (17.7)	3446 (14.9)	487 (12.0)	59,710 (19.1)
Greater than £100,000	1952 (6.6)	5221 (6.0)	5458 (5.3)	2631 (4.0)	750 (3.2)	87 (2.1)	16,099 (5.2)
Unknown	3352 (11.4)	10,070 (11.6)	12,040 (11.6)	7730 (11.9)	2879 (12.4)	504 (12.4)	36,575 (11.7)
Education, N (%)							
College or University degree	10,544 (35.7)	30,398 (34.9)	34,399 (33.2)	19,617 (30.1)	6266 (26.9)	980 (23.9)	102,204 (32.7)
Other	14,788 (50.1)	43,294 (49.7)	52,267 (50.4)	33,678 (51.6)	12,073 (51.9)	2168 (52.9)	158,268 (50.6)
Unknown	4206 (14.2)	13,336 (15.3)	17,095 (16.5)	11,962 (18.3)	4915 (21.1)	949 (23.2)	52,463 (16.8)
Shift workers, N (%^b^)	2020 (13.0)	6938 (13.8)	9628 (15.4)	7009 (17.8)	2821 (21.0)	560 (26.3)	28,976 (9.3)

	**CII components**					
	**Evening person**	**Short or long sleep**	**High neuroticism**	**No Or high caffeine**	**Low vit D**	**Total**
N (%)	105,673 (33.8)	96,903 (31.0)	74,742 (23.9)	161,085 (51.5)	165,419 (52.9)	312,935
Age^a^	55.7 ± 8.3	57.1 ± 7.9	55.3 ± 8.0	56.2 ± 8.1	56.0 ± 8.1	56.6 ± 8.1
Male, N (%)	49,943 (47.3)	46,105 (47.6)	28,978 (38.8)	72,382 (44.9)	78,472 (47.4)	148,473 (47.4)
BMI^a^	27.6 ± 4.8	28.0 ± 5.1	27.4 ± 5.1	27.5 ± 4.9	28.0 ± 5.1	27.4 (4.7)
Average total household income before tax, N (%)						
Less than £18,000	19,374 (18.4)	22,205 (23.0)	16,942 (22.7)	31,267 (19.5)	31,783 (19.3)	57,056 (18.3)
£18,000–£31,000	23,142 (22.0)	21,574 (22.3)	16,478 (22.1)	35,864 (22.3)	35,659 (21.6)	68,977 (22.1)
£31,000–£52,000	25,258 (24.0)	20,912 (21.7)	16,888 (22.7)	37,342 (23.2)	39,246 (23.8)	73,803 (23.6)
£52,000–£100,000	20,642 (19.6)	15,169 (15.7)	12,375 (16.6)	29,217 (18.2)	31,878 (19.3)	59,710 (19.1)
Greater than £100,000	5507 (5.2)	3945 (4.1)	2631 (3.5)	7147 (4.4)	8235 (5.0)	16,099 (5.2)
Unknown	11,505 (10.9)	12,746 (13.2)	9170 (12.3)	19,818 (12.3)	18,137 (11.0)	36,575 (11.7)
Education, N (%)						
College or University degree	35,511 (33.6)	26,268 (27.1)	21,327 (28.5)	47,558 (29.5)	57,347 (34.7)	102,204 (32.7)
Other	54,562 (51.6)	49,737 (51.3)	38,740 (51.8)	83,752 (52.0)	81,203 (49.1)	158,268 (50.6)
Unknown	15,600 (14.8)	20,898 (21.6)	14,675 (19.6)	29,775 (18.5)	26,869 (16.2)	52,463 (16.8)
Shift workers, N (%^b^)	11,082 (17.4)	10,515 (19.9)	7504 (17.3)	15,835 (16.6)	16,369 (16.0)	28,976 (9.3)

N: sample size; BMI: Body mass index.

a Values are **means** ± **standard deviations**.

b Percentages among N = 183,238 genotyped European ancestry UKB participants with all CII components available, who replied to the baseline questionnaire (2006–2010) and answered to the question if their primary job involved shift work (defined as work schedules outside 9am–5pm). Shift workers are those who answered with “sometimes”, “usually” or “always”. Participants who answered with “prefer not to answer” or “do not know” were excluded from this analysis.

### Genome-wide association studies in the UKB

**Fig. 1:**
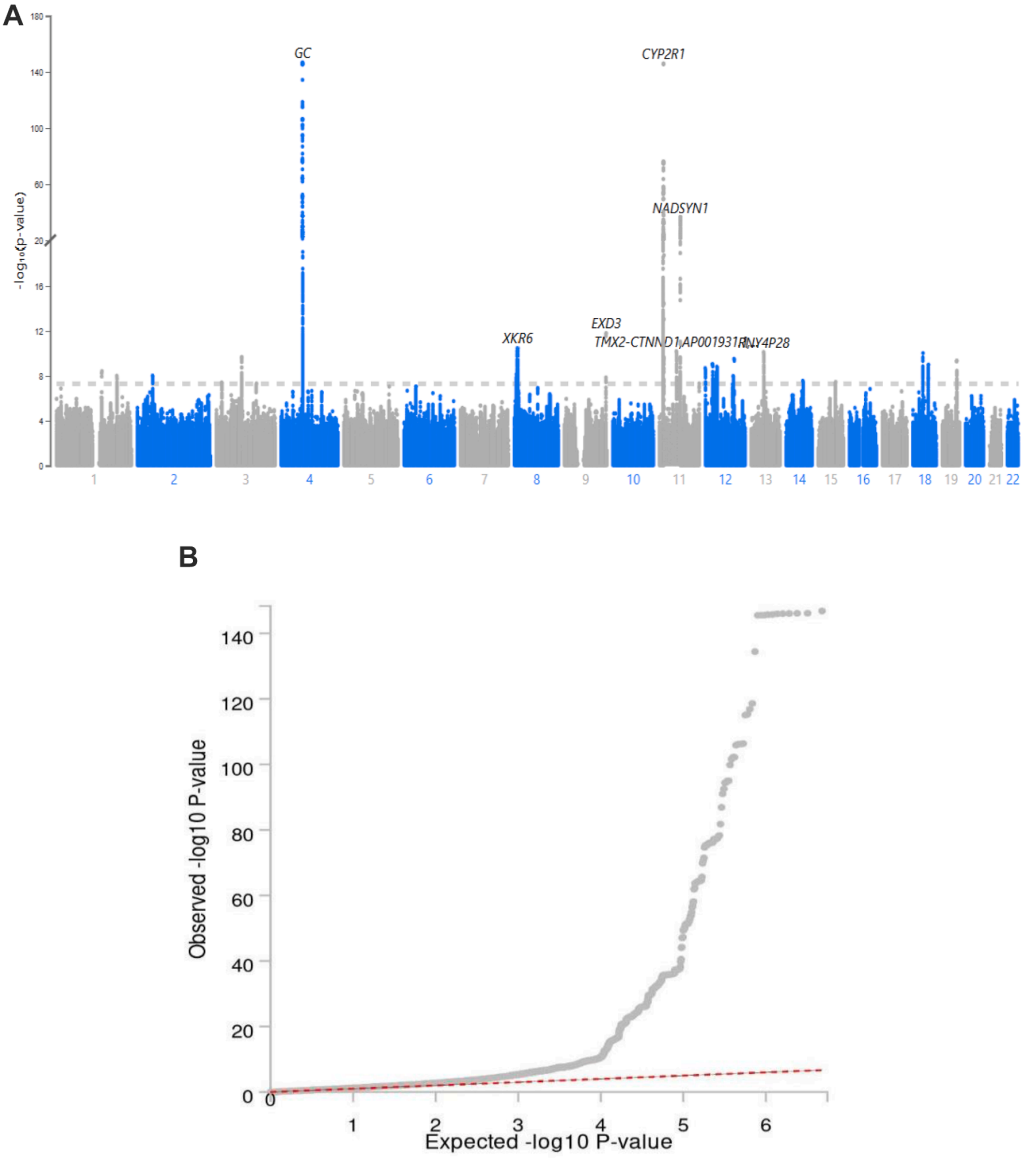
Manhattan plot (A) and quantile–quantile plot of expected versus observed log10P values (B) from of the genome-wide association study of the CII.

The Manhattan plot (Fig. 1A) and quantile–quantile (QQ) plot (Fig. 1B), generated using LocusZoom,^41^ illustrate GWAS results of the CII. We observed moderate inflation of the test statistics (λ_GC_ = 1.27, mean χ^2^ = 1.34). LD-score regression yielded SNP based heritability of h_SNP_^2^ = 0.066 (SE 0.0033; Supplementary Table S1) and an intercept of 1.01 (SE 0.0076), suggesting that inflation was predominantly due to polygenicity rather than confounding^35^ (attenuation ratio 0.03).

#### Functional consequences of genes

Functional annotation of all SNPs in linkage disequilibrium (LD; r^2^ ≥ 0.6) identified 27 genomic risk loci (Table 2). The most significant genomic loci were rs34265662 on chromosome 4 (p = 1.49E-147, β = 7.93E-02, SE = 0.003) near GC gene followed by rs117913124 on chromosome 11 (p = 1.01E-146; β = 0.22; SE = 0.009) near CYP2R1. Other significant genomic loci were mapped to e.g., NADSYN1 (p = 5.05E-37), EXD3 (p = 1.57E-12) as well as e.g., CELF4 (p = 9.28E-11) or SOX5 (p = 9.12E-10). A total of 72 genes with recognised Ensembl ID were mapped (Supplementary Table S2), with 5 circadian rhythm related genes (CRGs) among them (CALCA, DHCR7, KDM5A, HAL, and CRX; Table 3). The list of CRGs (Supplementary Table S3) was obtained from MSigDB database.^42^, ^43^, ^44^

**Table 2:** Genomic risk loci resulting from FUMA analysis based on the GWAS of the CII among 312,935 European ancestry UK Biobank participants.

Genomic Locus	Variant	Chromosome: position	Nearest gene	Effect allele	Effect size (β)	Standard error	P-value
7	rs34265662	4:72617557	GC	TA	0,0793	0,0031	1,49E-147
11	rs117913124	11:14900931	CYP2R1	A	0,2192	0,0085	1,01E-146
14	rs3794060	11:71187679	NADSYN1	T	−0,0434	0,0034	5,05E-37
10	9:140259068_T_TA	9:140259068	EXD3	TA	0,0301	0,0043	1,57E-12
9	rs73198970	8:11040216	XKR6	C	0,0187	0,0028	3,47E-11
12	11:57522200_CCCCT_C	11:57522200	TMX2-CTNND1:RP11-691N7.6:CTNND1	C	0,0196	0,0030	6,42E-11
22	rs567979837	13:60753105	RNY4P28	CT	−0,0185	0,0028	7,51E-11
25	rs1557341	18:35127427	CELF4	C	−0,0193	0,0030	9,28E-11
5	rs7652808	3:85603643	CADM2	G	0,0185	0,0029	2,12E-10
21	rs10859995	12:96375682	HAL	C	0,0178	0,0028	3,08E-10
27	rs2547244	19:48363039	TPRX2P	G	0,0231	0,0037	4,92E-10
17	rs6487365	12:24066460	SOX5	A	0,0183	0,0030	9,12E-10
26	rs2126786	18:53447005	RP11-397A16.1:RP11-397A16.2	C	0,0225	0,0037	1,01E-09
19	12:39098718_GT_G	12:39098718	CPNE8	G	0,0171	0,0028	1,55E-09
16	rs10848644	12:365,289	SLC6A13:RP11-283I3.4	T	−0,0167	0,0028	2,81E-09
1	rs61816761	1:152285861	FLG-AS1:FLG	A	−0,0574	0,0097	3,80E-09
8	rs2979241	8:8303353	CTA-398F10.2	C	−0,0162	0,0028	7,34E-09
3	rs11563179	2:51693002	AC007682.1	C	0,0222	0,0039	9,43E-09
2	rs2279681	1:201861016	SHISA4	G	−0,0168	0,0029	1,02E-08
20	rs139923919	12:93222395	EEA1	CAT	−0,0176	0,0031	1,04E-08
13	rs509533	11:66070575	TMEM151A	C	0,0159	0,0028	1,68E-08
23	rs16661	14:75128316	AREL1	C	0,0165	0,0030	2,74E-08
18	rs9668760	12:34611172	RP13-7D7.1	G	−0,0157	0,0028	2,98E-08
24	rs62007299	15:77711719	PEAK1	A	0,0169	0,0031	3,55E-08
4	rs7625384	3:18774357	AC144521.1	T	0,0170	0,0031	3,89E-08
6	rs4082244	3:134729537	EPHB1	G	−0,0160	0,0029	4,79E-08
15	rs10750539	11:133548873	RP11-448P19.1	A	0,0160	0,0029	4,81E-08

**Table 3:** Circadian rhythm related genes resulting from FUMA analysis based on the GWAS of the CII among 312,935 European ancestry UK Biobank participants.

Ensg	Symbol	Chr	Strand	MsigDB label	PLI	Min GWAS p	Ind Sig Snps	GL
ENSG00000110680	CALCA	11	−1	WP CLOCKCONTROLLED AUTOPHAGY IN BONE METABOLISM^a^	0.0014	4.8730e-25	11:14992795_AGGAGCCCACAGACCTT_A; rs61878675	11
ENSG00000172893	DHCR7	11	−1	UEDA PERIFERAL CLOCK^b^	5.0427e-08	6.1235e-37	rs12793607, rs1540129, rs3794060, rs1790349, rs369124946, 11:71203933_AT_A, rs181766110, rs139168803, rs35708376, rs760165576	14
ENSG00000073614	KDM5A	12	−1	WP CIRCADIAN RHYTHM GENES^c^	0.99999	6.1331e-07	rs10848644	16
ENSG00000084110	HAL	12	−1	UEDA PERIFERAL CLOCK^b^	5.9087e-17	3.0841e-10	rs10859995	21
ENSG00000105392	CRX	19	1	WP CIRCADIAN RHYTHM GENES^c^	0.6237	4.7685e-08	rs2547244	27

Ensg: Ensembl gene identifier; Chr: chromosome; MsigDB Label: Molecular Signatures Database label; PLI: probability of being loss-of-function intolerant; Min GWAS p: minimum p-value from GWAS analysis; Ind Sig Snps: Independent significant SNPs; GL: Genomic Loci.

a Systematic name: M45523. MSigDB url: https://www.gsea-msigdb.org/gsea/msigdb/human/geneset/WP_CLOCKCONTROLLED_AUTOPHAGY_IN_BONE_METABOLISM.

b Systematic name: M12498; MSigDB url: https://www.gsea-msigdb.org/gsea/msigdb/human/geneset/UEDA_PERIFERAL_CLOCK; PMID: 15273285.

c Systematic name: M39605; MSigDB url: https://www.gsea-msigdb.org/gsea/msigdb/human/geneset/WP_CIRCADIAN_RHYTHM_GENES.

**Fig. 2:**
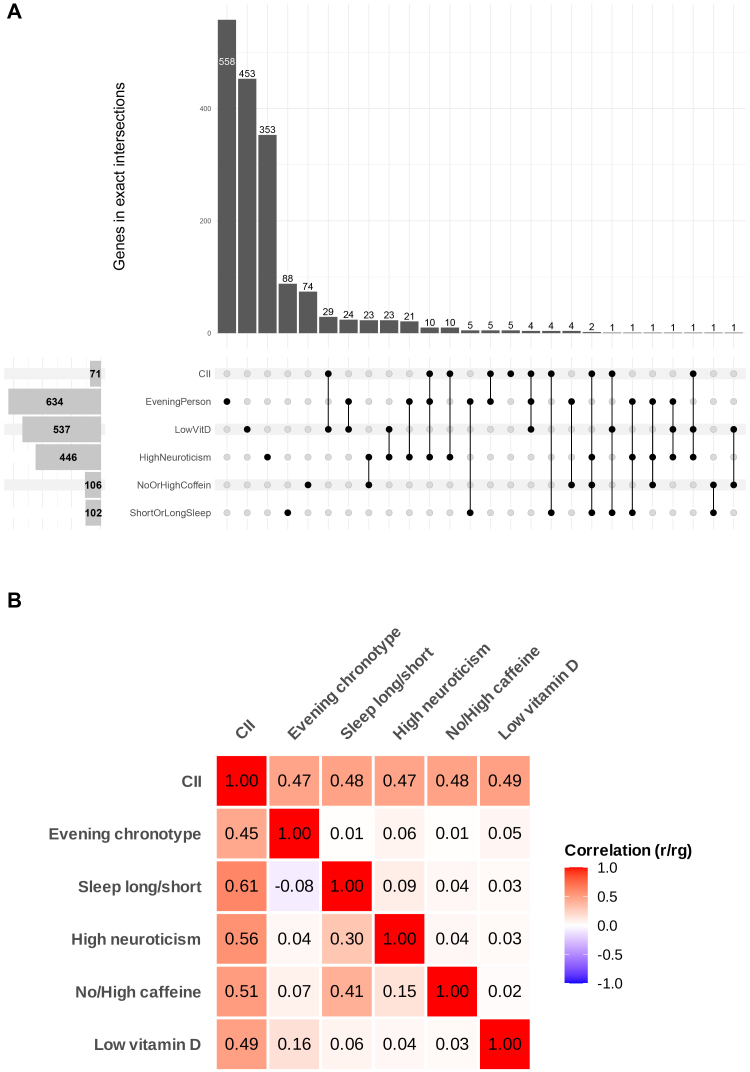
Relationship of the CII with its five component traits. (A) UpSet plot showing overlaps among mapped genes for CII and its five component traits. The left panel indicates the total number of mapped genes for each phenotype. The upper bar plot shows the number of genes in each exact, mutually exclusive intersection, and the connected dots below each bar indicate the phenotype sets contributing to that intersection. (B) Phenotypic (upper triangle) and genetic (lower triangle) correlations between the CII and its five components.

Analysis of gene-set overlap between the CII and its components showed that the exact pairwise intersections included 29 genes shared only by CII and low vitamin D levels, 10 shared only by CII and high neuroticism, 5 shared only by CII and evening chronotype and 4 shared only by CII and short or long sleep duration (Fig. 2A). There were no genes shared only by CII and no or high coffee consumption. In addition, five genes—EPHB1, SERPING1, C12orf74, PLEKHG7, and EEA1—were detected in the CII analysis but not in the component-specific mapped-gene results (Table 4). We also assessed pairwise gene overlap among the five CII components (Supplementary Table S4) and genes shared by at least two of the CII components (Supplementary Tables 5 and 6). Overlap was generally modest, with the largest intersections observed for evening chronotype with high neuroticism (34 genes), evening chronotype with low vitamin D (29 genes), no/high caffeinated coffee with high neuroticism (26 genes), and low vitamin D with high neuroticism (25 genes), indicating partial but limited shared genetic architecture among components.

**Table 4:** Genes significantly associated with the CII but not with any of its individual components, obtained from FUMA analysis based on the GWAS of the CII among 312,935 European ancestry UK Biobank participants together with effect sizes (β), standard errors (se) and p-values (Ind Sig SNP p) for the corresponding independent significant SNPS in an overall analysis (GWAS overall) and among N = 164,462 European ancestry UK Biobank women (GWAS in women).

Ensg	GL	Symbol	Chr	PLI	Ind Sig Snps	GWAS overall			GWAS in women		
						Ind Sig SNP p	β	se	Ind Sig SNP p	β	se
ENSG00000154928	6	EPHB1^a^^,^^b^	3	9.98E-01	rs4082244	4.79E-08	−0.0160	0.0029	1.47E-07	−0.0303	0.0058
ENSG00000149131	12	SERPING1^a^^,^^b^^,^^d^	11	9.73E-01	rs1647396	2.46E-07	0.0153	0.0028	–	–	–
ENSG00000214215	20	C12orf74^a^^,^^b^	12	5.22E-04	rs139923919	1.04E-08	−0.0176	0.0031	5.13E-06	0.0178	0.0039
ENSG00000187510	20	PLEKHG7^a^^,^^b^	12	3.68E-11	rs139923919						
ENSG00000102189	20	EEA1^a^^,^^c^	12	6.97E-01	rs139923919						

Ensg: Ensembl gene identifier; Chr: chromosome; MsigDB Label: Molecular Signatures Database label; GL: Genomic Loci; PLI: probability of being loss-of-function intolerant; Ind Sig Snps: Independent significant SNPs; Ind Sig SNP p: p-value for Ind Sig SNP.

a Protein coding.

b Forward strand.

c Reverse strand.

d Gene did not appear in women-specific FUMA results.

Tissue expression analysis performed in MAGMA indicated expression in brain cerebellum, frontal cortex and nucleus accumbens (Supplementary Fig. S1) and gene set analysis indicated significant curated gene sets M39352 (vitamin D metabolism; adjusted p-value 0.0326) and M1493 (Nikolsky breast cancer 11q12_q14 amplicon; adjusted p-value 0.0492; Supplementary Table S7). Among GWAS catalogue recognised gene sets, some were associated with CII components (Vitamin D levels and insufficiency, neuroticism, sleep duration and chronotype Supplementary Table S4). In addition, CII was associated with GWAS catalogue gene sets linked to psychiatric and neurodevelopmental disorders, cardiometabolic traits, immune-related conditions, behavioural tendencies, and lifestyle factors (Supplementary Table S7).

### Phenotypic and genetic correlations between the CII and its components

To further evaluate the validity of the CII, we examined its phenotypic and genetic correlations with its individual components - and among the individual components themselves–within our study sample. Phenotypically, CII showed a consistent moderate to strong positive correlation with each of the component traits (Fig. 2B). Genetic correlations were uniformly stronger than the phenotypic correlations (Fig. 2B), suggesting that shared genetic factors contribute to the relationship between the CII and its components. The strongest genetic associations were observed for high neuroticism (r_g_ = 0.56, p = 4.79 × 10^−143^) and short or long sleep duration (r_g_ = 0.61, p = 1.0 × 10^−117^), while caffeine intake (r_g_ = 0.51, p = 4.33 × 10^−53^) and low vitamin D levels (r_g_ = 0.49, p = 5.92 × 10^−82^) also exceeded their phenotypic counterparts. Evening chronotype showed a slightly lower but still strong genetic correlation (r_g_ = 0.45, p = 4.73 × 10^−88^). This suggests that CII accounts for a substantial portion of the shared genetic susceptibility across component traits, beyond what can be observed at the phenotypic level. We also evaluated phenotypic correlations between the CII and objective accelerometry-derived measures of circadian stability in the UK Biobank. Higher CII was modestly inversely correlated with Sleep Regularity Index (SRI)^45^ and Relative Amplitude (RA) (r = −0.10 and −0.12, respectively).

### Genetic-variant—shift work GWAS results

To test whether associations of genetic variants with CII differ by shift work status we conducted a genetic-variant-by-shift work GWAS. Using the default parameters of FUMA we identified 91 GW-significant (p < 5 × 10^−8^) interaction lead variants (Supplementary Table S8). After conducting separate shift-work stratified GWASs (Supplementary Fig. S2), we checked effects of interaction lead variants among shift workers (N = 28,976) and no-shift workers (N = 154,262) (Supplementary Table S8). Since the shift workers (SW) subgroup contained substantially fewer individuals than the no shift workers (noSW), SW-specific effect estimates had larger standard errors, reducing statistical power to detect the interaction. Across the 91 lead variants, interaction p-values were mostly similar to those in the noSW stratum (Supplementary Fig. S3), and the estimated differences in effects were small with wide confidence intervals in SW (Supplementary Table S8, Supplementary Fig. S4–S7), suggesting that the lack of interaction significance largely reflects limited precision in the smaller SW stratum rather than strong evidence against effect modification. Variants with both opposite effect size directions and heterogeneity p-values that remain significant after multiple-testing correction (α = 0.05/91 = 5.5 × 10^−4^), were considered as indicating SW-specific effect modification. Of the 91 lead variants, two showed significant heterogeneity—rs79799991 (GL 21; 5:141914736:A:T; P_delta_ = 1.9 × 10^−4^; P_int_ = 9.6 × 10^−6^) close to FGF1 gene and rs7161954 (GL 57; 15:78118955:G:T; P_delta_ = 2.4 × 10^−4^; P_int_ = 1.9 × 10^−7^) close to LINGO1 gene—both indicating smaller effects in SW than noSW (Supplementary Table S8). Given the substantially smaller shift-worker subgroup compared with the non-shift-worker subgroup, we performed formal approximate power calculations under a linear SNP × shift-work interaction model using the observed sample sizes, shift-work prevalence, minor allele frequencies, and the observed standard deviation of the CII. The formal test for interaction had 80% power at α = 5 × 10^−8^ to detect only moderate effect sizes, ranging from 0.148 CII units for variants with MAF 0.05 to 0.070 CII units for variants with MAF of 0.30.

### Sex differences in genetic associations

We conducted genetic-variant-by-sex and sex-specific GWAS analyses for the CII in our study sample to investigate potential modification of genetic variants effects by sex (Supplementary Fig. S8–S14). Compared to the overall sample analysis, of the five CII-specific genes (Table 4), three (EPHB1, PLEKHG7 and EEA1) were also identified in women-specific FUMA results (Table 4) while none of them were found in men-specific analyses. When looking at corresponding independent significant SNPs for the five CII-specific genes, they are placed on three genomic loci (chr 3 (GL 6), chr 11 (GL 12) and chr 12 (GL 20); Table 4). Two of these three loci were also identified among women (chr 3:134729537:C:G (EPHB1) and chr12:93222395:C:CAT (EEA1)) with one (chr3:134729537:C:G; rs4082244) showing the same direction of effects in both overall (β = −0.0160, se = 0.0029) and women-specific analyses (β = −0.0303, se = 0.0058; Table 4).

Using default FUMA parameters, we identified 65 lead variants for SNP × sex interaction (Supplementary Table S9). Comparing sex-stratified GWAS effect sizes in men (N = 148,473) and women (N = 164,462) (Supplementary Table S10; Supplementary Fig. S9–S14), nine variants showed opposite effect directions and significant heterogeneity after multiple-testing correction (α = 0.05/65 = 7.7 × 10^−4^; Supplementary Table S11), indicating sex-specific effects. These mapped near genes implicated in lipid or glucose metabolism (PID1), coagulation and vessel repair (SERPINE1), inflammation (ALOX5AP), and growth signalling (NXN) (Supplementary Table S12). MAGMA tissue expression found no differentially expressed genes, but gene-set analysis revealed sex-dependent enrichments not seen in the overall GWAS, including blood pressure interaction with alcohol and smoking, body-shape indices, neurobehavioral and reproductive traits, brain structure, ocular and vascular phenotypes, autoimmune disease, and bone measures (Supplementary Table S13). Overlap with the main GWAS was primarily vitamin D and neuroticism-related signals, suggesting these are largely shared across sexes.

### PheWAS of the CII-PRS in a clinical and population-based biobanks

**Fig. 3:**
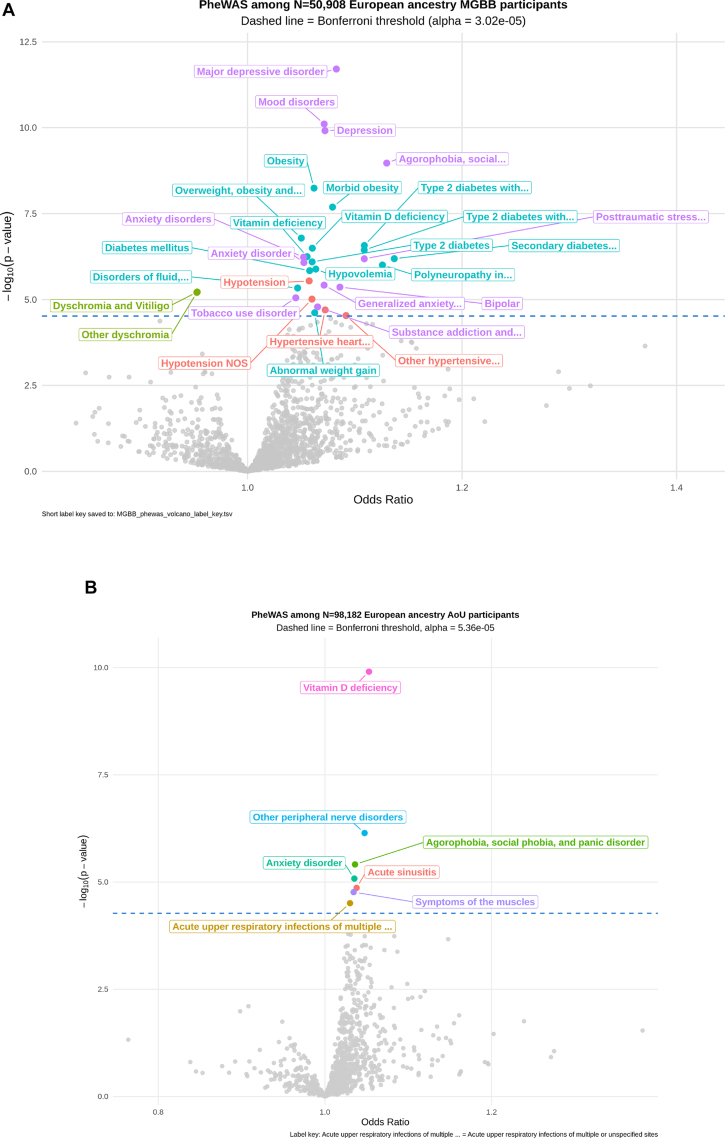
PheWAS results for the PRS-CS polygenic risk score of the Circadian Imbalance Index (CII-PRS) among European-ancestry participants in (A) the Mass General Brigham Biobank (MGBB; N = 50,908) and (B) All of US (AoU; N = 98,182). The x-axis shows the odds ratio per unit increase in the CII-PRS and the y-axis shows –log10 (p value). The dashed line indicates the Bonferroni-corrected significance threshold. Labelled points denote phenotypes exceeding this threshold, whereas grey points represent non-significant associations. In MGBB, the strongest signals were concentrated in psychiatric, metabolic, obesity-related, and hypertensive phenotypes. In AoU, the strongest signals included vitamin D deficiency, anxiety-related phenotypes, peripheral nerve disorders, acute sinusitis, upper respiratory infections, and muscle-related symptoms. Full results are provided in Supplementary Tables S14–S17.

We further searched for CII-associated phenotypes by conducting two PheWAS of a CII polygenic risk score (PRS) generated with PRS-CS.^37^ PRS weights were trained in UK Biobank and applied to European-ancestry participants in MGBB (N = 50,908) and All of US (AoU; N = 98,182). In MGBB, the CII-PRS was significantly associated with 31 disease phenotypes (p_MGBB_ <3.02E-5; Supplementary Table S14, Fig. 3A), with the strongest signals for endocrine/metabolic outcomes (multiple diabetes-related phenotypes), psychiatric disorders (phobias/panic disorder, PTSD, bipolar disorder, major depressive disorder), and hypertensive complications; inverse associations were observed for dyschromia and vitiligo. Sex-stratified MGBB analyses showed associations with obesity and mood- or anxiety-related traits (and skin cancer) in women, and angina pectoris/ischaemic heart disease in men (Supplementary Tables S15–S16; Supplementary Fig. S15). In AoU, seven phenotypes were significant (p_AoU_ < 5.36E-05; Supplementary Table S17, Fig. 3B), led by vitamin D deficiency, followed by peripheral nerve disorders, acute sinusitis, upper respiratory infections, and anxiety-related phenotypes.

In PheWAS sensitivity analyses using component-weighted PRSs, Bonferroni-significant associations were limited to a small number of component-related phenotypes. In MGBB (Supplementary Table S18; Supplementary Fig. S16), the vitamin D-weighted score was associated with vitamin D deficiency (OR = 1.08, 95% CI 1.06–1.11) and vitamin deficiency (OR = 1.07, 95% CI 1.05–1.09), and the no/high-caffeine-weighted score was associated with angina pectoris (OR = 1.05, 95% CI 1.03–1.08). In AoU (Supplementary Table S19; Supplementary Fig. S17), the vitamin D-weighted score was associated with vitamin D deficiency (OR = 4.08, 95% CI 3.23–5.14), and the neuroticism-weighted score was associated with anxiety disorder (OR = 2.05, 95% CI 1.49–2.81) and agoraphobia/social phobia/panic disorder (OR = 1.93, 95% CI 1.41–2.63). No Bonferroni-significant associations were observed for the remaining component-weighted scores. In contrast, the CII-PRS showed associations across a broader set of phenotypes, suggesting that the composite score captures an aggregate genetic signal within the CII-associated variant set rather than simply reproducing the strongest component-specific association.

### CII-associated phenotypes and genetic correlation analysis

**Fig. 4:**
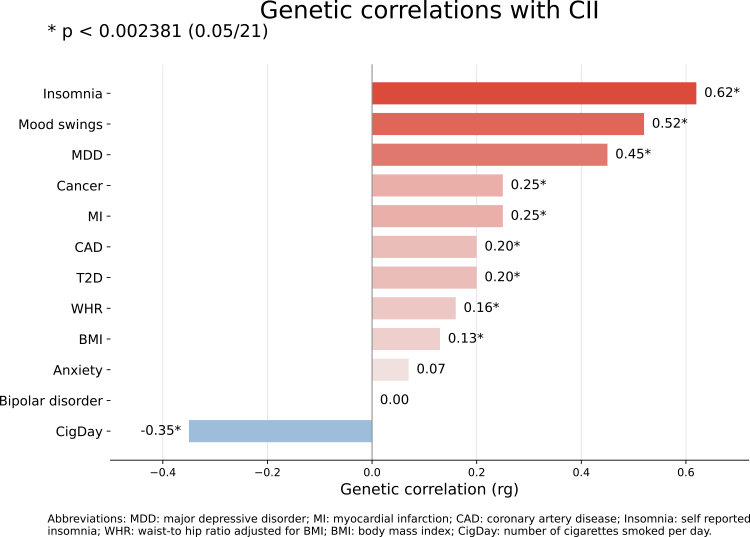
Genetic correlations between the CII and (disease) phenotypes of interest.

We compiled phenotypes of interest from FUMA outputs (GWAS-catalogue traits and gene sets; Supplementary Table S7) and PheWAS-significant diseases in MGBB and AoU (Supplementary Tables S14 and S17), excluding CII components and phenotypes with low heritability or case counts. Sixteen traits remained (Supplementary Table S20) for genetic-correlation analysis with CII using UKB-GWAS summary statistics, with multiple-testing correction (p < 0.0023). CII showed positive genetic correlations with insomnia, MDD, mood swings, MI, CAD, T2D, BMI, BMI-adjusted WHR, and borderline cancer, and a negative correlation with number of cigarettes smoked per day (Fig. 4; Supplementary Tables S21 and S22).

### Two sample Mendelian randomisation

We examined potential causal relationships between CII and the ten traits showing significant genetic correlation with CII (insomnia, mood swings, MDD, cancer, MI, T2D, CAD, BMI-adjusted waist-to-hip-ratio (WHRadjBMI), BMI, no. cigarettes). Summary statistics were obtained from FinnGen^46^ (insomnia, mood, cancer, MI, T2D, CAD, BMI), The Psychiatric Genomics Consortium (MDD^47^), GIANT (WHRadjBMI^48^) and Tobacco and Genetics Consortium (cigarettes per day^49^; Supplementary Table S23). We performed bi-directional two sample MR using GSMR.^38^, ^39^, ^40^ In the forward direction (CII being an exposure), higher genetically predicted CII was associated with increased odds of T2D (OR = 1.170; 95% CI = (1.053, 1.299)), MI (OR = 1.208; 95% CI = (1.029, 1.418)) and mood swings (OR = 1.129; 95% CI = (1.010, 1.261)) (Supplementary Table S24). We applied a conservative Bonferroni-corrected threshold of p < 0.0025 (0.05/20). Forward associations were nominally significant but did not meet this threshold and are therefore interpreted as suggestive. Instruments were genome-wide significant (P < 5 × 10^−8^) clumped for independence (LD r^2^ < 0.05), with horizontal pleiotropy addressed using the HEIDI-outlier filtering (P < 0.01). After filtering, 23 instruments were retained for CII→T2D and CII→mood, and 25 for CII→MI, with multi-SNP HEIDI-outlier P-values (0.106, 0.046, and 0.717, respectively) not suggesting pervasive pleiotropy (Supplementary Table S25 and S26). In the reverse direction, genetically predicted mood phenotype (β = 0.129, SE = 0.019, p = 8.32 × 10^−12^) and CAD (β = 0.015, SE = 0.005, p = 0.0017) remained significant after Bonferroni correction, whereas MI showed only nominal evidence of association with CII (β = 0.011, SE = 0.005, p = 0.0335), and T2D showed no evidence of association (β = −0.004, SE = 0.004, p = 0.344; Supplementary Table S25).

To further address the possibility of correlated pleiotropy, we performed MR-Egger and weighted median sensitivity analyses (Supplementary Table S27; Supplementary Fig. S18 and S19). These analyses supported the CII→MI association, with consistent positive effects in MR-Egger (β = 0.173; p = 0.035) and weighted median analyses (β = 0.180; p = 0.012), and no evidence of a significant MR-Egger intercept (p = 0.954). The CII→T2D association was directionally consistent across methods but reached nominal significance only in MR-Egger (β = 0.154; p = 0.030). In the reverse direction, mood→CII was robustly supported by both MR-Egger (β = 0.142; p = 2.64 × 10^−4^) and weighted median analyses (β = 0.152; p = 3.02 × 10^−4^), with no evidence of directional pleiotropy based on the MR-Egger intercept (p = 0.402). CAD→CII was also significant in both sensitivity models, but the MR-Egger intercept was significant (p = 0.032), suggesting possible directional pleiotropy.

### Replication in the Nurses’ Health Study II

For replication analysis we used 11,351 women data from the NHS2 cohort. Compared to UKB women, nurses in the NHS2 were on average younger (56.2 (8.0) in the UKB vs 55.39 (4.40) in NHS2) and had similar average BMI (27.0 (5.1) in the UKB vs 27.34 (6.22) in NHS2). In both cohorts, women with higher CII values were more likely to have higher BMI, lower socio-economic status and were more likely to perform shift work (Supplement 3, Supplementary Tables N2–N3). Building on our prior UKB findings^7^ that CII is associated with increased cardiovascular-kidney-metabolic (CKM) risk—especially when combined with night shift work among European-ancestry participants—we tested whether NHS2 reproduced these phenotypic patterns and whether cumulative shift-work duration modified CII–CKM associations.

In NHS2, CKM risk increased with higher CII, with a significant dose–response trend (p_trend_ <0.0001; Supplement 3, Supplementary Table N4). CKM risk also increased jointly with higher CII and longer shift-work duration, with significant trends within each CII stratum (low 0–1, middle 2–3, high 4–5; Supplement 3, Supplementary Tables N5–N7). Notably, the trend emerged using finer shift-work categories in NHS2 (never, <5 years, ≥5 years), whereas in UKB it was apparent only with broader categories (never, <20 years, ≥20 years), likely reflecting the larger number of shift-working nurses and higher-quality shift-work assessment in NHS2. Overall, these analyses support phenotypic replication of CII characteristics and associations observed in UKB.

Because NHS2 is women-only, GWAS replication focused on lead SNPs from the UKB women GWAS (N = 164,462). Of 104 genome-wide significant lead variants in UKB women, 84 were available in NHS2 after QC (Supplementary Table S28); ten showed nominal significance in NHS2 (p < 0.044; Supplementary Table S29), and four also had consistent effect directions (rs11917139 near ZBTB20, rs330905 near PPP1R3B, rs35358081 near OR1J1, rs10848644 near SLC6A13). Finally, a PRS trained in UKB (PRS-CS^37^) was associated with observed CII in NHS2 (N = 11,344) after adjustment for age and 10 genetic PCs, improving model fit (likelihood ratio χ^2^ = 16.3, p = 5.4 × 10^−5^), but explaining <0.2% additional variance.

#### Leave-one-component-out GWAS and MR sensitivity analyses

Leave-one-component-out GWASs showed that the main CII signal was not entirely driven by any single component (Supplementary Table S30; Supplementary Fig. S20–S25). Across the available comparisons, effect directions were consistent for the lead loci, with exclusion of vitamin D producing the greatest attenuation. When chronotype, sleep duration, neuroticism, or caffeine were omitted, 14, 19, 14, and 17 lead loci remained genome-wide significant, respectively, and all available loci remained nominally significant. Excluding vitamin D had the largest effect, particularly for the leading vitamin D-related loci rs34265662 near GC (Supplementary Fig. S21A), rs117913124 near CYP2R1 (Supplementary Fig. S21E), and rs3794060 near NADSYN1/DHCR7 (Supplementary Fig. S22B). Even so, 7 of 25 available lead loci remained genome-wide significant and 20 of 25 remained nominally significant after excluding vitamin D (Supplementary Table S30).

Notably, the loci highlighted as CII-specific also persisted across leave-one-out analyses. rs4082244 (Supplementary Fig. S20F), mapping to EPHB1, showed consistent negative effect estimates across all five leave-one-out GWASs (beta range −0.0138 to −0.0121; p range 9.71E-08 to 3.64E-06). rs139923919 (Supplementary Fig. S23B), mapping to C12orf74, PLEKHG7, and EEA1, also retained a consistent direction of effect across all available leave-one-out analyses (beta range −0.0153 to −0.0139; p range 2.51E-08 to 3.26E-07). These findings suggest that, although vitamin D-related biology contributes strongly to several of the top associations, the composite CII captures broader shared genetic signal across multiple components rather than being reducible to a single dominant trait.

We next performed leave-one-component-out MR analyses for the key forward associations with MI, T2D, and mood swings (Supplementary Table S31, Supplementary Fig. S25). For T2D, estimates remained positive and nominally significant after excluding chronotype (OR = 1.086, 95% CI 1.015–1.161, p = 0.016), sleep duration (OR = 1.078, 95% CI 1.012–1.149, p = 0.019), neuroticism (OR = 1.084, 95% CI 1.014–1.160, p = 0.018), or caffeine (OR = 1.093, 95% CI 1.021–1.171, p = 0.011). For mood swings, estimates remained positive after excluding chronotype (OR = 1.026, 95% CI 1.007–1.045, p = 0.006), sleep duration (OR = 1.023, 95% CI 1.005–1.042, p = 0.013), or caffeine (OR = 1.023, 95% CI 1.003–1.043, p = 0.023), but were attenuated after excluding neuroticism (OR = 1.017, 95% CI 0.998–1.036, p = 0.085). For MI, estimates remained directionally positive after excluding chronotype (OR = 1.062, 95% CI 0.932–1.209, p = 0.367), sleep duration (OR = 1.058, 95% CI 0.934–1.198, p = 0.374), neuroticism (OR = 1.144, 95% CI 1.000–1.307, p = 0.050), or caffeine (OR = 1.091, 95% CI 0.954–1.248, p = 0.205), but were mostly non-significant. When vitamin D was excluded, forward GSMR estimates could not be obtained because too few valid instruments remained after applying the same selection and filtering criteria; therefore, the contribution of vitamin D to the forward MR findings could not be formally assessed in this framework. Overall, these analyses suggest that the forward MR findings are not driven solely by any of the CII components, while component contributions appear outcome specific.

## Discussion

We investigated the genetic architecture of a newly developed CII, an empirically defined composite score designed to summarise susceptibility to circadian disruption across heterogeneous sleep, psychological, behavioural, and light-exposure-related domains. The observed correlation structure supports interpreting the CII as a cumulative susceptibility index across distinct but related domains, rather than as a single latent construct or a direct measure of circadian phase. Thus, the CII complements component-specific analyses by summarising shared genetic susceptibility across partially independent traits that may contribute to vulnerability to circadian disruption. In the GWAS analysis of the CII we identified 27 genomic risk loci and 72 associated genes, including five previously known to be involved in circadian regulation (CALCA, DHCR7, KDM5A, HAL, and CRX). The strongest signals mapped to loci involved in vitamin D biology (GC, CYP2R1), metabolic and endocrine pathways (PDE3B, PSMA1, RRAS2),^50^ neurovascular signalling and pain (CALCA, CALCB),^51^^,^^52^ cellular maintenance (COPB1, PSMA1) and cholesterol and Vitamin D metabolism (NADSYN1, DHCR7).^53^^,^^54^ Although five genes (EPHB1, SERPING1, C12orf74, PLEKHG7, EEA1) were identified in the CII analysis but not in the component-specific analyses, these findings should be interpreted cautiously, as they may reflect aggregated genetic effects across correlated component traits and the greater power of the composite phenotype, rather than uniquely novel circadian biology. For PLEKHG7 and SERPING1,^55^ available evidence provides only indirect links to circadian-relevant pathways, while direct evidence connecting EPHB1, C12orf74, and EEA1 to circadian regulation is currently lacking. These genes should therefore be viewed as candidate CII-associated loci requiring further functional validation, rather than as established circadian genes. EPHB1 is involved in neural development and immune cell maturation^56^, ^57^, ^58^ and has been implicated in cancer progression and chronic pain.^59^^,^^60^ C12orf74 has been described as an oncogene activating EGFR/AKT/mTORC1 signalling and linked to poor prognosis in several cancers, and has also been associated with reduced coronary artery disease risk.^61^ EEA1 has been connected to susceptibility to fungal infection, autoimmune disease (including lupus autoantigenicity), and neurological disorders.^62^, ^63^, ^64^ The generally modest gene overlap among the individual CII components further supports the view that the index captures shared susceptibility across distinct domains, rather than a single tightly unified phenotype.

We identified 65 variants with sex-specific associations, including nine with opposite directions in men and women, highlighting metabolism, vascular biology, inflammation, and cell signalling pathways. We also performed exploratory SNP × shift-work analyses to assess whether occupational circadian stress might modify genetic associations with the CII. Although 91 interaction lead variants were detected, the substantially smaller shift-worker subgroup limited precision, and most effect differences were small. In practical terms, this analysis was powered mainly to detect moderate SNP × shift-work effects, and weaker interactions may therefore have gone undetected. Only two variants—rs79799991 near FGF1 and rs7161954 near LINGO1—showed significant heterogeneity after multiple-testing correction, with smaller effects in shift workers. FGF1 is central to metabolic regulation; disrupted FGF1/FGFR1 signalling impairs insulin processing and promotes diabetes-like phenotypes,^65^ and FGF1 is also implicated in tumour biology and adipose remodelling.^66^ LINGO1 is important for CNS development and has been linked to essential tremor and Parkinson's disease. Overall, these findings provide stronger support for sex-specific genetic effects than for modification by shift-work status, which will require confirmation in larger studies of shift-working populations.

In our women-only replication cohort (NHS2), the CII-PRS was significantly associated with observed CII, however, it explained less than 0.2% of additional variance, indicating that its predictive utility is currently limited and that these findings should be interpreted primarily as evidence of a modest but detectable polygenic signal.

Across two independent resources, a hospital-based biobank (MGBB) and a population cohort (AoU), the CII-PRS showed phenome-wide associations across neuropsychiatric, metabolic, and cardiometabolic domains. In MGBB, the strongest signals involved endocrine and metabolic traits (including T2D and hypertensive complications) and mental disorders (including MDD and mood-related phenotypes). AoU showed associations with anxiety-spectrum diagnoses, peripheral nerve disorders, musculoskeletal symptoms, acute sinusitis, and upper respiratory infections. Sex-stratified analyses in MGBB suggested heterogeneity: women showed associations with obesity, mood/anxiety disorders, and skin cancer, whereas men showed ischaemic phenotypes (angina pectoris, ischemic heart disease), potentially reflecting sex-specific circadian genetic risk and hormone-related immune regulation. Differences between MGBB and All of Us PheWAS results may partly reflect cohort design and phenotype ascertainment, as MGBB is a hospital- and patient-based biobank whereas All of Us is a broader population-based cohort. Therefore, differences in disease prevalence, healthcare utilisation, and EHR coding practices may contribute to heterogeneity in the specific phenotypes associated with the CII-PRS. Component-weighted PheWAS sensitivity analyses further suggested that individual components showed narrower, component-related associations, whereas the full CII-PRS captured a broader phenome-wide pattern, supporting its role as an aggregate genetic susceptibility measure rather than a simple proxy for a single component.

The PheWAS findings were broadly consistent with genetic correlation and MR analyses, which showed shared genetic architecture between CII and insomnia, mood swings, MDD, WHR, T2D, CAD, and MI. In MR analyses, forward associations of CII with T2D, mood swings, and MI were nominally significant but did not survive Bonferroni correction and should be considered suggestive. Reverse associations of mood swings and CAD with CII remained significant after correction, although CAD→CII showed evidence of possible directional pleiotropy in MR-Egger analyses. MR-Egger and weighted median analyses were directionally consistent with CII→MI and mood→CII, and provided partial support for CII→T2D. Overall, these results are compatible with potential directional links between CII and mood and cardiometabolic traits, but they should be interpreted cautiously because MR cannot fully distinguish effects of the composite CII from pathways operating through individual components, correlated pleiotropy, or feedback between circadian-related behaviours and disease.

The leave-one-component-out analyses further supported the robustness of the composite CII signal: although exclusion of vitamin D substantially attenuated several leading loci, particularly known vitamin D-related signals, associations at the CII-specific loci rs4082244 (EPHB1) and rs139923919 (C12orf74/PLEKHG7/EEA1) remained directionally consistent and statistically robust across leave-one-out analyses, arguing against these signals being driven by any single component alone. Leave-one-component-out MR analyses similarly suggested that the forward MR findings were not driven solely by any of the components, although component contributions were outcome-specific, with neuroticism contributing to the mood-related signal and vitamin D contributing to instrument strength.

We evaluated the genetic architecture of the CII, a composite measure of susceptibility to circadian disruption integrating sleep, circadian, light-exposure–related, behavioural, and psychological domains. Limitations include restriction to European ancestry, reliance on self-report for most components (except vitamin D), dichotomisation of component traits, limited shift-worker sample size, a women-only replication cohort (N = 11,344), and potential heterogeneity and coding bias in EHR-based PheWAS phenotypes. Most CII components were self-reported and may therefore be affected by recall error and reporting bias which would be expected mainly to attenuate associations, weaken genetic signal, and reduce precision. In addition, dichotomisation of continuous traits may have reduced information and statistical power, increased sensitivity to the selected cut-points, and obscured nonlinear or dose–response relationships. Accordingly, the present CII should be viewed as a practical composite measure of circadian susceptibility, rather than as a comprehensive representation of the underlying biology. In addition, the current CII does not incorporate objective, behaviourally derived measures that may better capture circadian regulation. An important goal of future research will therefore be to refine the CII by incorporating accelerometer-derived indices, such as the SRI^45^ and RA, as well as model-informed indicators of alignment with individualised optimal sleep timing.^67^ The modest inverse correlations of the CII with SRI and RA in the UK Biobank accelerometry subset support the view that the CII is related to, but not interchangeable with, objective measures of circadian stability. Future studies should examine whether the loci identified for the CII, as well as the CII PRS, are also associated with objective accelerometry- or wearable-derived measures of circadian stability and whether these measures share genetic architecture with the CII. A further goal will be to integrate static measures of circadian susceptibility, such as the CII or CII-PRS, with longitudinal wearable-derived and mathematically modelled biomarkers of sleep and circadian timing,^68^^,^^69^ thereby linking inherited risk with dynamic physiological states and potentially improving personalised prediction and intervention. Although the present study focused on the genetics of the original CII, defined as an unweighted index based on five dichotomised components, alternative formulations may improve representation of the multidimensional structure of circadian susceptibility. In particular, future studies should investigate PCA-based, other factor-based, weighted, and continuous-component versions of the CII. Genetic analyses of these refined indices will be important to assess the robustness of the current findings and to improve biological interpretation of the CII and assess the robustness of the current findings.

In conclusion, the CII summarises selected heterogeneous domains of susceptibility to circadian disruption and identifies genetic signals spanning sleep, psychiatric, metabolic, and cardiovascular domains, including signals modified by sex and, more cautiously, shift-work exposure. These findings align with the circadian syndrome concept,^70^ which links circadian rhythm disruption with cardiometabolic and related comorbidities. The results of the MR analysis suggest possible directional associations between the CII and mood as well as cardiometabolic traits, but they do not allow for a definitive determination of a causal relationship due to the complex nature of the CII, possible residual pleiotropy, and potential bidirectional interactions. With further validation, refinement, and integration with objective circadian measures, the CII may eventually contribute to research on risk stratification, although the current CII PRS has limited predictive performance and is not yet suitable for individual-level clinical use.

## Contributors

All authors read and approved the final version of the manuscript. MZ: conceptualisation, data curation, formal analysis, investigation, methodology, project administration, resources, software, visualisation, writing—original draft; MW: funding acquisition (AoU analyses), formal analysis (AoU), writing—review and editing; JZ: data curation (UKB), formal analysis, writing—review and editing; IS-L: methodology, writing—review and editing; LDM: methodology, writing—review and editing; AB: writing—review and editing; JV: formal analysis (MGBB), writing—review and editing; LV: formal analysis (All of US), writing—review and editing; SR, RR, MKR: conceptualisation, methodology, writing—review and editing; OO: conceptualisation, methodology (NHS2 replication), writing—review and editing; RS: conceptualisation, methodology, funding acquisition (MGBB), supervision, writing—review and editing; ESS: conceptualisation, funding acquisition, supervision, writing—original draft, writing—review and editing. MZ and MW accessed and verified the underlying data.

## Data sharing statement

The individual-level data underlying this article cannot be shared publicly due to participant consent, data protection requirements, and cohort-specific data access agreements. Researchers are encouraged to apply directly to the relevant data custodians for access to the underlying cohort data. UK Biobank data are available to approved researchers through the UK Biobank access process (https://www.ukbiobank.ac.uk/enable-your-research/apply-for-access). Data from other contributing cohorts and biobanks are governed by their respective access policies and cannot be redistributed by the authors. GWAS summary statistics generated in this study will be made publicly available in the GWAS Catalogue after publication. Analysis code used to generate the main results will be made available upon reasonable request to the corresponding author (magdalena.zebrowska@meduniwien.ac.at). Any code sharing will exclude individual-level cohort data and will be subject to the data-use restrictions of the contributing studies.

## Declaration of interests

MKR reports consulting fees from Eli Lilly and Company and modest stock ownership in GSK, outside the submitted work. S.R. reports institutional grants or contracts from Google/Proxima, consulting fees from Amgen Inc, unpaid board membership with the National Sleep Foundation and the Alliance of Sleep Apnoea Partners, and a stipend for an editor role at Sleep Health, all outside the submitted work. Other authors have no relevant financial or non-financial interests to disclose.
