# Supplementary material for: Genetic architecture of a Circadian Imbalance Index: genome-wide association, phenome-wide association, and Mendelian randomisation analyses
Source: eBioMedicine. 2026 Jul 21;130:106380. doi: 10.1016/j.ebiom.2026.106380 (PMC13476902; doi:10.1016/j.ebiom.2026.106380)
Supplement: Supplementary file 1 [file mmc4.pdf]

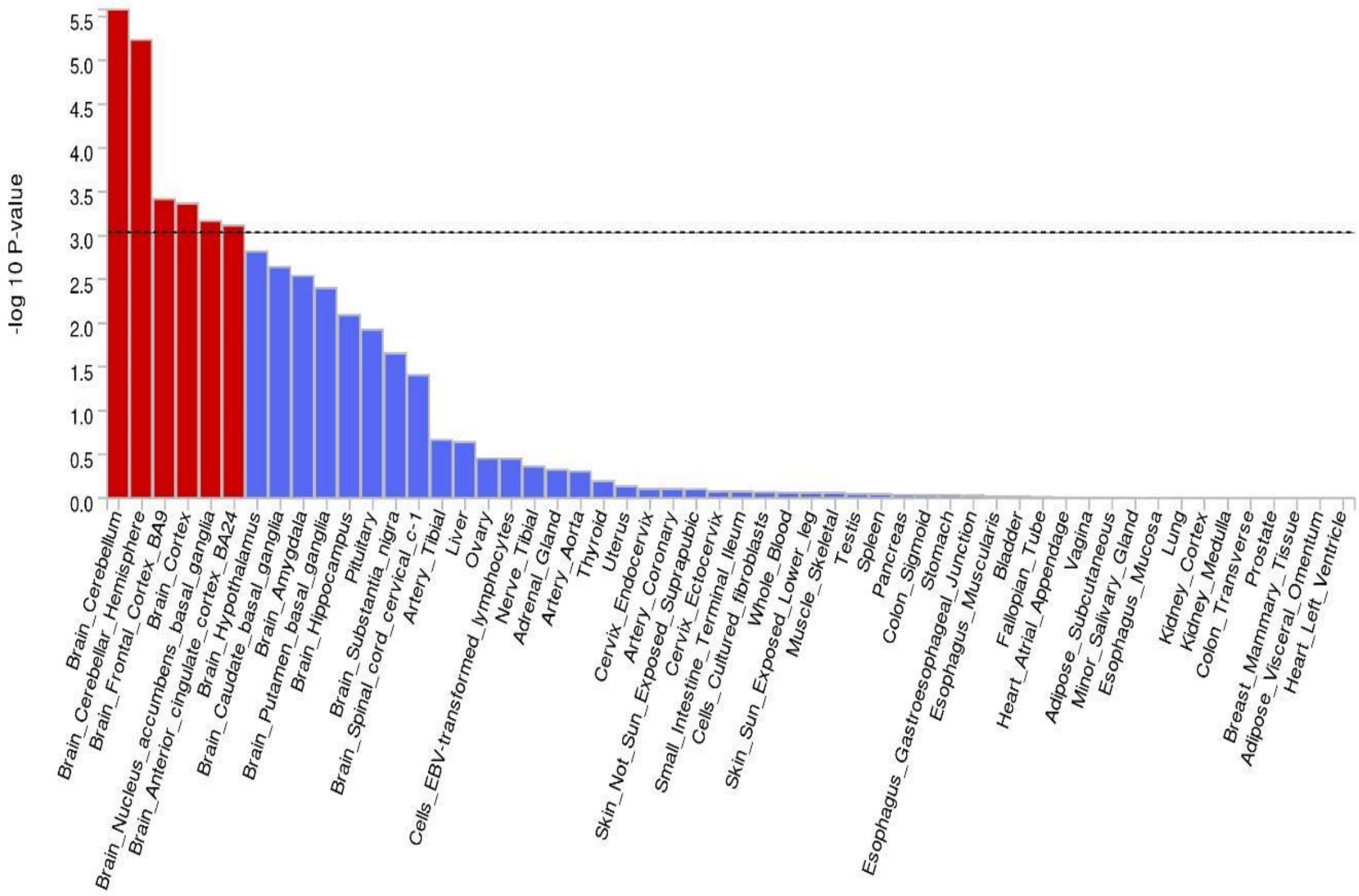

Supplementary Figure 1: MAGMA Tissue Expression analysis of the CII.

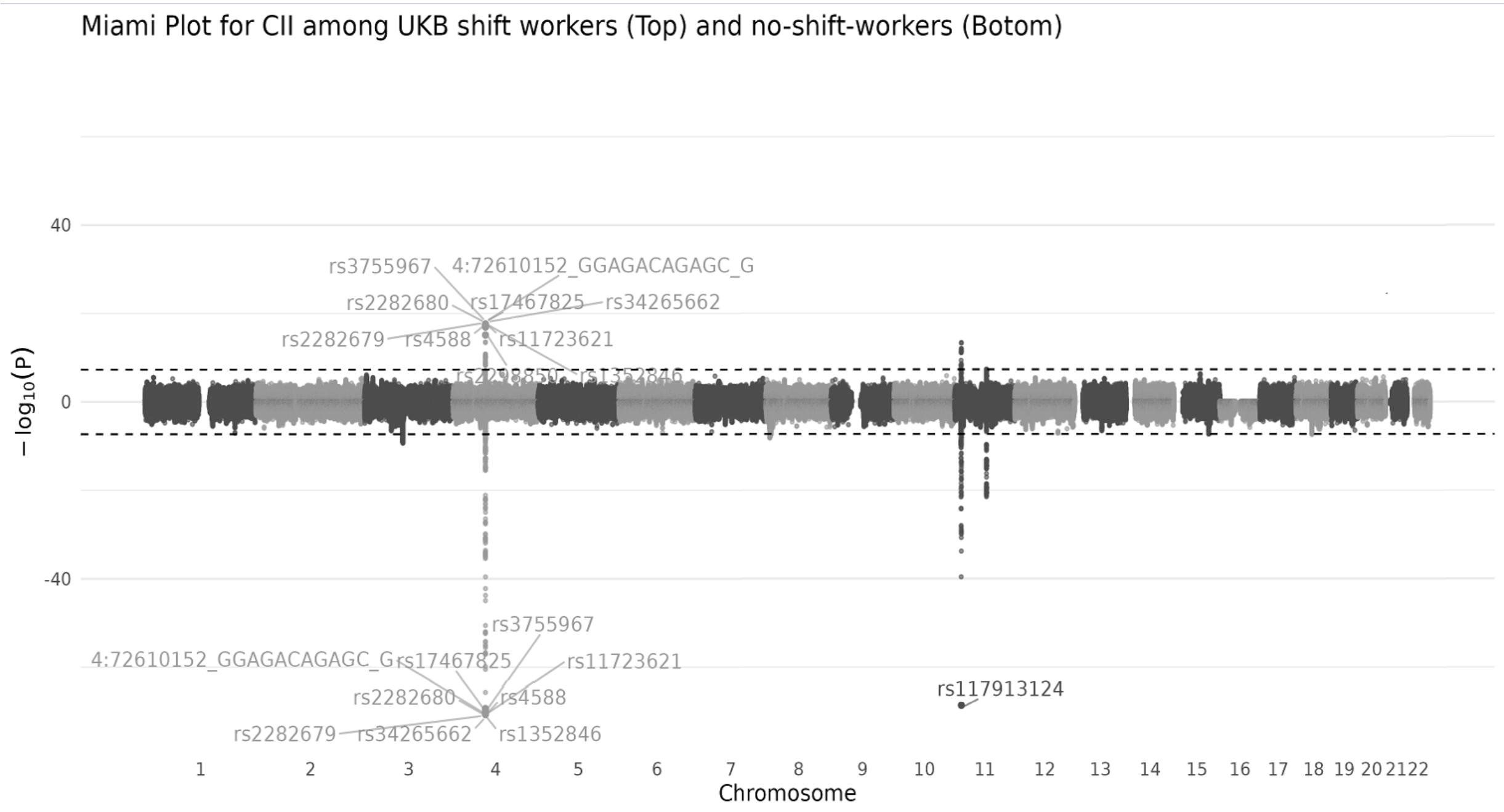

Supplementary Figure 2: Miami plot of CII-GWASs results for European ancestry UKB participants: shift workers (N=28,976; top) versus no-shift-workers (N=154,262; bottom).

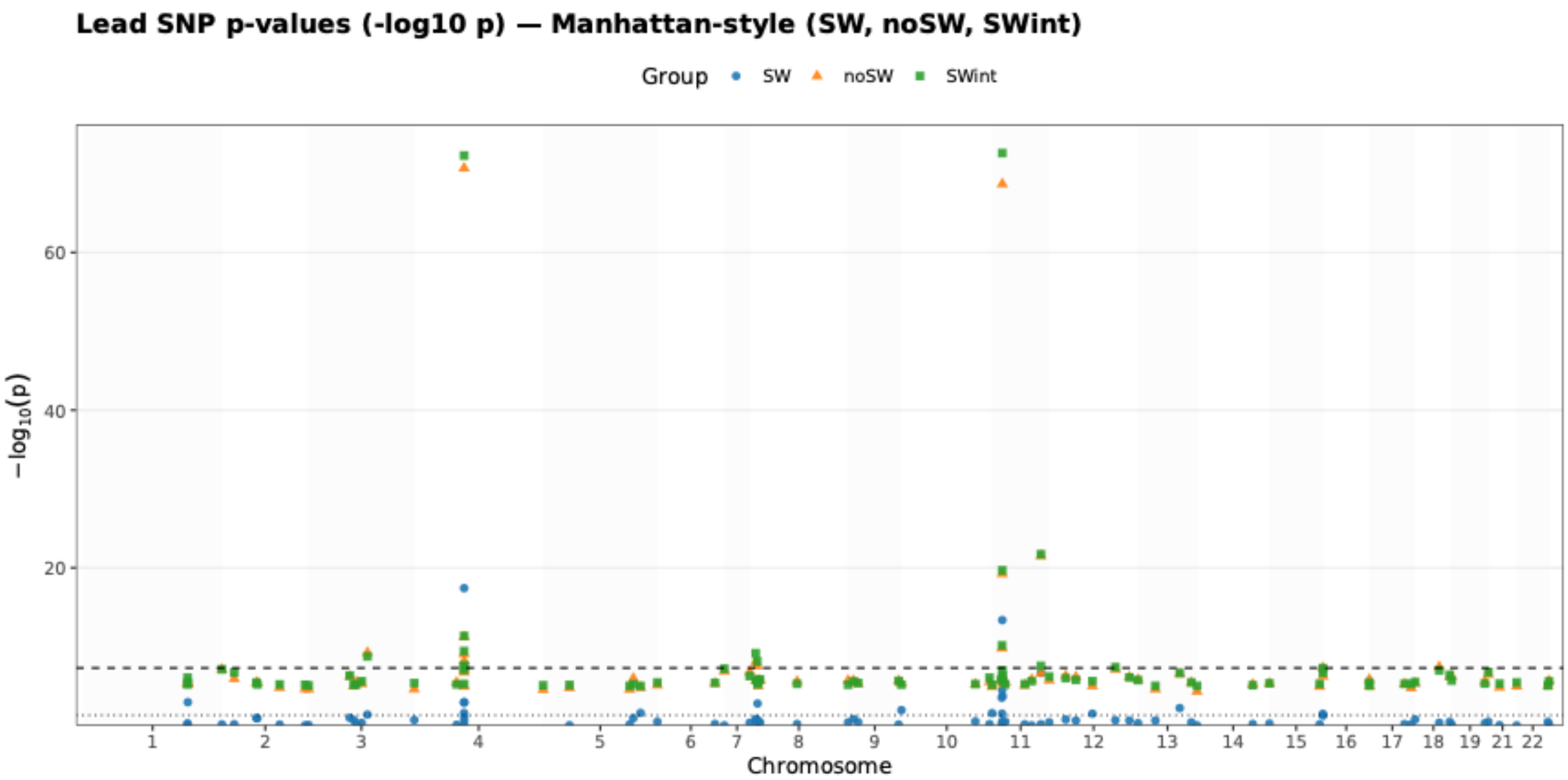

Supplementary Figure 3: Manhattan plot of –log10(p) values for lead SNPs from the SNP × shift work interaction (SWint) and corresponding results in shift workers (SW; N=28,976) versus no-shift-workers (noSW; N=154,262).

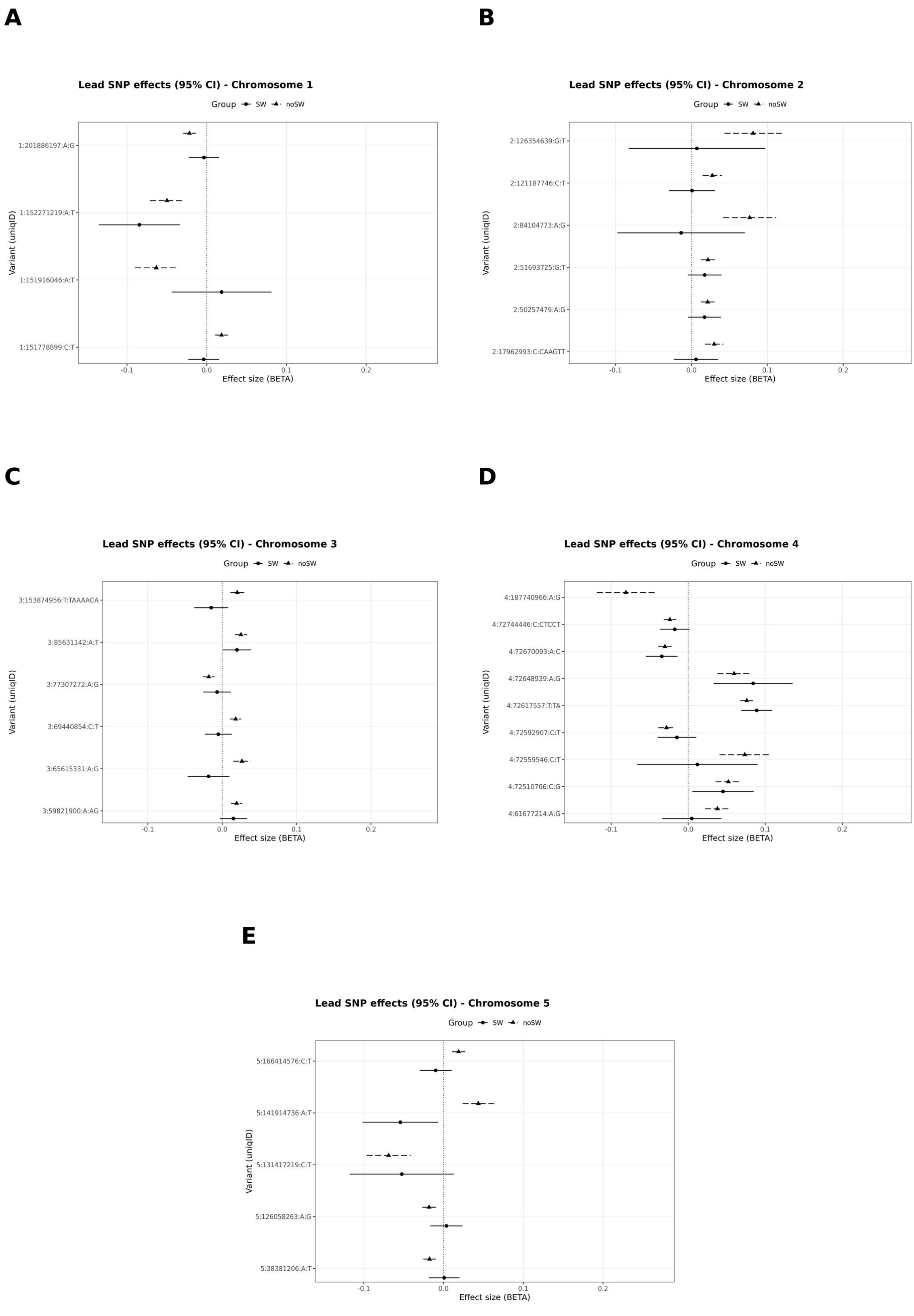

Supplementary Figure 4. Lead SNP effects by shift work status for chromosomes 1–5. Forest plots show lead SNP effect estimates and 95% confidence intervals for chromosomes 1–5. Panels show: A, chromosome 1; B, chromosome 2; C, chromosome 3; D, chromosome 4; and E, chromosome 5. Points represent beta effect estimates and horizontal lines represent 95% confidence intervals. Circles with solid lines indicate the shift work (SW) group, and triangles with dashed lines indicate the non-shift work (noSW) group. The vertical dotted line indicates a null effect estimate of zero.

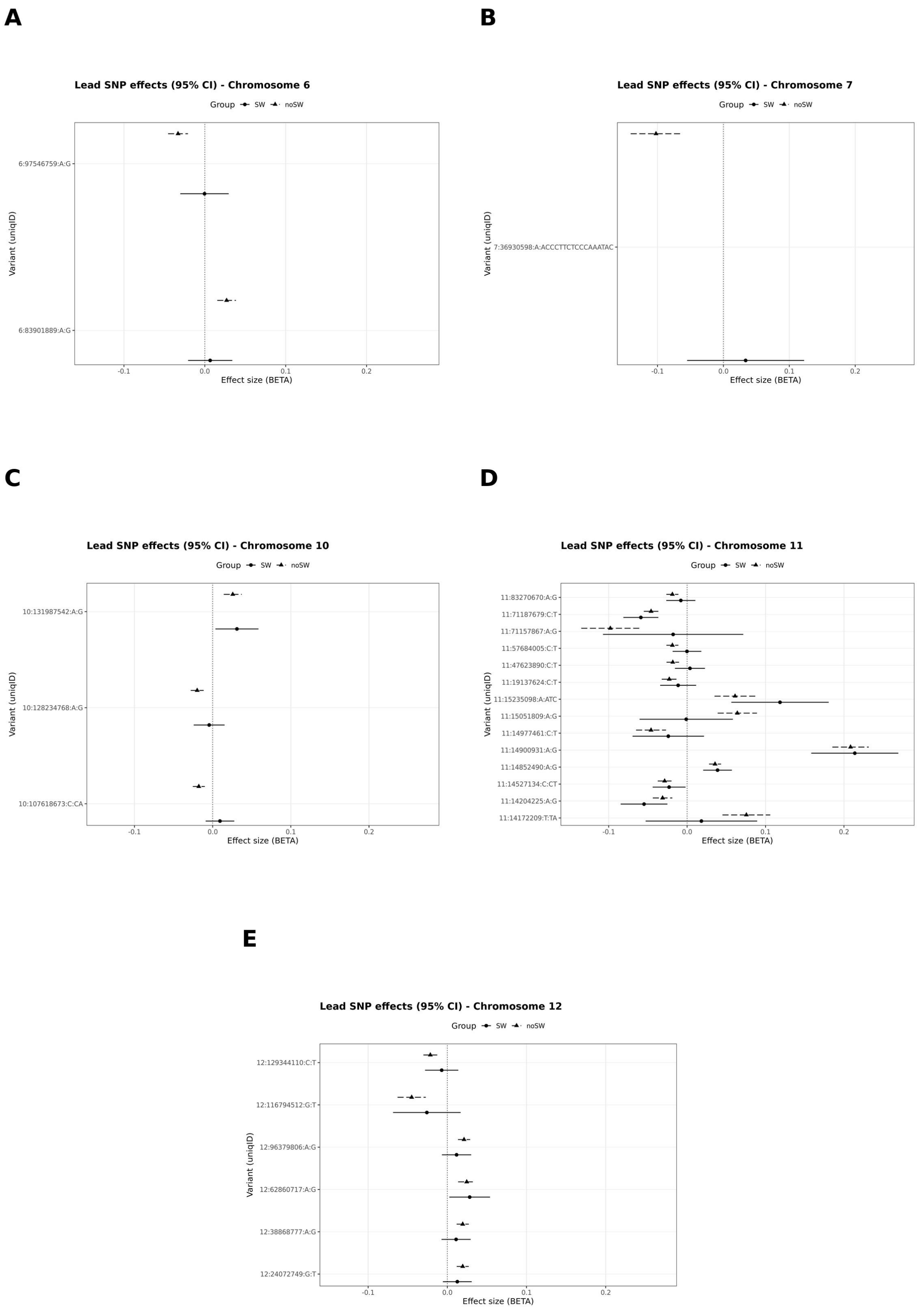

Supplementary Figure 5. Lead SNP effects by shift work status for chromosomes 6, 7, 10, 11, and 12. Forest plots show lead SNP effect estimates and 95% confidence intervals for chromosomes 6, 7, 10, 11, and 12. Panels show: A, chromosome 6; B, chromosome 7; C, chromosome 10; D, chromosome 11; and E, chromosome 12. Points represent beta effect estimates and horizontal lines represent 95% confidence intervals. Circles with solid lines indicate the shift work (SW) group, and triangles with dashed lines indicate the non-shift work (noSW) group. The vertical dotted line indicates a null effect estimate of zero.

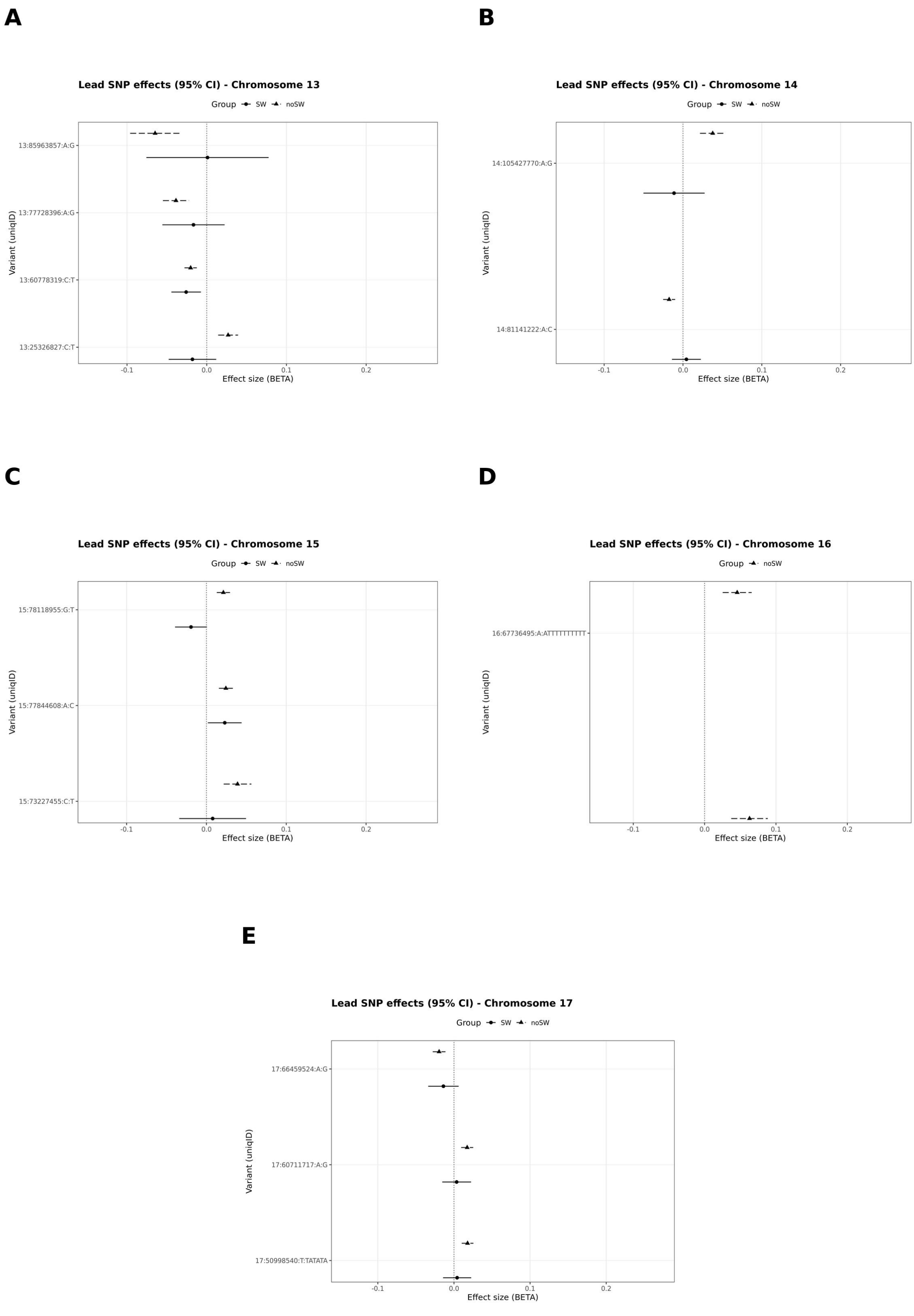

Supplementary Figure 6. Lead SNP effects by shift work status for chromosomes 13–17. Forest plots show lead SNP effect estimates and 95% confidence intervals for chromosomes 13–17. Panels show: A, chromosome 13; B, chromosome 14; C, chromosome 15; D, chromosome 16; and E, chromosome 17. Points represent beta effect estimates and horizontal lines represent 95% confidence intervals. Circles with solid lines indicate the shift work (SW) group, and triangles with dashed lines indicate the non-shift work (noSW) group. The vertical dotted line indicates a null effect estimate of zero.

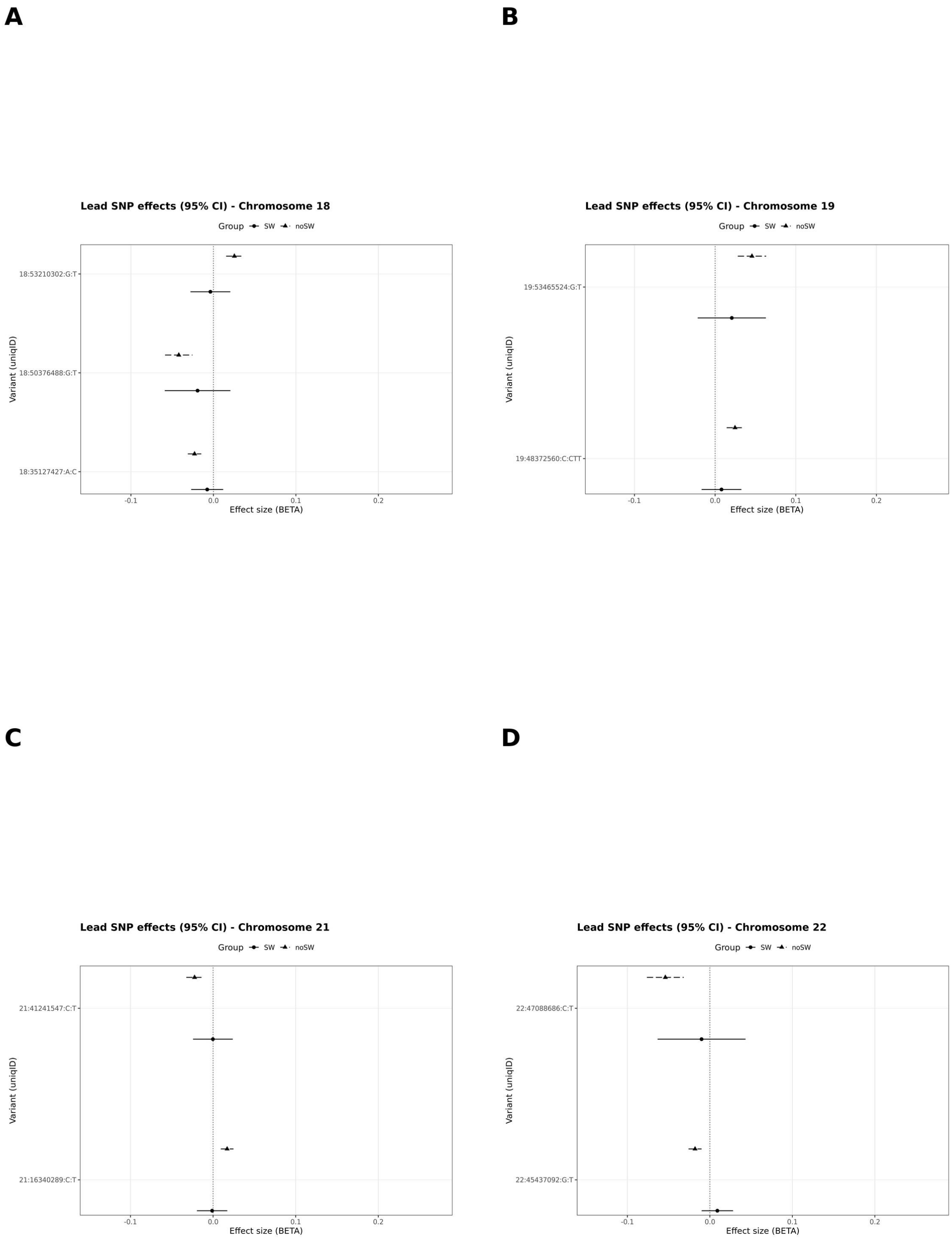

Supplementary Figure 7. Lead SNP effects by shift work status for chromosomes 18, 19, 21, and 22. Forest plots show lead SNP effect estimates and 95% confidence intervals for chromosomes 18, 19, 21, and 22. Panels show: A, chromosome 18; B, chromosome 19; C, chromosome 21; and D, chromosome 22. Points represent beta effect estimates and horizontal lines represent 95% confidence intervals. Circles with solid lines indicate the shift work (SW) group, and triangles with dashed lines indicate the non-shift work (noSW) group. The vertical dotted line indicates a null effect estimate of zero.

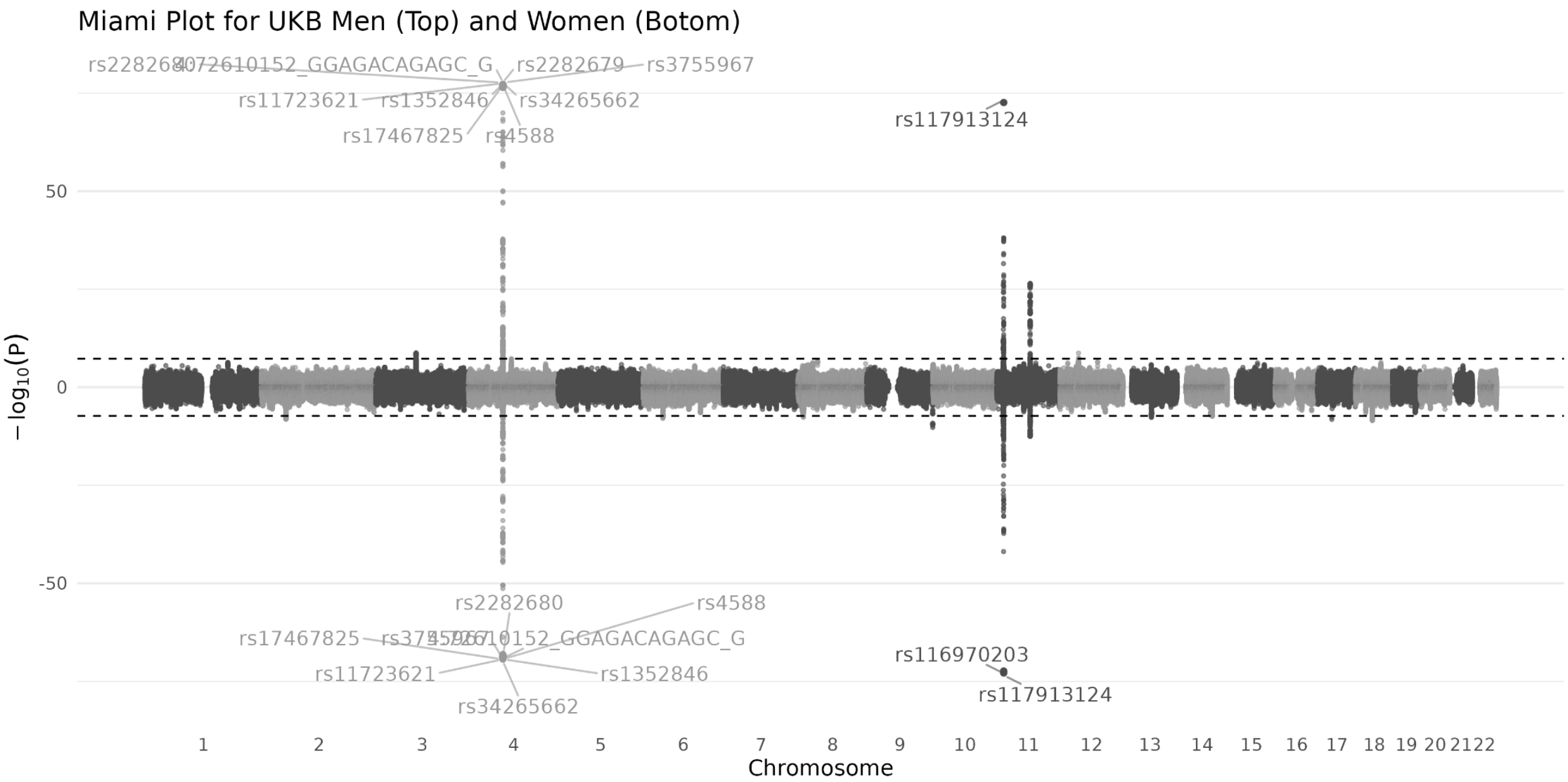

Supplementary Figure 8: Miami plot of CII-GWASs results for European ancestry UKB participants: men (N=148,473) versus women (N= 164,462; bottom).

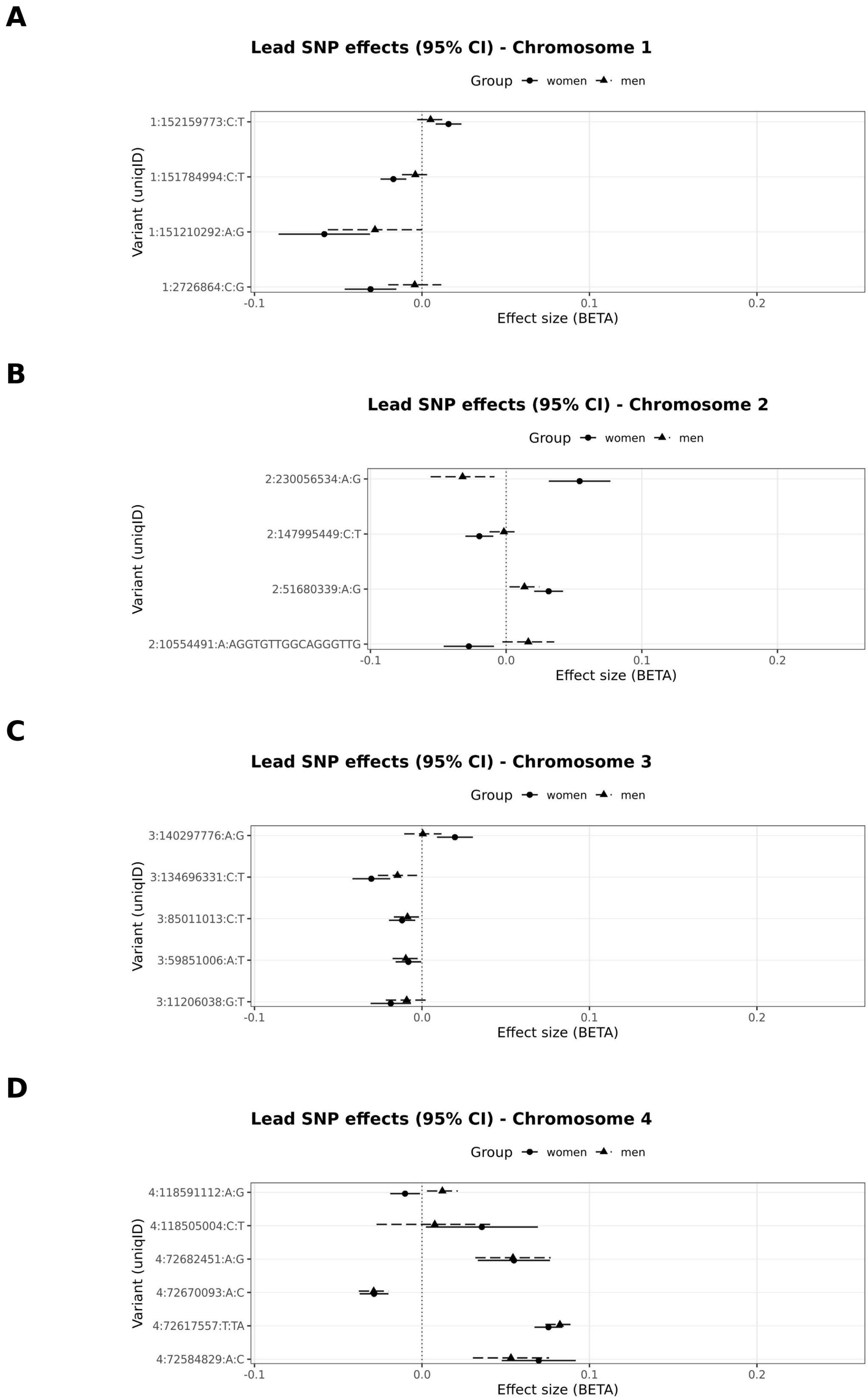

Supplementary Figure 9. Sex-specific GWAS effect sizes (95% CI) for lead SNPs identified in SNP x sex interaction analysis of the CII. Panels show A, chromosome 1; B, chromosome 2; C, chromosome 3; and D, chromosome 4. Points represent beta effect estimates, and horizontal lines represent 95% confidence intervals. Circles with solid lines indicate women, and triangles with dashed lines indicate men. The vertical dotted line indicates a null effect estimate of zero.

A

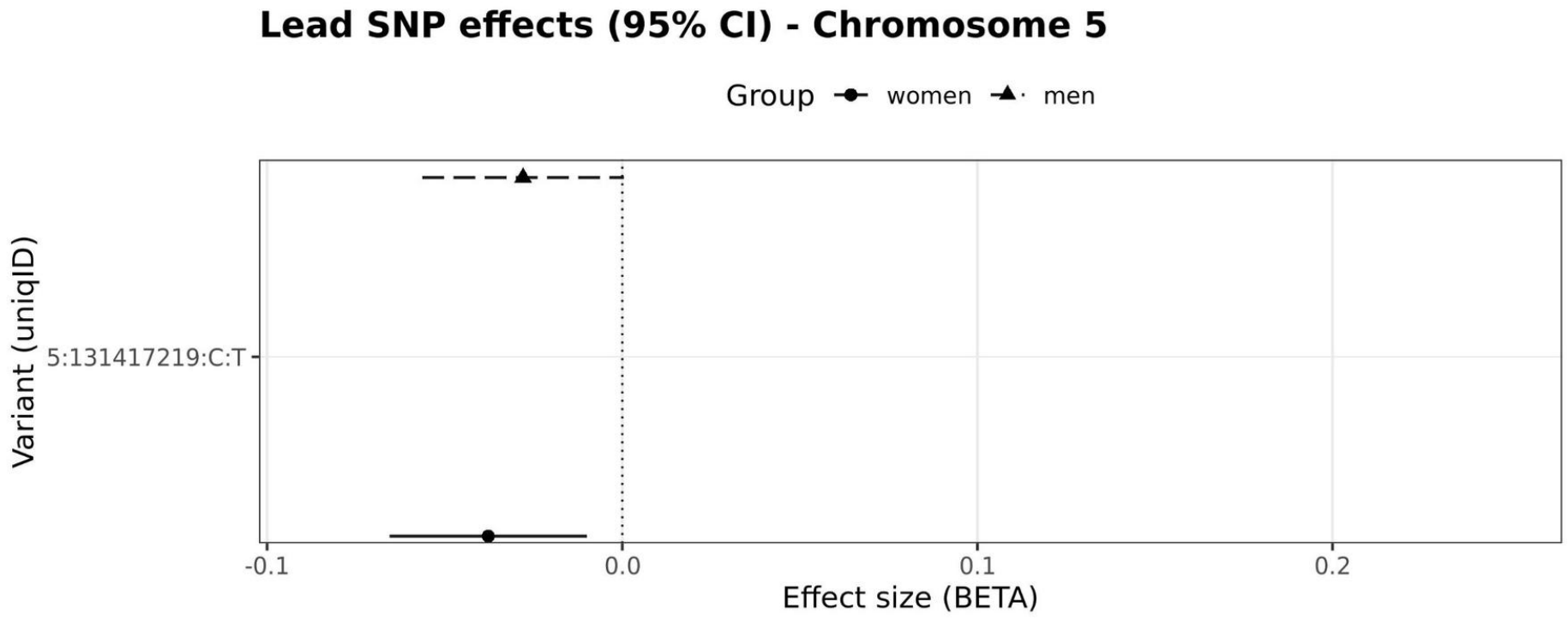

B

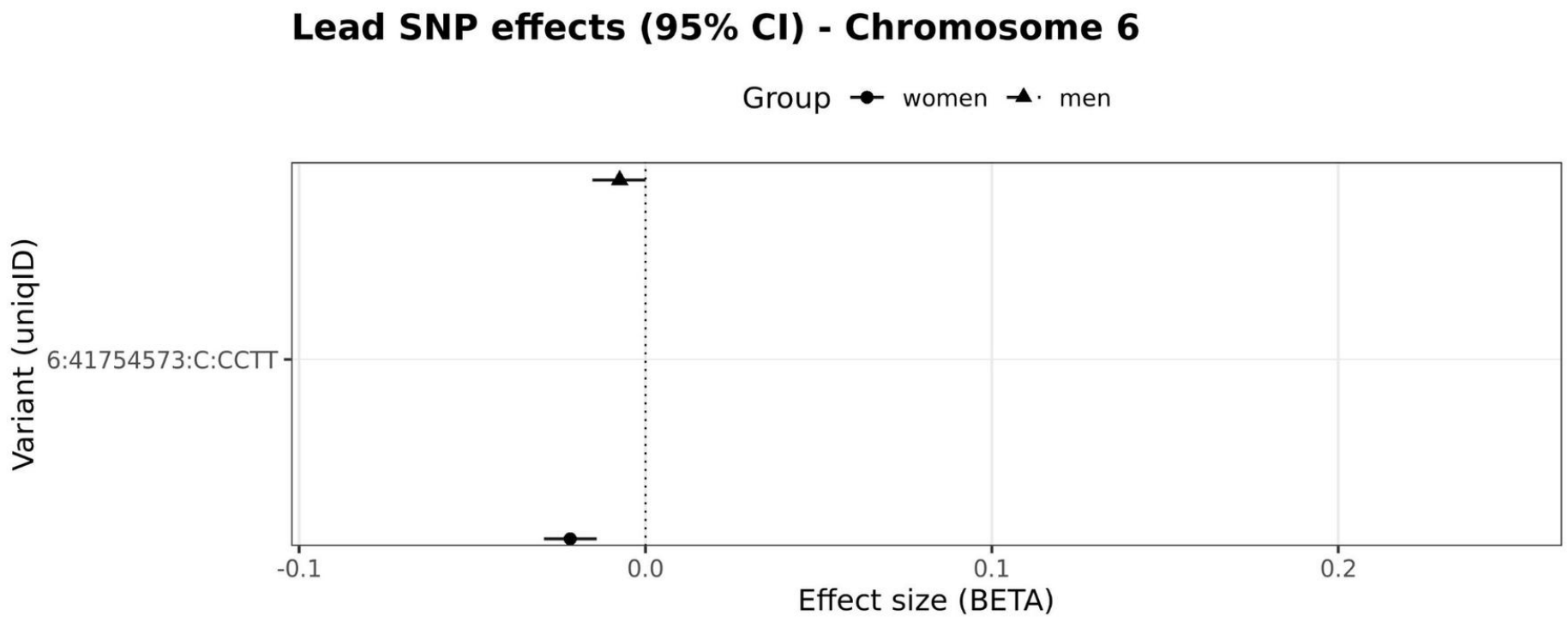

C

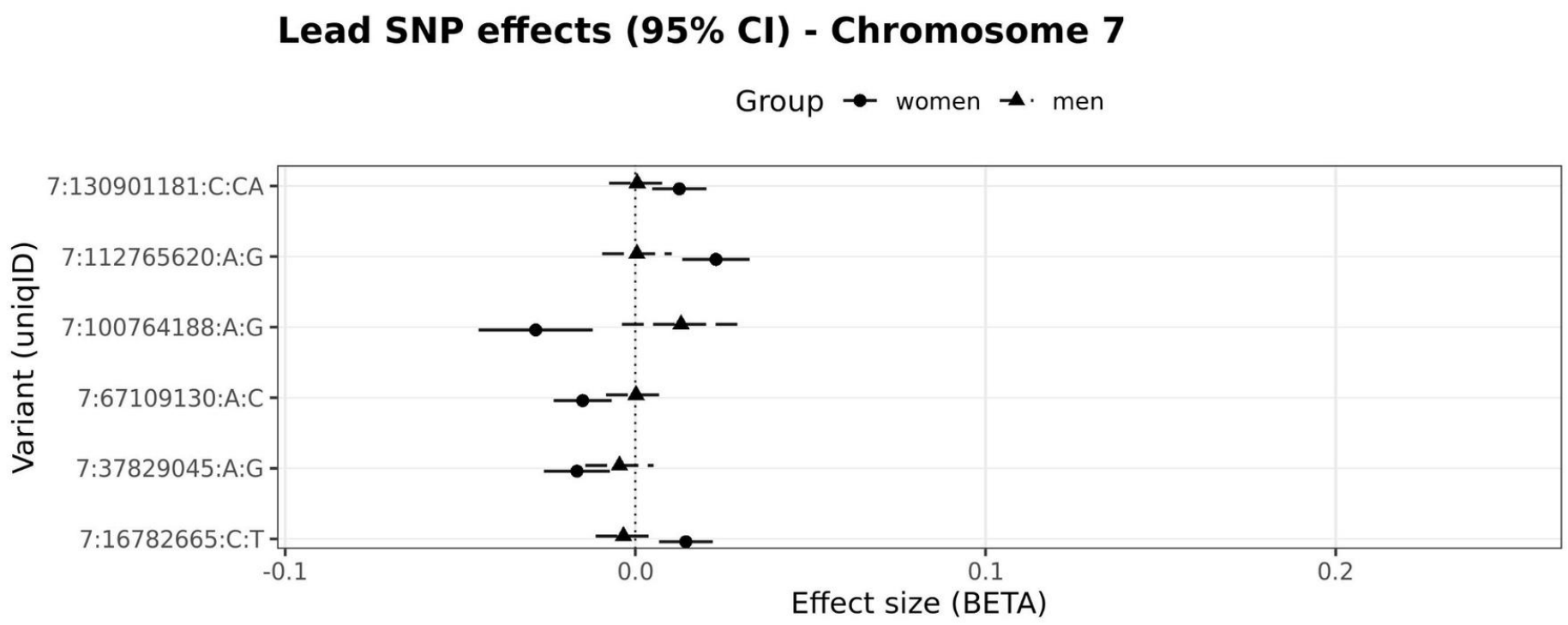

D

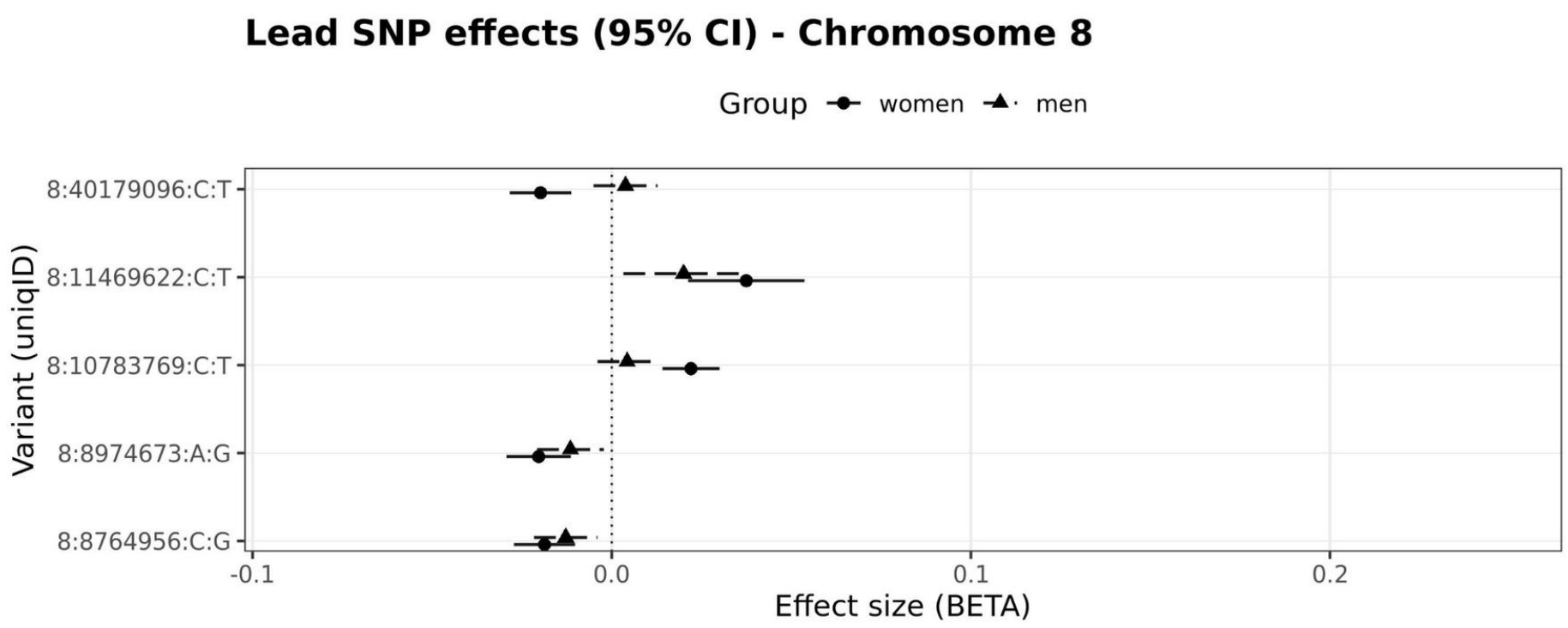

Supplementary Figure 10. Sex-specific GWAS effect sizes (95% CI) for lead SNPs identified in SNP x sex interaction analysis of the CII. Panels show A, chromosome 5; B, chromosome 6; C, chromosome 7; and D, chromosome 8. Points represent beta effect estimates, and horizontal lines represent 95% confidence intervals. Circles with solid lines indicate women, and triangles with dashed lines indicate men. The vertical dotted line indicates a null effect estimate of zero.

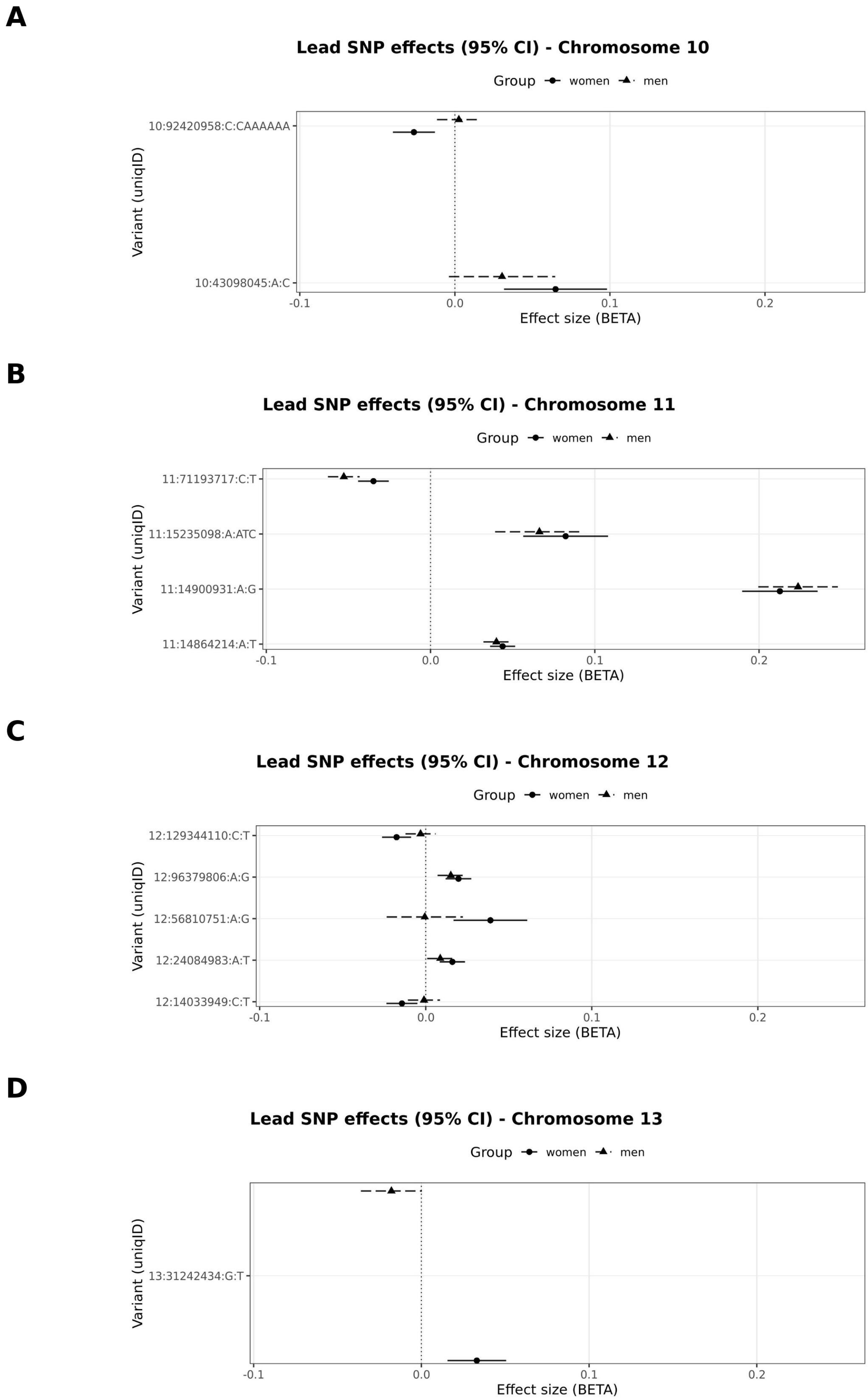

Supplementary Figure 11. Sex-specific GWAS effect sizes (95% CI) for lead SNPs identified in SNP x sex interaction analysis of the CII. Panels show A, chromosome 10; B, chromosome 11; C, chromosome 12; and D, chromosome 13. Points represent beta effect estimates, and horizontal lines represent 95% confidence intervals. Circles with solid lines indicate women, and triangles with dashed lines indicate men. The vertical dotted line indicates a null effect estimate of zero.

A

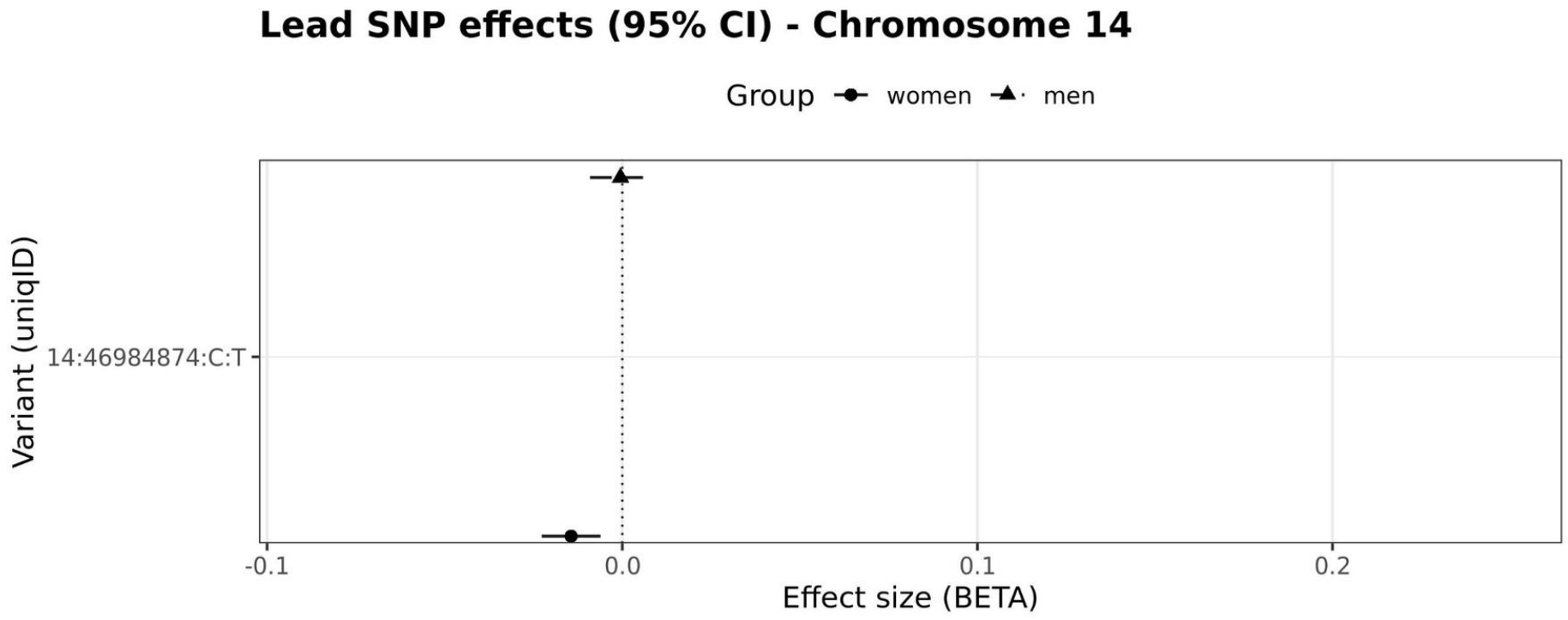

B

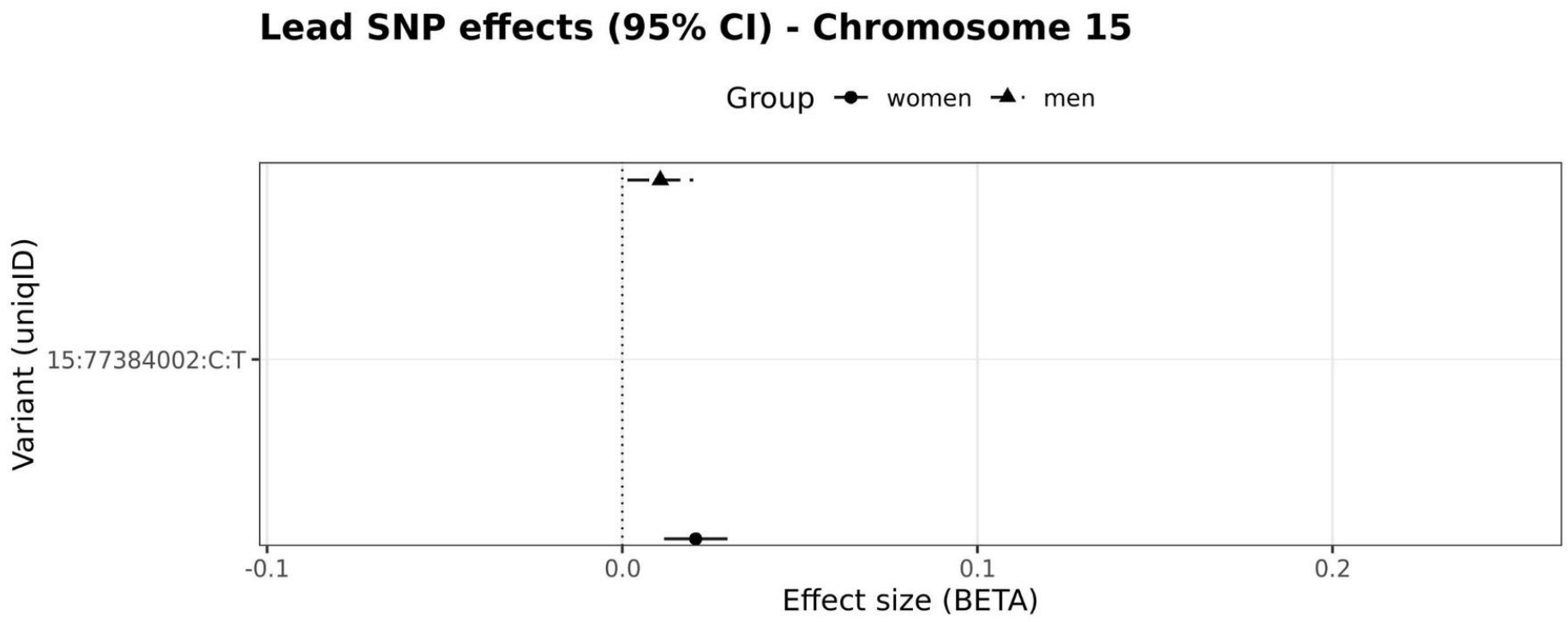

C

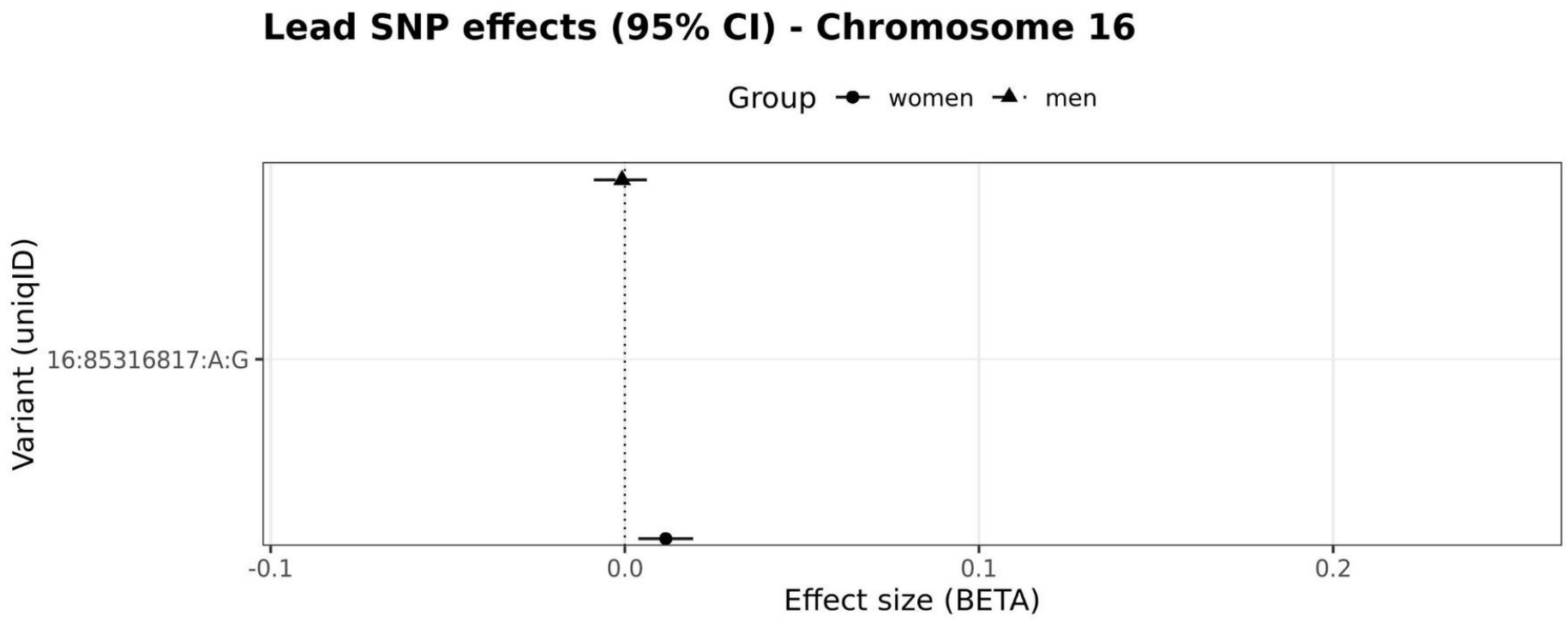

D

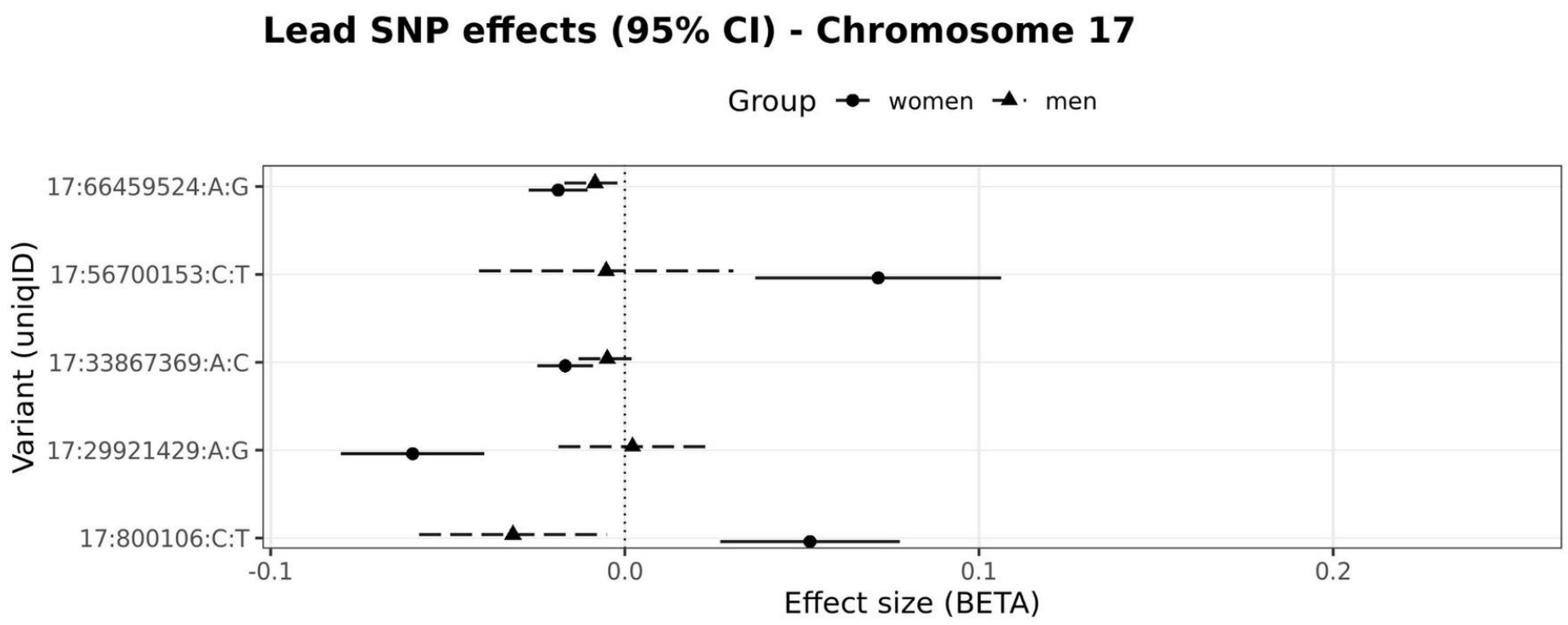

Supplementary Figure 12. Sex-specific GWAS effect sizes (95% CI) for lead SNPs identified in SNP x sex interaction analysis of the CII. Panels show A, chromosome 14; B, chromosome 15; C, chromosome 16; and D, chromosome 17. Points represent beta effect estimates, and horizontal lines represent 95% confidence intervals. Circles with solid lines indicate women, and triangles with dashed lines indicate men. The vertical dotted line indicates a null effect estimate of zero.

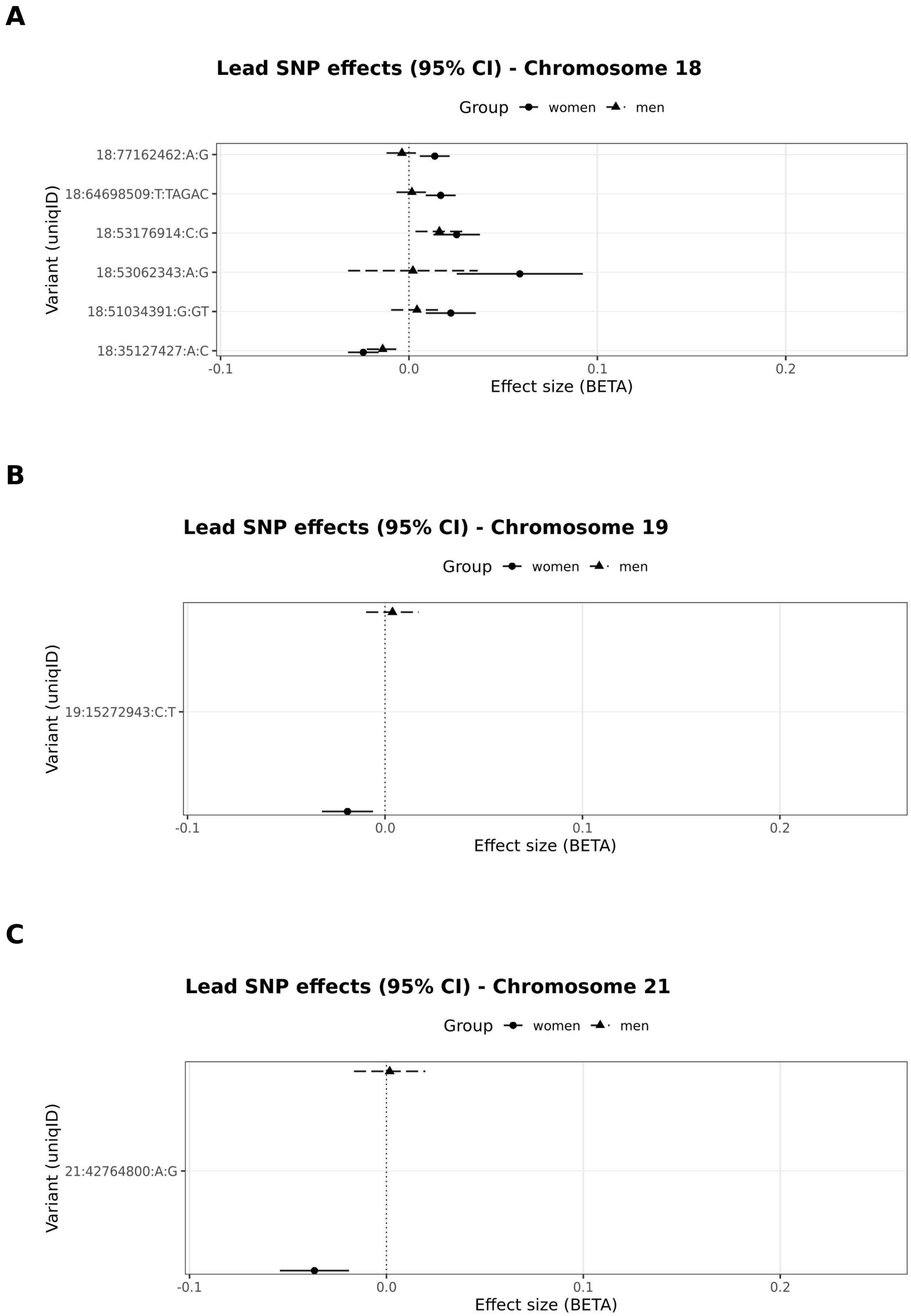

Supplementary Figure 13. Sex-specific GWAS effect sizes (95% CI) for lead SNPs identified in SNP x sex interaction analysis of the CII. Panels show A, chromosome 18; B, chromosome 19; and C, chromosome 21. Points represent beta effect estimates, and horizontal lines represent 95% confidence intervals. Circles with solid lines indicate women, and triangles with dashed lines indicate men. The vertical dotted line indicates a null effect estimate of zero.

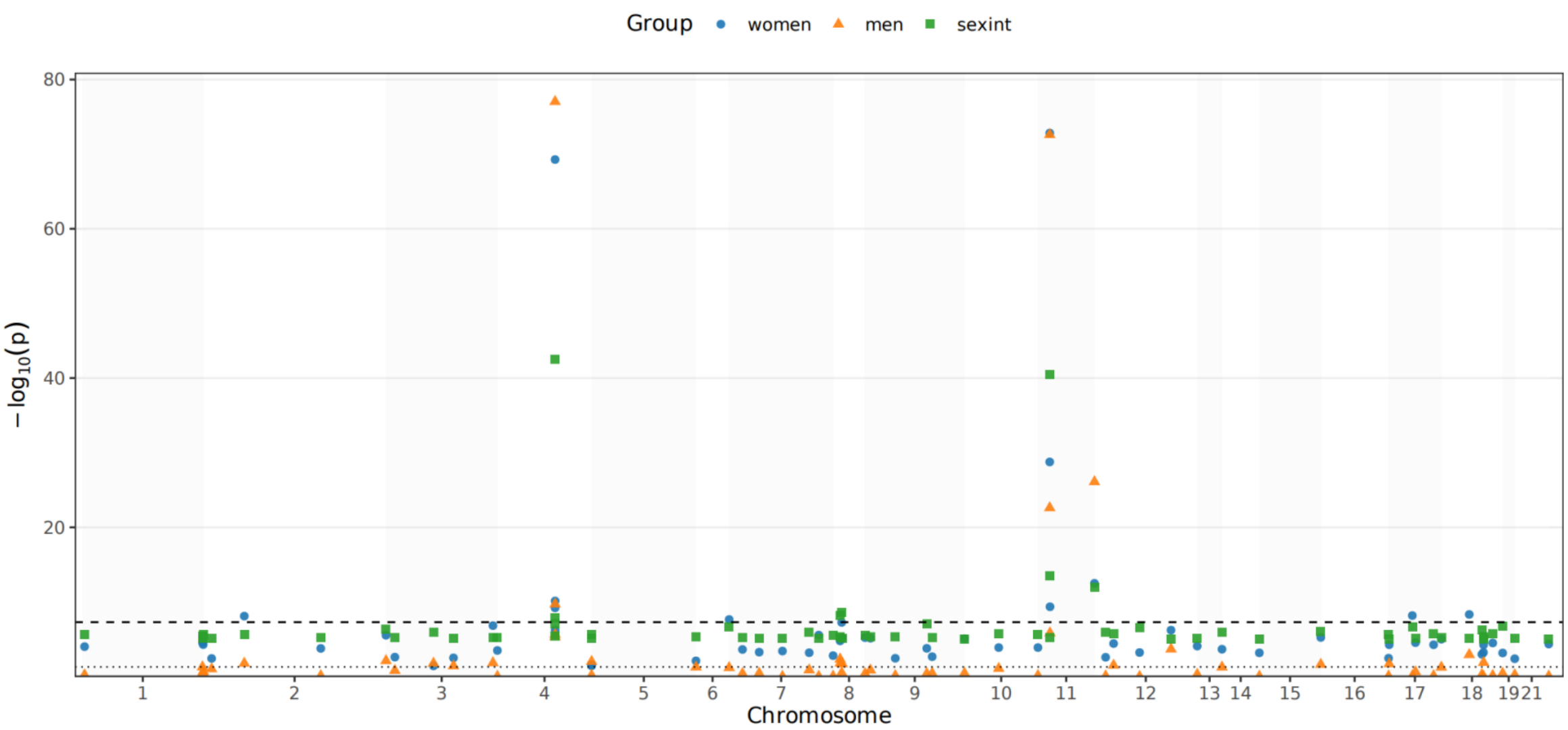

Supplementary Figure 14: Manhattan plot of –log10(p) values for lead SNPs from the SNP × sex (sexint) and corresponding results in UKB European ancestry men (N=148,473) and women (N= 164,462).

A

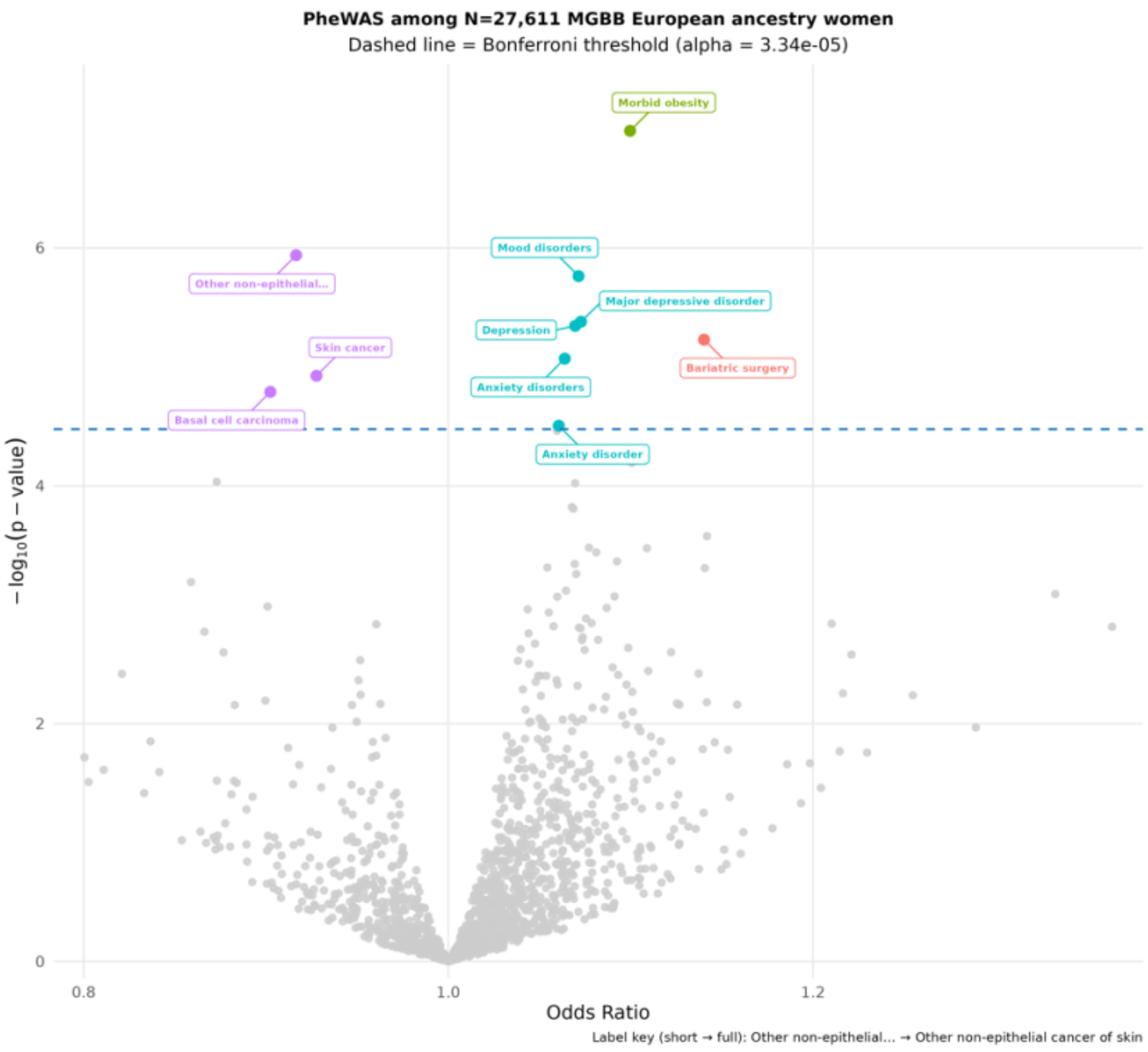

B

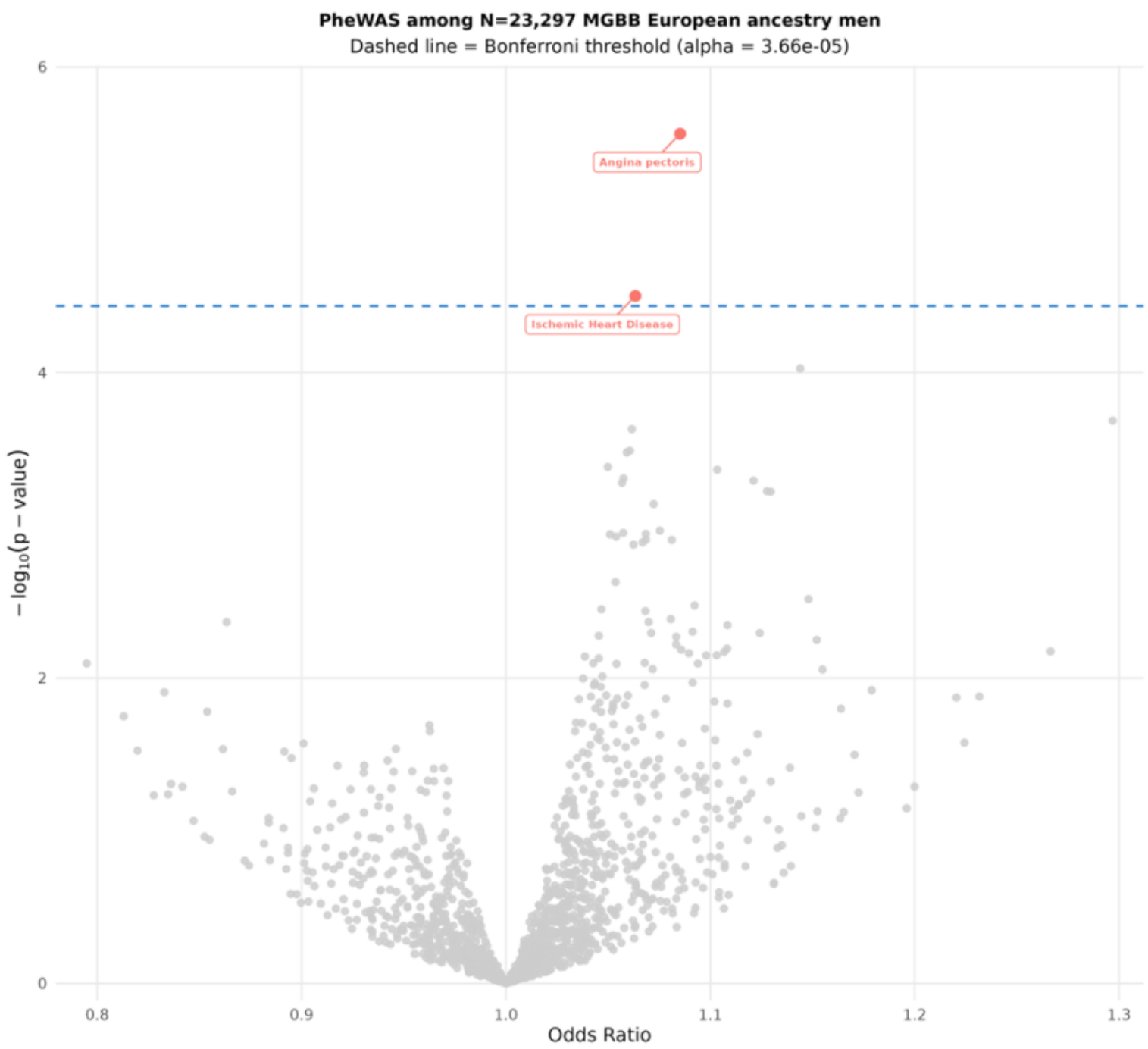

Supplementary Figure 15: Results of the PheWAS analysis of the PRS-CS polygenic risk score for the CII (CII-PRS) among European ancestry MGB Biobank participants: (A) women, N=27,611; (B) men, N=23,297.

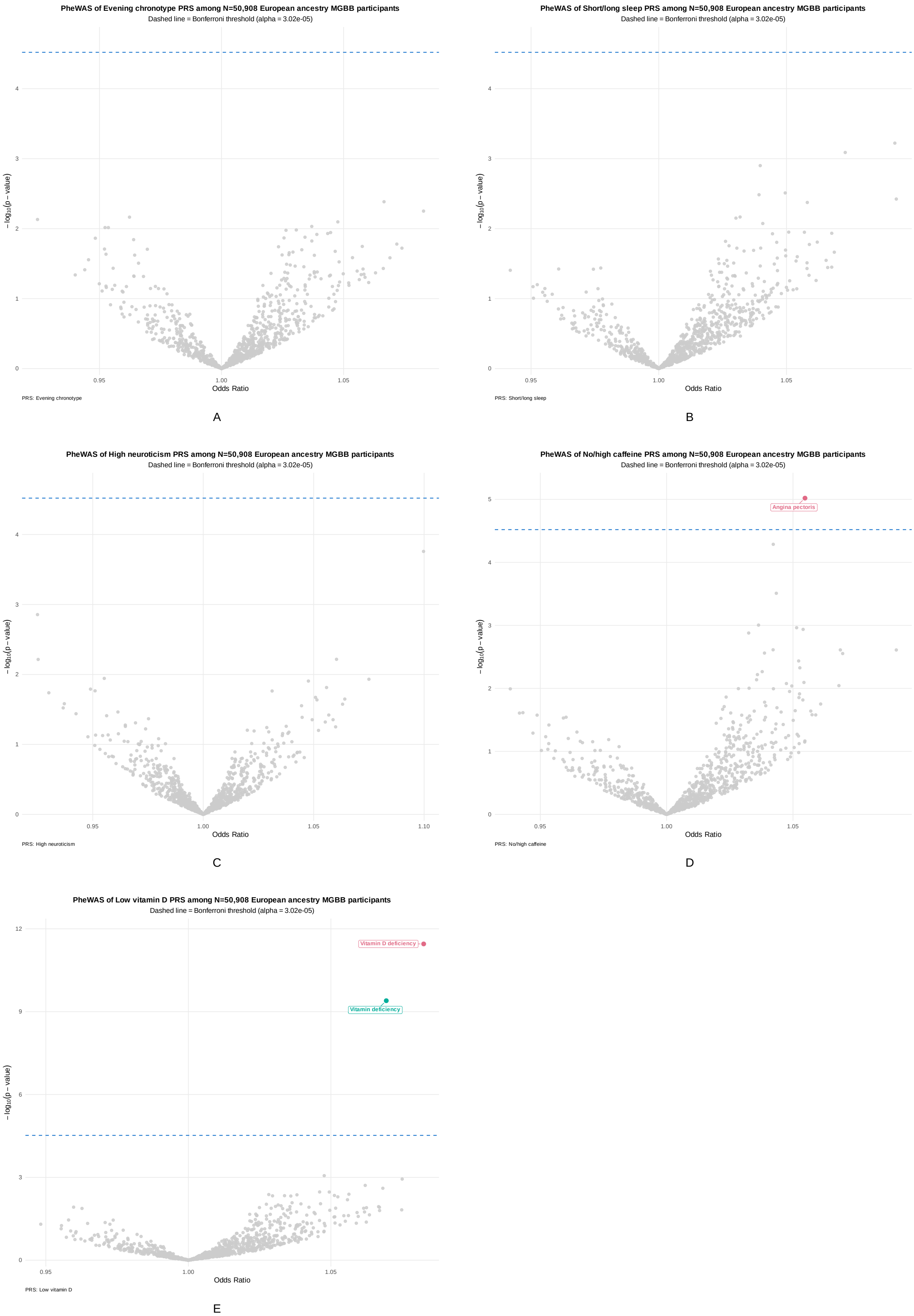

Supplementary Figure 16. PheWAS sensitivity analyses of component-weighted CII PRSs in MGBB. PheWAS results for component-weighted PRSs among 50,908 European ancestry MGBB participants. Panels show A, evening chronotype-weighted PRS; B, short/long sleep duration-weighted PRS; C, high neuroticism-weighted PRS; D, no/high caffeine intake-weighted PRS; and E, low vitamin D-weighted PRS. The x-axis shows odds ratios, and the y-axis shows –log10(p-value). The dashed horizontal line indicates the Bonferroni significance threshold, alpha = 3.02×10^{-5}.

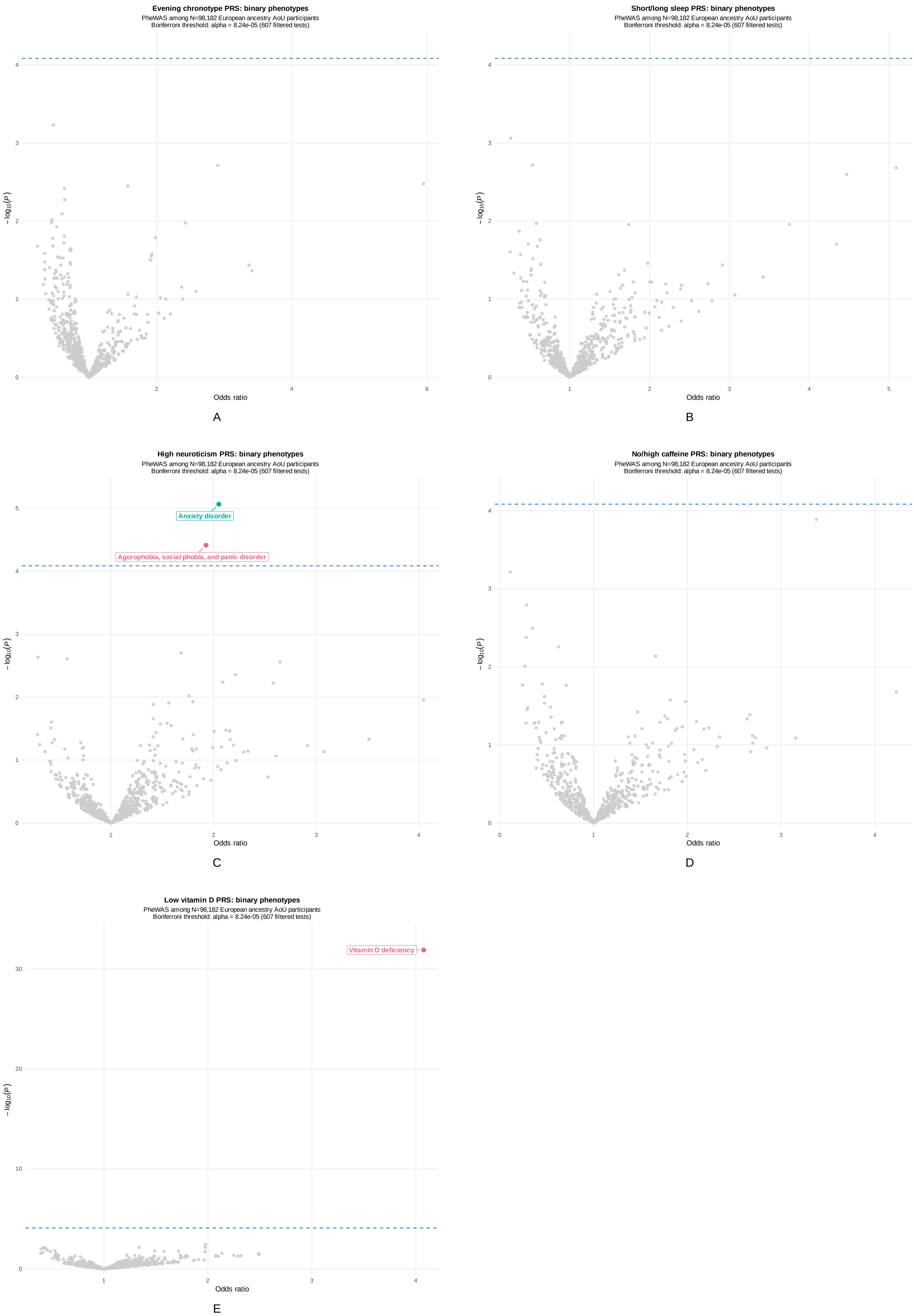

Supplementary Figure 17. PheWAS sensitivity analyses of component-weighted CII PRSs in AoU. PheWAS results for component-weighted PRSs among 98,182 European ancestry AoU participants. Panels show A, evening chronotype-weighted PRS; B, short/long sleep duration-weighted PRS; C, high neuroticism-weighted PRS; D, no/high caffeine intake-weighted PRS; and E, low vitamin D-weighted PRS. The x-axis shows odds ratios, and the y-axis shows –log10(P). The Bonferroni significance threshold was alpha = 8.24×10^{-5} across 607 filtered tests.

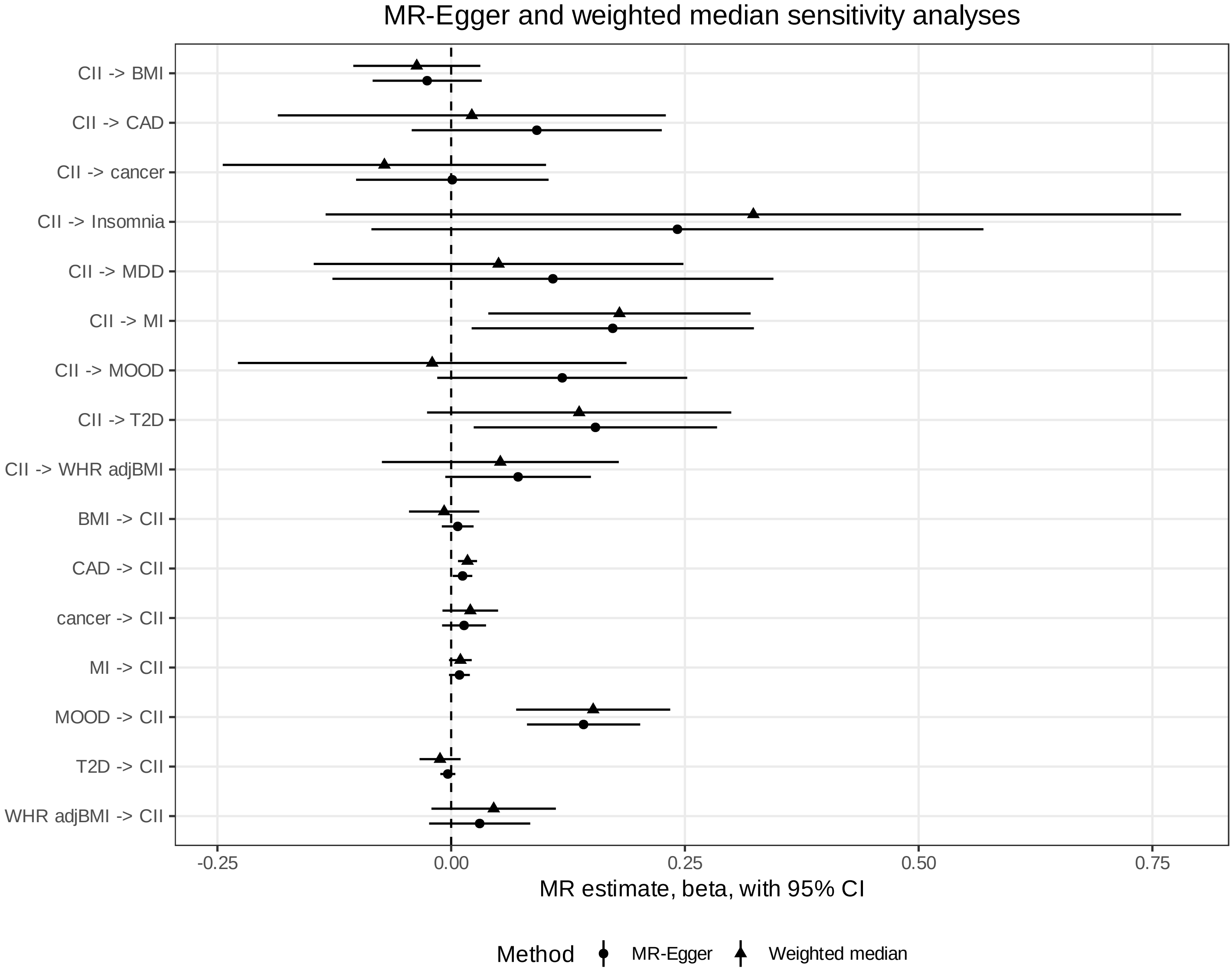

Supplementary Figure 18. MR-Egger and weighted median sensitivity analyses for bidirectional MR associations between CII and cardiometabolic, psychiatric, sleep, and cancer-related traits. Estimates are shown for forward associations of CII with BMI, coronary artery disease (CAD), cancer, insomnia, major depressive disorder (MDD), myocardial infarction (MI), mood disorder, type 2 diabetes (T2D), and waist-to-hip ratio adjusted for BMI (WHRadjBMI), as well as reverse associations of these traits with CII. Points indicate MR effect estimates, and horizontal lines indicate 95% confidence intervals. The vertical dashed line indicates the null effect estimate.

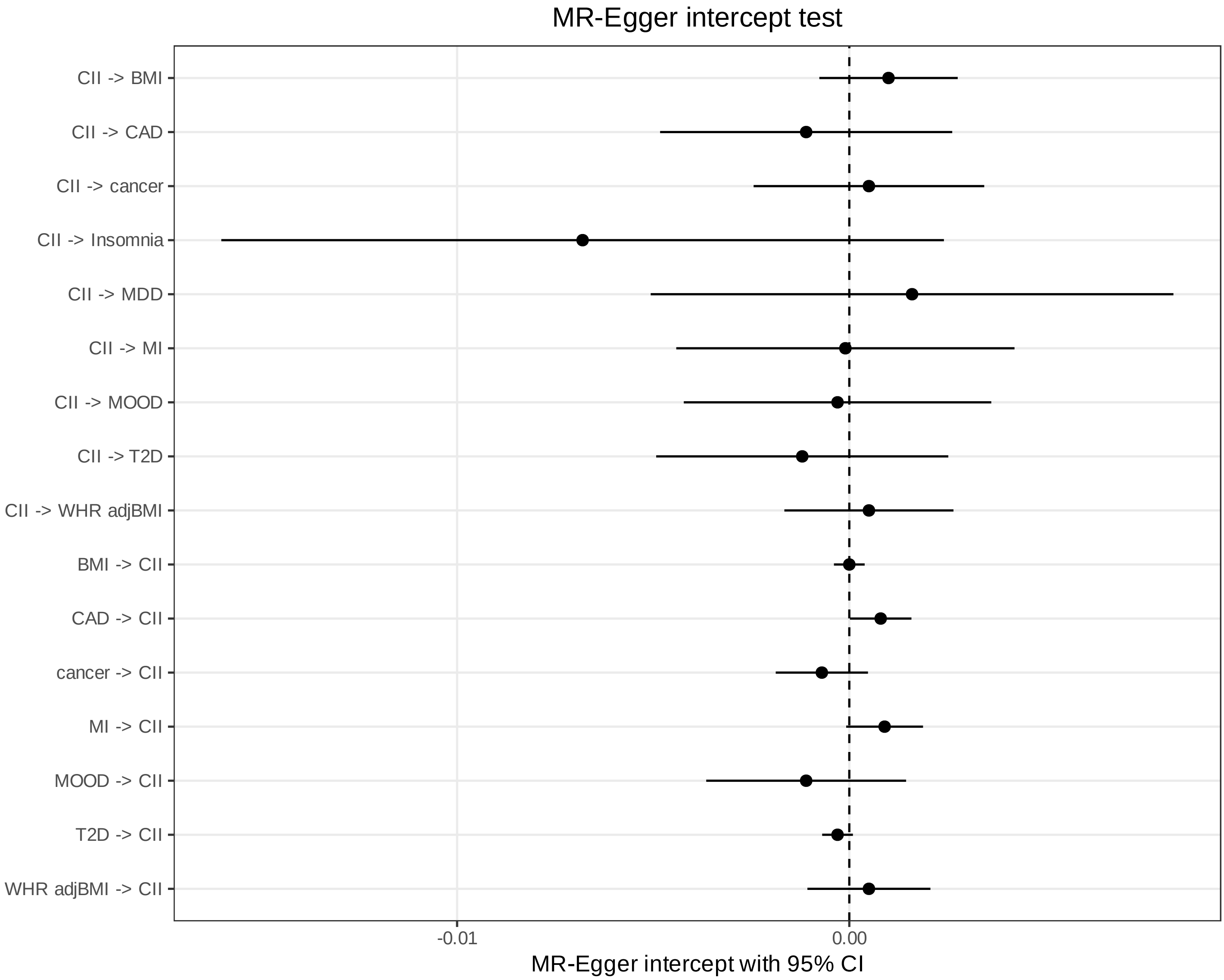

Supplementary Figure 19. MR-Egger intercept tests for bidirectional MR associations between CII and cardiometabolic, psychiatric, sleep, and cancer-related traits. Intercepts are shown for forward associations of CII with each outcome and reverse associations of each trait with CII. Points indicate MR-Egger intercept estimates and horizontal lines indicate 95% confidence intervals. The vertical dashed line indicates an intercept of zero.

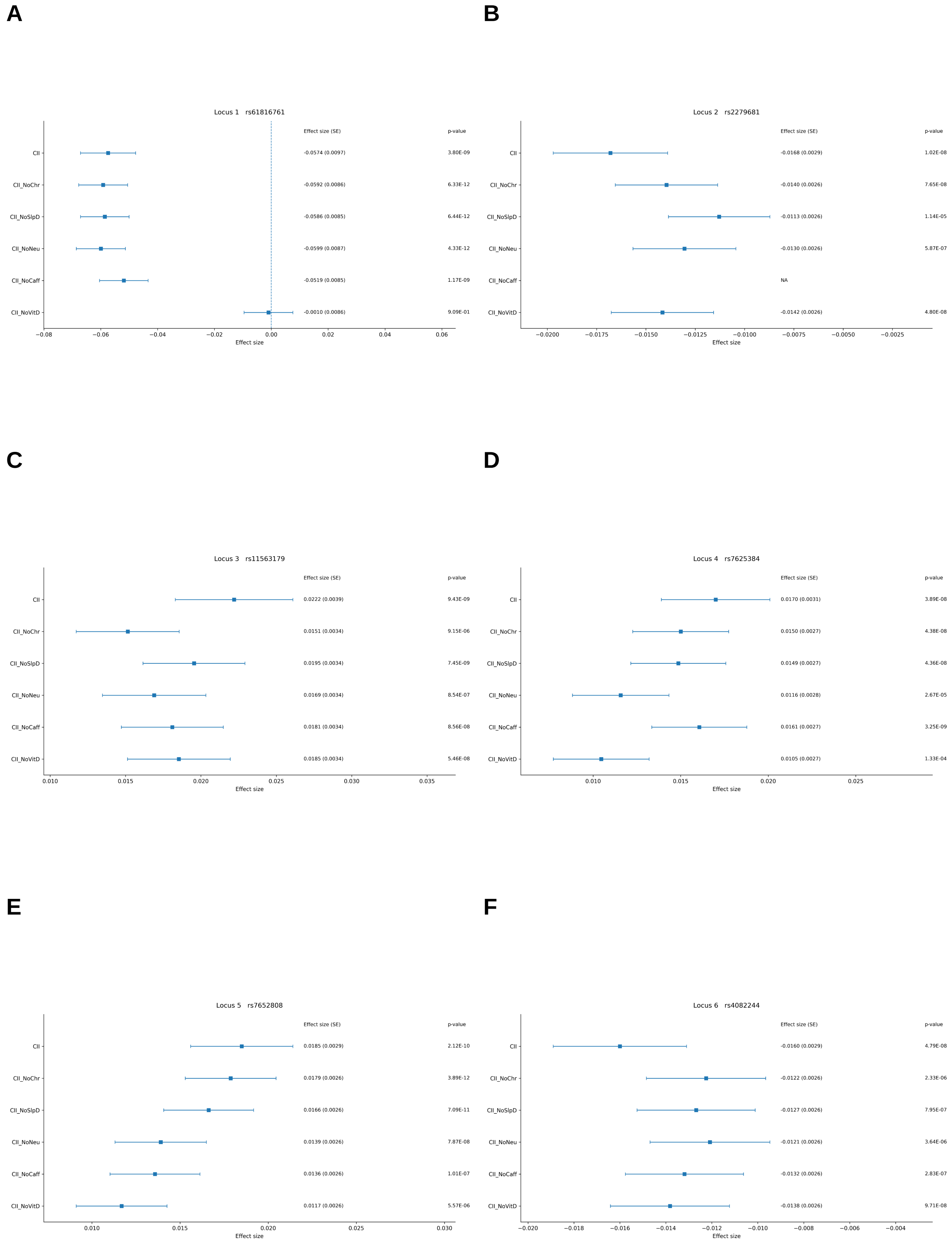

Supplementary Figure 20. Forest plots of lead CII loci across the main CII GWAS and leave-one-component-out CII GWAS analyses. Panels show A, locus 1, rs61816761; B, locus 2, rs2279681; C, locus 3, rs11563179; D, locus 4, rs7625384; E, locus 5, rs7652808; and F, locus 6, rs4082244. Each subplot shows the effect estimate and 95% confidence interval for a lead variant identified in the main CII GWAS, compared across the original CII and the five leave-one-component-out versions of the index: CII excluding evening chronotype (CII_NoChr); CII excluding short or long sleep duration (CII_NoSlpD); CII excluding high neuroticism (CII_NoNeu); CII excluding no or high caffeinated coffee intake (CII_NoCaff); and CII excluding low vitamin D (CII_NoVitD). Each leave-one-component-out index was based on the remaining four CII components.

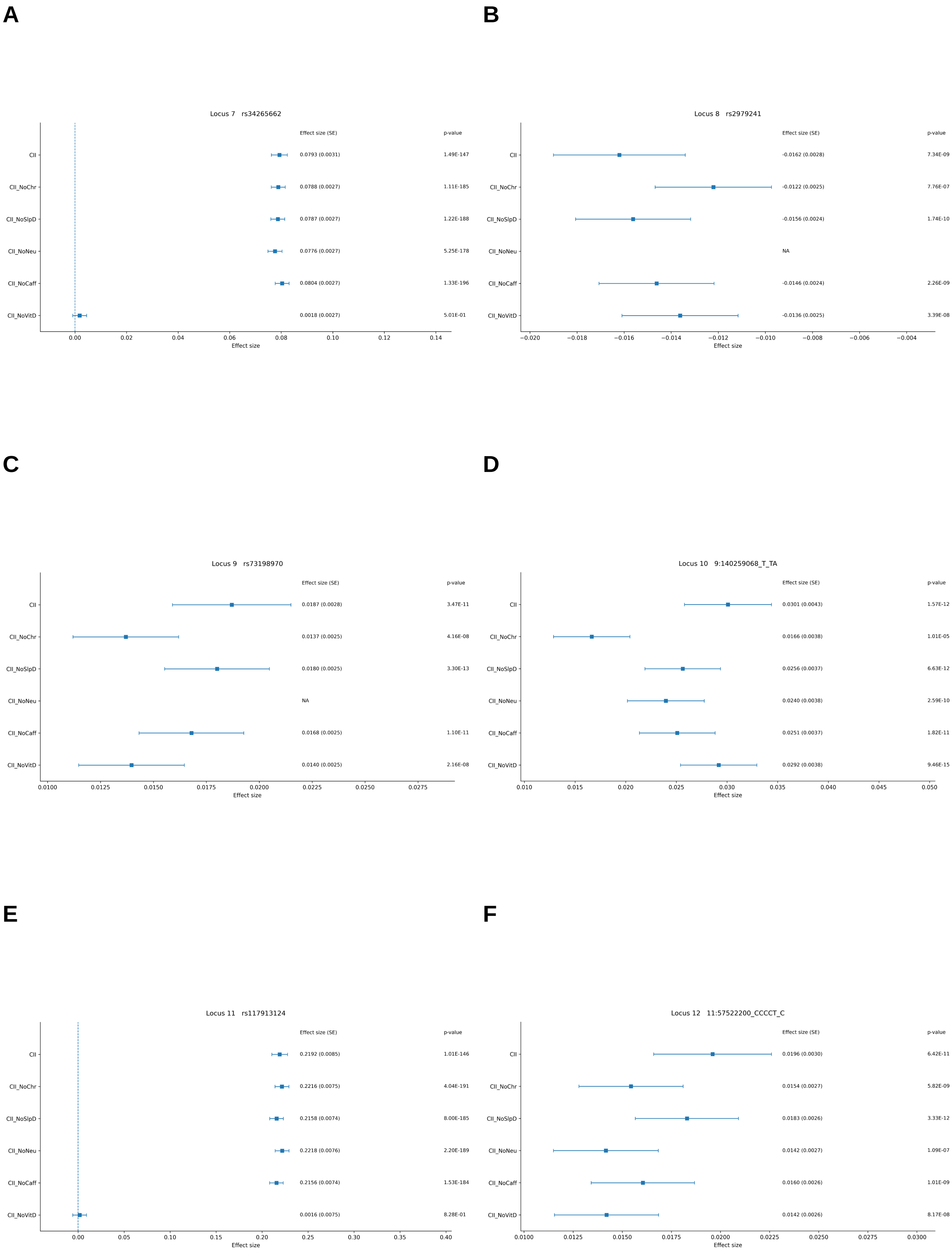

Supplementary Figure 21. Forest plots of lead CII loci across the main CII GWAS and leave-one-component-out CII GWAS analyses. Panels show A, locus 7, rs34265662; B, locus 8, rs2979241; C, locus 9, rs73198970; D, locus 10, 9:140259068_T_TA; E, locus 11, rs117913124; and F, locus 12, 11:57522200_CCCCT_C. Each subplot shows the effect estimate and 95% confidence interval for a lead variant identified in the main CII GWAS, compared across the original CII and the five leave-one-component-out versions of the index: CII_NoChr, CII_NoSlpD, CII_NoNeu, CII_NoCaff, and CII_NoVitD. Each leave-one-component-out index was based on the remaining four CII components.

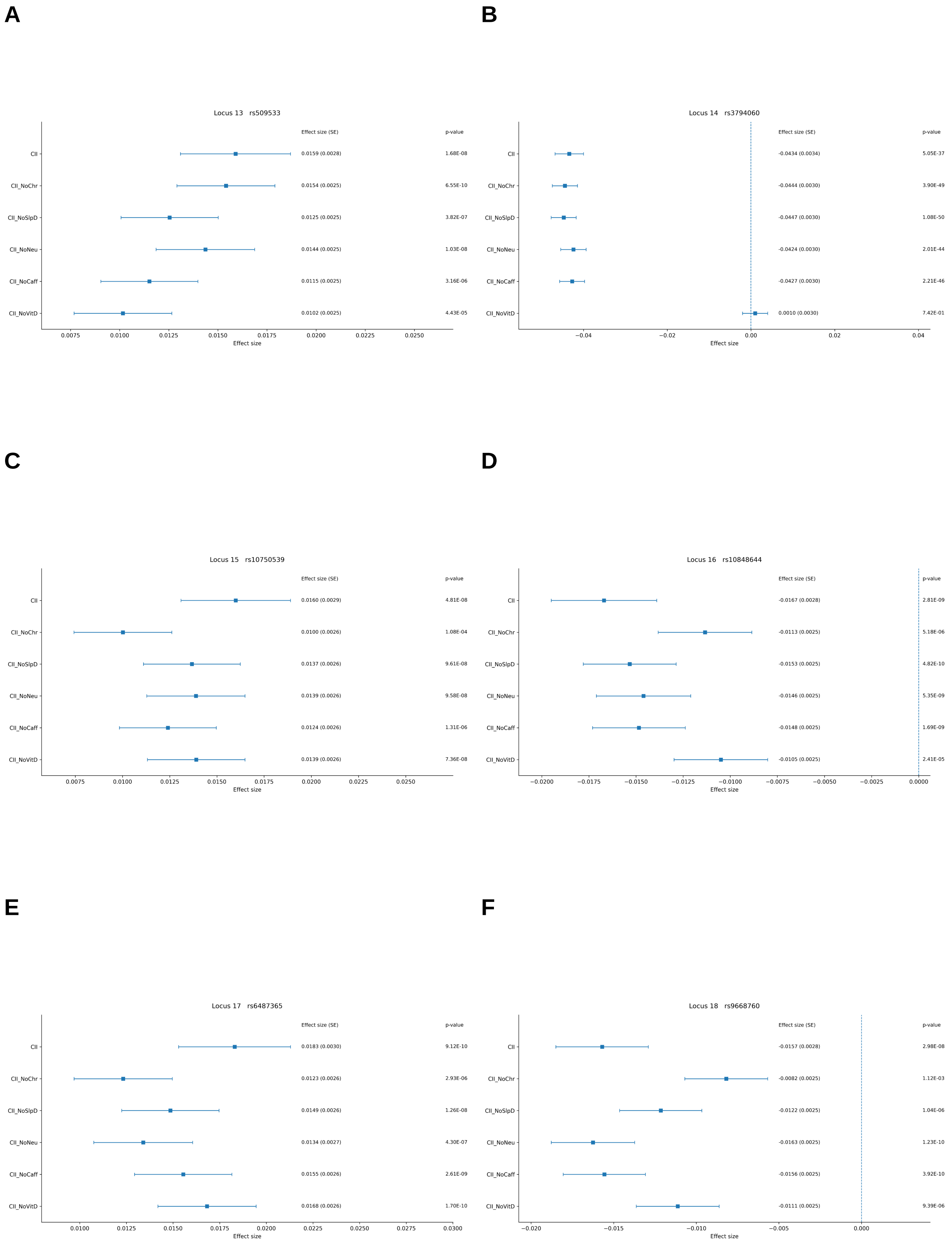

Supplementary Figure 22. Forest plots of lead CII loci across the main CII GWAS and leave-one-component-out CII GWAS analyses. Panels show A, locus 13, rs509533; B, locus 14, rs3794060; C, locus 15, rs10750539; D, locus 16, rs10848644; E, locus 17, rs6487365; and F, locus 18, rs9668760. Each subplot shows the effect estimate and 95% confidence interval for a lead variant identified in the main CII GWAS, compared across the original CII and the five leave-one-component-out versions of the index: CII_NoChr, CII_NoSlpD, CII_NoNeu, CII_NoCaff, and CII_NoVitD. Each leave-one-component-out index was based on the remaining four CII components.

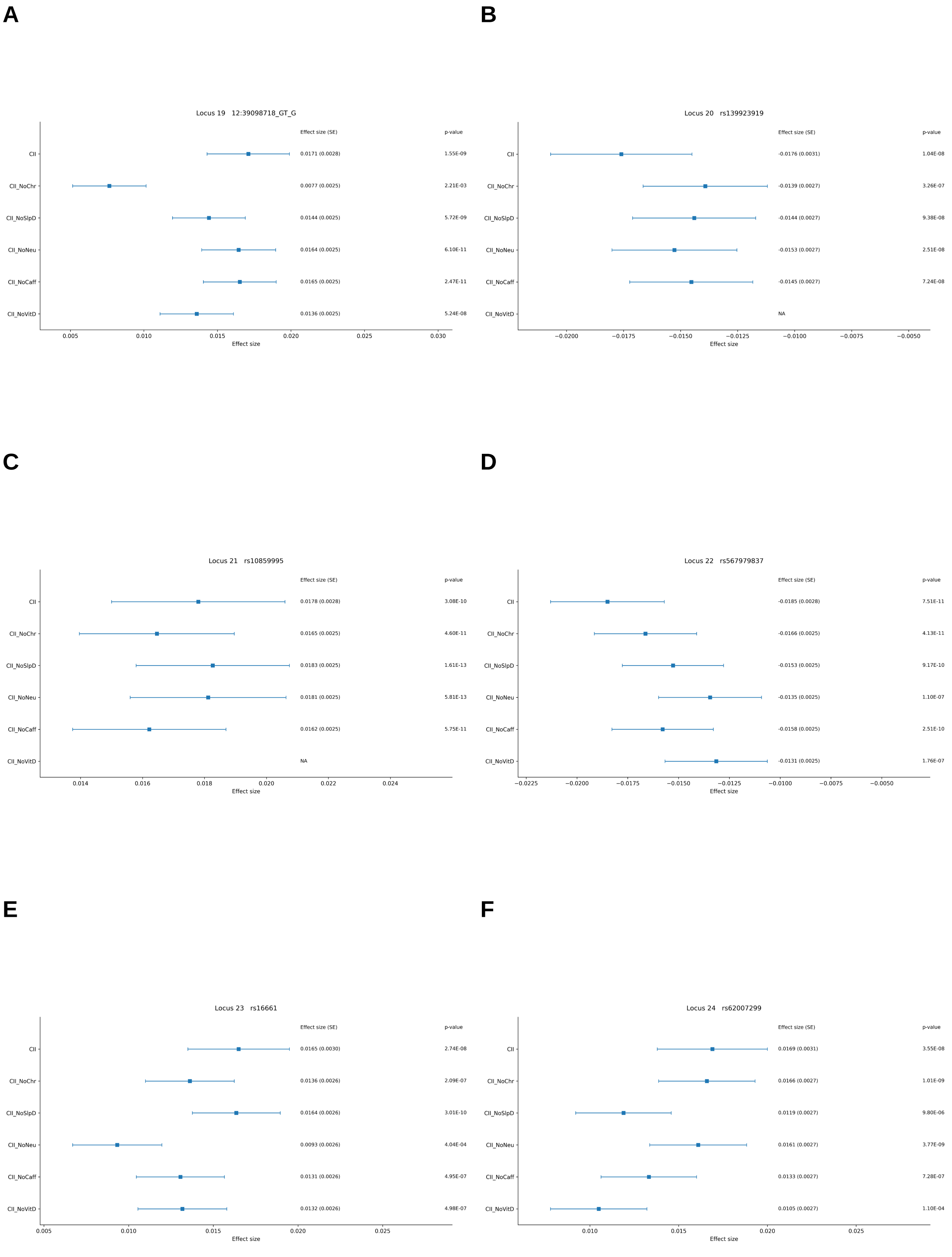

Supplementary Figure 23. Forest plots of lead CII loci across the main CII GWAS and leave-one-component-out CII GWAS analyses. Panels show A, locus 19, 12:39098718_GT_G; B, locus 20, rs139923919; C, locus 21, rs10859995; D, locus 22, rs567979837; E, locus 23, rs16661; and F, locus 24, rs62007299. Each subplot shows the effect estimate and 95% confidence interval for a lead variant identified in the main CII GWAS, compared across the original CII and the five leave-one-component-out versions of the index: CII_NoChr, CII_NoSlpD, CII_NoNeu, CII_NoCaff, and CII_NoVitD. Each leave-one-component-out index was based on the remaining four CII components.

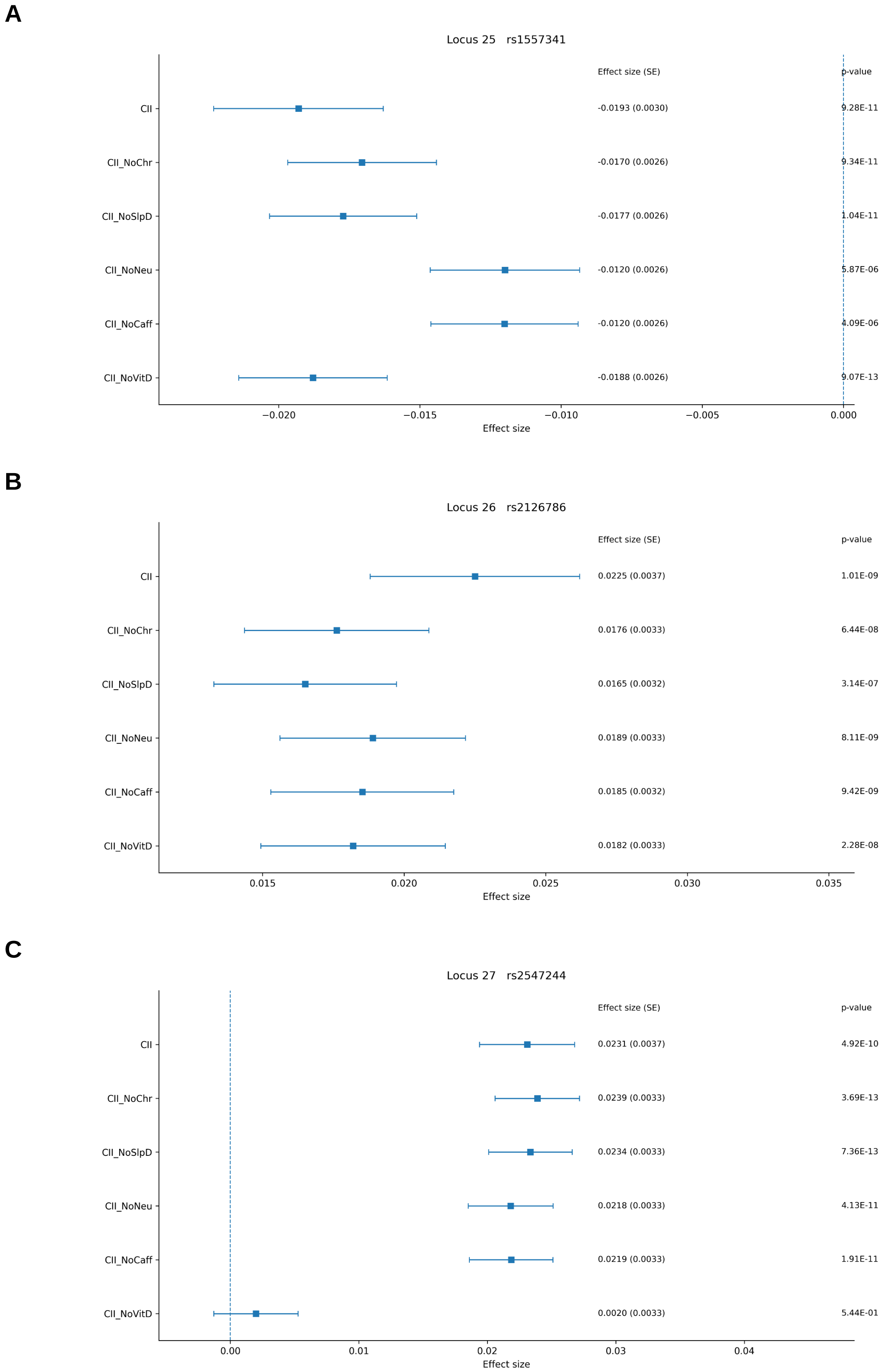

Supplementary Figure 24. Forest plots of lead CII loci across the main CII GWAS and leave-one-component-out CII GWAS analyses. Panels show A, locus 25, rs1557341; B, locus 26, rs2126786; and C, locus 27, rs2547244. Each subplot shows the effect estimate and 95% confidence interval for a lead variant identified in the main CII GWAS, compared across the original CII and the five leave-one-component-out versions of the index: CII_NoChr, CII_NoSlpD, CII_NoNeu, CII_NoCaff, and CII_NoVitD. Each leave-one-component-out index was based on the remaining four CII components.

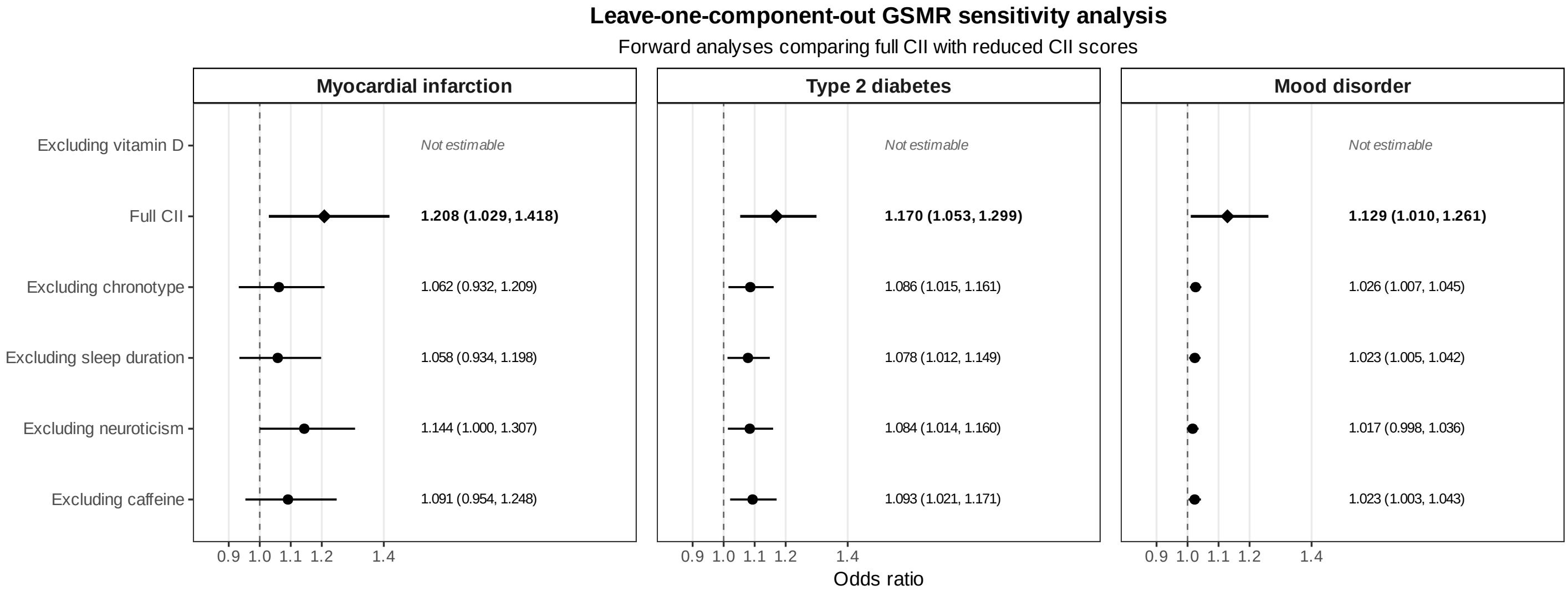

Supplementary Figure 25. Leave-one-component-out MR sensitivity analyses for key forward CII associations with myocardial infarction, type 2 diabetes, and mood swings. Panels show forward MR estimates for the association of CII with A, myocardial infarction; B, type 2 diabetes; and C, mood swings. Each panel compares the full CII score with reduced CII scores generated by excluding one component at a time. Points indicate odds ratios and horizontal lines indicate 95% confidence intervals. The vertical dashed line indicates an odds ratio of 1.
