# Supplementary material for: Genetic architecture of a Circadian Imbalance Index: genome-wide association, phenome-wide association, and Mendelian randomisation analyses
Source: eBioMedicine. 2026 Jul 21;130:106380. doi: 10.1016/j.ebiom.2026.106380 (PMC13476902; doi:10.1016/j.ebiom.2026.106380)
Supplement: Supplementary file 2 [file mmc2.docx]

**Supplement 3 : Methods for** **the manuscript “Genetic architecture of a Circadian Imbalance Index: genome-wide association, phenome-wide association, and Mendelian randomization analyses”**

Contents

[Genetic data quality control in the UKB	1](#_Toc215595863)

[Functional mapping and pathway analysis	2](#_Toc215595864)

[Polygenic Risk score analysis in the UKB	2](#_Toc215595865)

[Marginal and conditional two sample Mendelian Randomization with mtCOJO	2](#_Toc215595866)

[PheWAS of the CII-PRS in the MGB biobank	3](#_Toc215595867)

[PheWAS of the CII-PRS in the All of US	4](#_Toc215595868)

[Replication analysis in the Nurses’ Health Study II	5](#_Toc215595869)

[Phenotypes’ assessment	5](#_Toc215595870)

[Replication of phenotypic associations with CKM	7](#_Toc215595871)

[Replication of the CII GWAS results in the NHS2	8](#_Toc215595872)

[References	9](#_Toc215595873)

# Genetic data quality control in the UKB

For quality control in the UKB, we excluded SNPs with a genotyping efficiency below 80%, minor allele frequencies (MAF) below 1%, or Hardy-Weinberg equilibrium (HWE) values below 1×10^−8^. We performed pruning to eliminate genomic regions showing high linkage disequilibrium (LD) using a sliding window method with a size of 200 base pairs and a step of 50, retaining only SNPs with LD pairs (r2) below 0.2. We excluded samples with extreme heterozygosity, as well as individuals identified by UK Biobank as having high relatedness (UKB field P22027_0_0). Using PLINK, we also excluded samples showing unusual heterozygosity patterns on sex chromosomes. To control for relatedness, individuals related to the fourth degree were removed, following <https://choishingwan.gitlab.io/ukb-administration/admin/master_generation/#all-in-one-pipeline>. Our sample-level quality control also included the removal of participants who withdrew their consent (as of September 1, 2023). All quality control procedures were performed separately in each genetically inferred ancestry group, as defined by the Pan-UK Biobank^1^ .

# Functional mapping and pathway analysis

In FUMA, independent significant SNPs are identified by grouping all significant variants with LD r^2^≥0.6. A locus is defined by merging LD blocks of independent significant SNPs that are located within 250 kilobases of one another. The boundaries of the locus are determined by locating SNPs in LD (r^2^≥0.6) with the independent significant SNPs and establishing a region that includes all such SNPs within the locus. Additionally, lead SNPs within the locus are identified through successive clumping of all independent significant SNPs in the genomic region using an LD threshold of r^2^≥0.1. To check for enrichment in gene ontology and gene expression data from tissues in GTEx v8^2^ we used MAGMA^3^ v1.08. The gene expression dataset provided by FUMA was utilized to conduct tissue enrichment analysis. Pathway analyses of genes mapped by FUMA's SNP2GENE tool were carried out via FUMA GENE2FUNC with default parameters.

# Polygenic Risk score analysis in the UKB

Polygenic Risk Score for the CII was constructed by using PRS-CS^2^ (<https://github.com/getian107/PRScsx>) which estimates posterior SNP effect sizes under continuous shrinkage (CS) priors using GWAS summary statistics and an external linkage disequilibrium (LD) reference panel. Further, CII-PRS was normalized (z-scored).

# Marginal and conditional two sample Mendelian Randomization with mtCOJO

To check causality of associations between CII and sleep traits and diseases which showed significant genetic correlation with CII we used Genome-wide Complex Trait Analysis (GCTA) software tool^6^, where generalized summary-data-based Mendelian randomization (GSMR) and multi-trait conditional and joint analysis (mtCOJO) are performed.

For phenotypes which showed significant genetic correlation with CII we performed the following analyses

i. check causality of CII--phenotype associations using bi-directional GSMR between CII GWAS and phenotype GWAS
ii. check causality of CII--phenotype associations *conditional on CII components*, by performing GSMR between CII-conditional-on-component and phenotype-conditional-on-component GWAS summary statistics, obtained from GCTA mtCOJO analyses and then applying the bi-directional GSMR.

# PheWAS of the CII-PRS in the MGB biobank

The Mass General Brigham (MGB) Biobank is a clinical repository containing disease phenotypes and supplemented with genetic data collected from the MGB healthcare network in Massachusetts. Since its establishment in 2009, participant recruitment has occurred both online and in person across MGB-affiliated community-based primary care practices and tertiary care centers. A subset of participants (n = 64,639) contributed blood samples for genotyping. DNA was extracted and genotyped using the Infinium Global Screening Array-24 v2.0 (Illumina). Genotype imputation was performed via the Michigan Imputation Server using the Trans-Omics for Precision Medicine (TOPMed, version r2) reference panel, with haplotype phasing conducted using Eagle v2.3. Standard quality control procedures were applied to exclude low-quality variants and samples. Related individuals (kinship coefficient > 0.0625) were identified, and one sample from each pair was removed. To account for population structure, principal components of ancestry were derived using TRACE with the Human Genome Diversity Project reference panel. For the present analyses, only individuals of European ancestry were retained.

Case ascertainment was based on clinical phenotypes identified from ICD-9/-10 billing codes and mapped to PheWAS codes based on clinical similarity generated by the PheWAS R package^4^. A total of 1,567 disorders were considered in the analysis. Associations between the CII-PRS and each disorder were tested via the Wald test using logistic regressions adjusted for age, sex, genotyping array, batch, and PCs of ancestry. Significance was determined using Bonferroni-adjusted P values for the total number of tests (0.05/1,567=3.01750150E-5).

# PheWAS of the CII-PRS in the All of US

The All of Us Research Program, which emphasizes inclusion of historically underrepresented populations and individuals with diverse ancestry and life circumstances, represents one of the most ancestrally diverse biomedical research resources available[69]. The dataset includes survey responses, detailed blood measurements, accelerometer recordings, and genetic data. For this study, we used a subset of whole-genome sequencing data from version 8 of the release, which includes over 400,000 participants^5^. Data were accessed via the All of Us Researcher Workbench. Electronic health record (EHR) data are stored in the OMOP Common Data Model (CDM) version 5.4, which follows a standardized structure of predefined EHR tables. Further details on the PheWAS pipeline are described in Wielscher et al^6^

# Replication analysis in the Nurses’ Health Study II

## Phenotypes’ assessment

To replicate our GWAS findings we used a women-only, independent study cohort – the Nurses’ Health Study II (NHS2) – which contains relevant phenotype data to construct the CII. Chronotype was assessed in the NHS2 in the 2009 questionnaire, when NHS2 women were 45-62 years old, by asking the following question: “*One hears about morning and evening types of people. Which ONE of these types do you consider yourself to be?”*

with possible answers: *Definitely a morning type/ More of a morning than an evening type/ Neither/ More of an evening than a morning type/ Definitely an evening type.* Similarly, as in the UKB, we excluded women who did not provide an answer and dichotomized it to assess eveningness, by assigning 1 risk point if answered *More of an evening than a morning type* or *definitely an evening type* and 0 otherwise. Sleep duration was assessed in years 2001, 2009 and 2017 asking about average hours of sleep (“Average hours of sleep over a 24-hour period” with possible answers: <5; 5; 6; 7; 8; 9; 10+) and in 2009 additionally asking about it in specific age ranges (20-25, 26-35, 36-45, 46+). These assessments were used to estimate an average sleep duration for each individual, averaging in case of several assessments per individual. Further, the average sleep duration was dichotomized to “short or long sleep duration” by assigning 1 if <7h or >=9h and 0 otherwise. Caffeine consumption in the NHS2 was assessed multiple times through food frequency questionnaires (FFQs; assessed in 1991, 1995, 1999, 2003, 2007, 2011, 2015 and 2019). We averaged available assessments per individual and further dichotomized assigning 1 if consumed no or >400mg of caffeine per day and 0 otherwise. For the Vitamin D levels in the NHS2 we used predicted Vitamin D score.

Neuroticism score was not explicitly assessed in the NHS2; therefore, we derived it using available NHS2 questions (see *Supplement_4_ NHS2_CII_replication.docx*). We evaluated the psychometric properties of the 12-item neuroticism proxy in NHS2 (N=65,339). Internal consistency was estimated with Cronbach’s alpha using listwise complete cases, and corrected item–total correlations were computed as the correlation between each item and the total score minus that item. Because items were dichotomous (0/1), we estimated a **tetrachoric** correlation matrix and conducted a one-component principal components analysis to evaluate unidimensionality. A dominant factor was predefined as ≥40% variance explained with standardized loadings ≥0.40. Analyses were conducted in SAS 9.4. Results indicated a clear general factor (44.6% variance; all loadings ≥0.53), supporting use of a single neuroticism composite. Internal consistency was acceptable (α=0.78) with modest heterogeneity in item discriminations. In covariate-adjusted linear models, adding the neuroticism PRS increased explained variance by ΔR² = 0.025. The partial R² was 0.025 (95% CI 0.020–0.031), implying a partial correlation of about 0.16. This is within the expected 1–3% range for complex behavioral traits and supports the genetic validity of the NHS2 neuroticism proxy for replication analyses. Furthermore, LD score regression in the NHS2 estimated an observed-scale SNP-heritability of h²=0.0224 (SE=0.007), significantly above zero, with λGC=0.993 and mean χ²=0.997, indicating no genomic inflation; the intercept=0.9683 (0.0063) suggests slightly conservative test statistics (ratio not computed because mean χ²<1). For the UKB neuroticism, the corresponding estimates were h²=0.0886 (0.0029) with λGC=1.6032 and intercept=1.0313 (0.0101); the ratio=0.0381 (0.0123) implies that most of the inflation reflects true polygenicity rather than confounding. The cross-cohort genetic correlation was r_g_=0.718 (SE=0.137; 95% CI≈0.45–0.99; P=1.70×10⁻⁷), indicating substantial shared genetic architecture between the NHS2 proxy and UKB neuroticism. The lower h² in NHS2 is expected given smaller effective sample size and coarser measurement, but the high r_g_ supports using the NHS2 neuroticism proxy for replication analyses.

Obtained estimate measure of neuroticism score was then dichotomized by assigning 1 if >=7 and 0 otherwise.

Overall, the CII constructed in the NHS2 based on the five components constructed as described above showed a distribution very similar to the UKB-women derived CII (*Supplement_4_ NHS2_CII_replication.docx*). Phenotypic data for all the five CII components were available for 50,900 European ancestry NHS2 women. Of them 11,344 had also genetic data available. Socio economic status in the NHS2 was evaluated as in DeVille et.al. using a composite neighbourhood SES (nSES) index derived from nine census tract–level variables capturing education, employment, housing, wealth, racial/ethnic, and population composition, with higher values indicating higher neighbourhood SES.

## Replication of phenotypic associations with CKM

In our recent study by Zhang et.al^7^, CII was associated with increased cardio-kidney-metabolic (CKM) risk. Particularly, among individuals of European ancestry, high CII posed the greatest risk if combined with night shift work. Given that the methods for assessing individual CII components differ between NHS2 and UKB, we conducted a series of phenotypic association analyses to determine how well the CII constructed in NHS2 replicates the features represented by the UKB's CII. Particularly, we constructed an estimate for a cardio-kidney-metabolic syndrome (CKM) in the NHS2 by integrating a presence of diabetes mellitus (instead of type 2 diabetes), kidney stones (as a proxy for chronic kidney disease), myocardial infarction or a presence of coronary bypass (as a replacement for an information on cardiovascular diseases) in 2009. This approach was largely determined by the availability of specific assessments in the NHS2 2009 questionnaire, and disease presence was self-reported. Both of these factors could introduce bias when compared to ICD9/10 code-based assessments of CKM in the UKB and we acknowledge this as a limitation of our study. To avoid confusion with an actual CKM diagnosis derived from ICD-9/10 codes in the UKB, we refer to the outcome constructed in the NHS2 as "estimated CKM" (eCKM). Furthermore, following our analyses in Zhang et al., we also investigated whether the cumulative duration of shift work (until 2009) modified the association between CII and the eCKM.

The analysis of association between the CII and the estimated CKM in the NHS2 indicated increasing eCKM risk with higher CII levels and significant increasing trend (p_trend_<.0001; see 8_Supplement_NHS2_CII_Phenotypic_associations_andSW.docx; Table2). Furthermore, similarly as in the UKB, the risk of the eCKM increased with the higher CII level and duration of shift work, with significant trends in each of the three CII levels (Low (0-1), middle (2-3), high (4-5); 8_Supplement_NHS2_CII_Phenotypic_associations_andSW.docx; Tables 3-4). Interestingly, in the NHS2 this trend was observed already when considering cumulative duration of shift work categorized into never, less than 5 years, 5 or more years of shift work, whereas in the UKB it was only observed under categorization :never, less than 20 years of shift work, 20 or more years of shift work. This can be attributed to considerably larger sample of nurses performing shift work and good quality assessment of this phenotype in the NHS2. In summary, these phenotypic replication analyses confirm that the CII derived in the NHS2 shows characteristics and associations patterns similar to the ones observed in the UKB, which in our understanding qualifies it as a good quality phenotype to perform the replication of our GWAS.

## Replication of the CII GWAS results in the NHS2

Women with available CII assessment in the NHS2 were genotyped on four genotyping platforms: GlobalScreeningArray, HumanCoreExome2, Illumina HumanHap, OmniExpress, and OncoArray. Genotype imputation was performed using the Haplotype Reference Consortium (HRC; v.r1.1.2016) reference panel, and variants with imputation quality (R² < 0.7) or minor allele frequency (MAF < 0.01) were excluded. Additionally, individuals with a kinship coefficient > 0.1 were excluded. Replication analysis was performed with PLINK 1.9 using linear regression model adjusted for age at sample collection and 10 first principal components of ancestry.

# References

1.	ReproGen Consortium *et al.* An atlas of genetic correlations across human diseases and traits. *Nat. Genet.* **47**, 1236–1241 (2015).

2.	Watanabe, K., Taskesen, E., Van Bochoven, A. & Posthuma, D. Functional mapping and annotation of genetic associations with FUMA. *Nat. Commun.* **8**, 1826 (2017).

3.	Yang, J., Lee, S. H., Goddard, M. E. & Visscher, P. M. GCTA: A Tool for Genome-wide Complex Trait Analysis. *Am. J. Hum. Genet.* **88**, 76–82 (2011).

4.	Carroll, R. J., Bastarache, L. & Denny, J. C. R PheWAS: data analysis and plotting tools for phenome-wide association studies in the R environment. *Bioinformatics* **30**, 2375–2376 (2014).

5.	The All of Us Research Program Investigators. The “All of Us” Research Program. *N. Engl. J. Med.* **381**, 668–676 (2019).

6.	Wielscher, M. *et al.* Modeling Sleep Genetics Uncovers Distinct Disease Risks in Night vs. Day Workers. Preprint at https://doi.org/10.21203/rs.3.rs-7528705/v1 (2025).

7.	Zhang, J. A newly developed circadian imbalance index (CII) and risk of cardiovascular-kidney-metabolic disease in the UK biobank. https://doi.org/10.21203/rs.3.rs-7722286/v1 doi:https://doi.org/10.21203/rs.3.rs-7722286/v1.
