# Supplementary material for: Genetic architecture of a Circadian Imbalance Index: genome-wide association, phenome-wide association, and Mendelian randomisation analyses
Source: eBioMedicine. 2026 Jul 21;130:106380. doi: 10.1016/j.ebiom.2026.106380 (PMC13476902; doi:10.1016/j.ebiom.2026.106380)
Supplement: Supplementary file 3 [file mmc3.docx]

**Supplement 4: Replication of the CII in the NHS2 cohort**

Contents

[Questions used for derivation of the neuroticism score in the NHS2	1](#_Toc215596823)

[Neuroticism distribution in the UKB EUR women	2](#_Toc215596824)

[Neuroticism distribution in the NHS2 :	2](#_Toc215596825)

[Questions used in the UKB to assess neuroticism (QU1-QU12) and their suggested equivalents in the NHS2 (QN1-QN32)	2](#_Toc215596826)

[**Table N1:** NHS2 questions used as an equivalent to each of 12 neuroticism score questions used to assess neuroticism in the UKB.	1](#_Toc215596827)

[CII among UKB women and in the NHS2 cohort	1](#_Toc215596828)

[**Table N2:** Baseline characteristics of **N=164,462** **genotyped, European ancestry women from the UK Biobank** with available CII assessments, by CII value and CII components.	1](#_Toc215596829)

[**Table N3:** Characteristics of N=11,351 **genotyped, European ancestry women from the NHS2** with available CII assessments, by CII value and CII components.	3](#_Toc215596830)

[Age adjusted odds ratios for estimated cardiovascular-kidney-metabolic (eCKM) risk in the Nurses’ Health Study II	5](#_Toc215596831)

[**Table N4:** Age adjusted odds ratios (ORs) with 95% CI for estimated cardiovascular kidney-metabolic (eCKM) risk among N= 50,900 European ancestry female nurses from the Nurses’ Health Study II (NHS2) stratified by Circadian Imbalance Index (CII)	5](#_Toc215596832)

[**Table N5.** Age adjusted odds ratios (ORs) with 95% CI for estimated cardiovascular-kidney-metabolic (eCKM) risk among N= 50,900 European ancestry female nurses from the Nurses’ Health Study II (NHS2) stratified by Circadian Imbalance Index (CII) levels and a cumulative duration of shift work (never, less than 20 years or 20+years of shift work).	5](#_Toc215596833)

[**Table N6.** Age adjusted^*^ odds ratios (ORs) with 95% CI for estimated cardiovascular-kidney-metabolic (eCKM) risk among N= 50,900 European ancestry female nurses from the Nurses’ Health Study II (NHS2) stratified by Circadian Imbalance Index (CII) levels and a cumulative duration of shift work (never, less than 5 years or 5+ years of shift work).	6](#_Toc215596834)

[**Table N7.** Age adjusted^*^ odds ratios (ORs) with 95% CI for estimated cardiovascular-kidney-metabolic (eCKM) risk among N=11,344 European ancestry female nurses from the Nurses’ Health Study II (NHS2) stratified by cumulative duration of shift work (never, less than 5 years or 5+ years of shift work).	6](#_Toc215596835)

[Distributions of the CII and its components in the UKB women and NHS2	7](#_Toc215596836)

[Figure N1. Correlation between the CII and its components in the NHS2	8](#_Toc215596837)

[References	9](#_Toc215596838)

# Questions used for derivation of the neuroticism score in the NHS2

In the UKB neuroticism was defined as described in Smith et.al ^1^, based on **12 question**s, categorized in **three neuroticism clusters: worry, depressed affect and sensitivity to environmental stress and adversity (SESA).** To construct neuroticism in the NHS2 we collected questions from NHS2 questionnaires capturing the same or closely related aspects of personality (Table N1) and combined responses to them as a replacement for each UKB question. Responses to each question were dichotomized (Table N1) and - in case of repeated assessments for a participant over years and in case of several NHS2 questions corresponding to one UKB question- NHS2 **responses were combined into one using ‘OR’ logic. (1=YES, 0=NO).**

## Neuroticism distribution in the UKB EUR women

## Neuroticism distribution in the NHS2 :

## Questions used in the UKB to assess neuroticism (QU1-QU12) and their suggested equivalents in the NHS2 (QN1-QN32)

**Worry (QU1-QU4)**

**QU1: Are you a worrier?** [**Data-Field 1980**](https://biobank.ndph.ox.ac.uk/showcase/field.cgi?tk=nh7uzUNRLh8ELJ5aUOXMLPUYlKIq6Omp494278&id=1980)

**Distribution among UKB EUR women**

**Assessment in the NHS2 (QN1 -QN4):**

QN1:Over the last 4 weeks, how often have you been bothered by any of the following problems? Not being able to stop or control worrying

QN2: Feeling afraid as if something awful might happen

QN3 Do you find yourself worrying about getting some incurable illness?

QN4 Do you worry unduly when relatives are late coming home?

**Distribution among NHS2 women**

**QU2: Do you suffer from 'nerves'?** [**Data Field 2010**](https://biobank.ndph.ox.ac.uk/showcase/field.cgi?tk=nh7uzUNRLh8ELJ5aUOXMLPUYlKIq6Omp494278&id=2010)

**Distribution among UKB EUR women**

**Assessment in the NHS2: (QN5-QN6)**

QN5:Over the last 4 weeks, how often have you been bothered by any of the following problems? Feeling nervous, anxious or on edge

QN6 During the past 4 weeks, have you had any of the following problems with your work or other regular daily activities **as a result of any emotional problems (such as feeling depressed or anxious**)? Cut down the amount of time you spent on work or other activities

**Distribution among NHS2 women**

**QU3: Would you call yourself a nervous person?** [**Data field 1970**](https://biobank.ndph.ox.ac.uk/showcase/field.cgi?tk=nh7uzUNRLh8ELJ5aUOXMLPUYlKIq6Omp494278&id=1970)

**Distribution among UKB EUR women**

**Assessment in the NHS2: QN7**

QN7:How much of the time during the past 4 weeks: Have you been a very nervous person?

**Distribution among NHS2 women**

**QU4: Would you call yourself tense or 'highly strung'?** [**Data field 1990**](https://biobank.ndph.ox.ac.uk/showcase/field.cgi?tk=nh7uzUNRLh8ELJ5aUOXMLPUYlKIq6Omp494278&id=1990)

**Distribution among UKB EUR women**

**Assessment in the NHS2: (QN8-QN9)**

QN8: Over the last 4 weeks, how often have you been bothered by any of the following problems?

a) Trouble relaxing
b) Being so restless that it is hard to sit still

QN9: How much of the time during the past 4 weeks :Have you felt calm and peaceful?

**Distribution among NHS2 women**

**Depressed affect (QU5-QU8)**

**QU5: Do you often feel lonely?** [**Data field 2020**](https://biobank.ndph.ox.ac.uk/showcase/field.cgi?tk=nh7uzUNRLh8ELJ5aUOXMLPUYlKIq6Omp494278&id=2020)

**Distribution among UKB EUR women**

**Assessment in the NHS2: QN10**

QN10:during the past month : I felt lonely

**Distribution among NHS2 women**

**QU6: Do you ever feel 'just miserable' for no reason?** [**Data field 1930**](https://biobank.ndph.ox.ac.uk/showcase/field.cgi?tk=nh7uzUNRLh8ELJ5aUOXMLPUYlKIq6Omp494278&id=1930)

**Distribution among UKB EUR women**

**Assessment in the NHS2: (QN11-QN15)**

QN11:How much of the time during the past 4 weeks

a) Have you felt so down in the dumps nothing could cheer you up?
b) Have you felt downhearted and blue?
c) Did you feel worn out?

QN12:during the past month.

a) I felt depressed.
b) I felt that everything I did was an effort.
c) I could not “get going”.
d) I felt fearful.

QN13:Over the last 2 weeks, how often have you been bothered by the following problems? Feeling down, depressed, or hopeless

QN14 In your lifetime, have you ever had two weeks or longer when nearly every day you felt sad, blue, or depressed for most of the day?

QN15 In the past two years, has there been a time lasting 2 weeks or longer: When most of the day you felt sad, empty or depressed?

**Distribution among NHS2 women**

**QU7: Does your mood often go up and down?** [**Data field 1920**](https://biobank.ndph.ox.ac.uk/showcase/field.cgi?tk=nh7uzUNRLh8ELJ5aUOXMLPUYlKIq6Omp494278&id=1920)

**Distribution among UKB EUR women**

**Assessment in the NHS2: QN16**

QN16: Please indicate how often you have felt this way. During the past month I was bothered by things that usually don’t bother me.

**Distribution among NHS2 women**

**QU8: Do you often feel 'fed-up'?** [**Data field 1960**](https://biobank.ndph.ox.ac.uk/showcase/field.cgi?tk=nh7uzUNRLh8ELJ5aUOXMLPUYlKIq6Omp494278&id=1960)

**Distribution among UKB EUR women**

**Assessment in the NHS2: (QN17-QN19)**

QN17 How much of the time during the past 4 weeks...

a) Have you felt no interest in things?
b) Did you feel tired?

QN18 In the past two years, has there been a time lasting 2 weeks or longer:

a) When most of the day you were very discouraged about how things were going in your life?
b) When you lost interest in most things you usually enjoy like work, hobbies, and personal relationships?

QN19 There are many ways to try to deal with problems. These items ask what you do, in general, to cope with the stress in your life.

a) I give up trying to deal with things.
b) I give up the attempt to cope.

**Distribution among NHS2 women**

**SESA (sensitivity to environmental stress and adversity; QU9-QU12)**

**QU9: Are your feelings easily hurt?** [**Data field 1950**](https://biobank.ndph.ox.ac.uk/showcase/field.cgi?tk=nh7uzUNRLh8ELJ5aUOXMLPUYlKIq6Omp494278&id=1950)

**Distribution among UKB EUR women**

**Assessment in the NHS2: (QN20-QN21)**

QN20: In the past two years, has there been a time lasting 2 weeks or longer: When most days you felt much more anxious and worried than other people with the same problems as you?

QN21 Please indicate the extent to which you agree or disagree with the following statements.

a) If something can go wrong with me, it will.
b) I hardly ever expect things to go my way.
c) I rarely count on good things happening to me.

**Distribution among NHS2 women**

**QU10: Do you worry too long after an embarrassing experience?** [**Data field 2000**](https://biobank.ndph.ox.ac.uk/showcase/field.cgi?tk=nh7uzUNRLh8ELJ5aUOXMLPUYlKIq6Omp494278&id=2000)

**Distribution among UKB EUR women**

**Assessment in the NHS2: QN22**

QN22:Over the last 4 weeks, how often have you been bothered by any of the following problems? Worrying too much about different things

**Distribution among NHS2 women**

**QU11: Are you often troubled by feelings of guilt?** [**Data field 2030**](https://biobank.ndph.ox.ac.uk/showcase/field.cgi?tk=nh7uzUNRLh8ELJ5aUOXMLPUYlKIq6Omp494278&id=2030)

**Distribution among UKB EUR women**

**Assessment in the NHS2: (QN23-QN24)**

QN23:What you do, in general, to cope with the stress in your life.

a) I blame myself for things that happened.
b) I criticize myself.

QN24 In your life, have you ever had any experience that was so frightening, horrible, or upsetting that, in the past month, you: Felt guilty or unable to stop blaming yourself or others for the event

**Distribution among NHS2 women**

**QU12: Are you an irritable person?** [**Data field 1940**](https://biobank.ndph.ox.ac.uk/showcase/field.cgi?tk=nh7uzUNRLh8ELJ5aUOXMLPUYlKIq6Omp494278&id=1940)

**Distribution among UKB EUR women**

**Assessment in the NHS2: (QN25-QN26)**

QN25:How much of the time during the past 4 weeks: Did you feel full of pep?

QN32: Over the last two weeks, how often have you been bothered by the following problems? Becoming easily annoyed or irritable

**Distribution among NHS2 women**

### **Table N1:** NHS2 questions used as an equivalent to each of 12 neuroticism score questions used to assess neuroticism in the UKB.

| **Neuroticism cluster** | **UKBB question** | **Possible answers** | **NHS2 questions** | **Possible answers/ Coding (0=No; 1=Yes)** | **NHS2 question’s assessment years** |
| --- | --- | --- | --- | --- | --- |
| **Worry** | QU1:Are you a worrier? | Yes  No  Do not know  Prefer not to answer | QN1:Over the last 4 weeks, how often have you been bothered by any of the following problems?  Not being able to stop or **control worrying**  QN2: Feeling afraid as if something awful might happen  QN3 Do you find yourself **worrying about getting some incurable illness?**  QN4 Do you **worry unduly** when relatives are late coming home? | Nearly every day=1  More than half the days=1  Several days=1  Not at all=0  not at all=0  several days=1  >half the days=1  nearly every day=1  often=1  sometimes=0  never=0  Yes=1  No=0 | 2013, 2017  2013, 2017  1993,2005  1993,2005 |
|  | QU2:Do you suffer from **'nerves'?** | Yes  No  Do not know  Prefer not to answer | QN5:Over the last **4 weeks**, how often have you been bothered by any of the following problems?  Feeling **nervous, anxious or on edge**  QN6 During the past 4 weeks, have you had any of the following problems with your work or other regular daily activities **as a result of any emotional problems (such as feeling depressed or anxious**)?   a) Cut down the amount of time you spent on work or other activities | Nearly every day=1  More than half the days=1  Several days=0  Not at all=0  Yes=1  No=0 | 2013, 2017  1993, 1997, 2001 |
|  | QU3:Would you call yourself a **nervous person?** | Yes  No  Do not know  Prefer not to answer | QN7:How much of the time **during the past 4 weeks**  - Have you been a **very nervous person**? | All of the time=1  Most of the time=1  A Good Bit of the time =1  Some of the time=0  A tittle of the time=0  None of the time=0 | 1993, 1997, 2001 |
|  | QU4:Would you call yourself **tense or 'highly strung'**? | Yes  No  Do not know  Prefer not to answer | QN8:Over the last 4 weeks, how often have you been bothered by any of the following problems?   a) **Trouble relaxing** b) Being so **restless t**hat it is hard to sit still   QN9:How much of the time during the past 4 weeks  - Have you **felt calm and peaceful?** | Nearly every day=1  More than half the days=1  Several days=0  Not at all=0  All of the time=0  Most of the time=0  A Good Bit of the time=0  Some of the time=0  A tittle of the time=0  None of the time=1 | 2013, 2017  1993,1997, 2001 |
| **Depressed affect** | QU5:Do you often **feel lonely?** | Yes  No  Do not know  Prefer not to answer | QN10:during the past month.  **I felt lonely** | All of the time=1  Occasionally or a moderate amount of time=1  Some or a little of the time=0  Rarely or none of the time=0 | 2013,2017 |
|  | QU6:Do you ever **feel 'just miserable'** for no reason? | Yes  No  Do not know  Prefer not to answer | QN11:How much of the time during the **past 4 weeks**  - Have you felt so **down in the dumps** nothing could cheer you up?  - Have you felt **downhearted and blue**?  - Did you feel **worn out**?  QN12:during the past month.  a:I felt **depressed**.  b:I felt that everything I did was an effort.  c:I **could not “get going”.**  d)I felt fearful.  **QN13** In your lifetime, have you ever had two weeks or longer when nearly every day you felt **sad,**  **blue, or depressed for most of the day**?  QN14 In the past two years, has there been a time lasting 2 weeks or longer:  When most of the day you felt **sad, empty or depressed?** | All of the time=1  Most of the time=1  A Good Bit of the time =1  Some of the time =0  A tittle of the time=0  None of the time=0  All of the time=1  Occasionally or a moderate amount of time=1  Some or a little of the time=0  Rarely or none of the time=0  Yes=1  No=0  Yes=1  No=0 | 1993, 1997, 2001  2013,2017  2001,2003,05,07,09,2011,2015,  2013 |
|  | QU7:Does your mood often go up and down?  Refers to emotional stability | Yes  No  Do not know  Prefer not to answer | QN15: During tho past 4 weeks, how much of the time has your physical health or **emotional problems interfered with your social activities** (like visiting with friends, relatives, etc.)?  QN16 During the past 4 weeks, to what extent has your physical health **or emotional problems interfered with your normal social activities** with family, friends, neighbors, or groups?  QN17 Please indicate how often you have felt this way.  During the past month I was bothered by things that usually don’t bother me. | All of the time =1  Most of the time =1  Some of the time =1  A little of the time=0  None of the time=0  Not at all =0  Slightly =0  Moderately =0  Quite a bit =1  Extremely =1  Rarely=0  little of time=1  moderate amount=1  all of the time=1 | 1993, 1997, 2001  1993, 1997, 2001  2013,2017 |
|  | QU8:Do you often feel **'fed-up'**? | Yes  No  Do not know  Prefer not to answer | QN18 How much of the time during the past 4 weeks...  Have you felt no interest in things?  Did you feel tired?  QN19 In the past two years, has there been a time lasting 2 weeks or longer:  b. When most of the day you were  very discouraged about how things  were going in your life?  c. When you lost interest in most things  you usually enjoy like work, hobbies,  and personal relationships?  QN20 There are many ways to try to deal with problems. These items ask what  you do, in general, to **cope with the stress** in your life.  I give up trying to deal with things.  I give up the attempt to cope. | All of the time=1  Most of the time=1  A Good Bit of the time=1  Some of the time =1  A tittle of the time=1  None of the time=0  All of the time =1  Most of the time=1  A Good Bit of the time=1  Some of the time=1  A tittle of the time=0  None of the time=0  Yes=1  No=0  not at all=0  a little bit=1  a medium amount of time=1  a lot=1  not at all=0  a little bit=0  a medium amount of time=1  a lot=1 | 2001  1993, 1997, 2001  2013  2001  2001 |
| **SESA (sensitivity to environmental stress and adversity)** | QU9:Are your feelings easily hurt? | Yes  No  Do not know  Prefer not to answer | QN21: In the **past two years**, has there been a time lasting **2 weeks or longer**:  When most days you felt much **more anxious and worried** than other people with the same problems as you?  QN22 Please indicate the extent to which you agree or disagree with the following statements..   a) If something can go wrong with me, it will. b) I hardly ever expect things to go my way. c) I rarely count on good things happening to me. | Yes=1  No=0  Disagree strongly=0  Neither agree nor disagree=0  Disagree a little=1  Agree a little=1  Agree strongly=1 | 2013  2017 |
|  | QU10:Do you **worry too long** after an embarrassing experience? | Yes  No  Do not know  Prefer not to answer | QN23:Over the last 4 weeks, how often have you been bothered by any of the following problems?  **Worrying too much** about different things | Nearly every day=1  More than half the days=1  Several days=1  Not at all=0 | 2013, 2017 |
|  | QU11:Are you often troubled by feelings of guilt? | Yes  No  Do not know  Prefer not to answer | QN24:What you do, in general, to cope with the stress in your life.   a) I **blame myself for things that happened**. b) I criticize myself.   QN25 In your life, have you ever had any experience that was so frightening, horrible, or upsetting that, in the past month, you:  **Felt guilty or unable to stop blaming yourself** or others for the event? | Not at all=0  A little Bit=0  A medium Amount=1  A lot=1  Yes =1  No=0 | 2001  2017 |
|  | QU12:Are you an **irritable person**? | Yes  No  Do not know  Prefer not to answer | QN26:How much of the time **during the past 4 weeks**  - Did you feel **full of pep**?  QN27: Over the **last two weeks**, how often have you been bothered by the following problems?  Becoming easily **annoyed or irritable** | All of the time=1  Most of the time=0  A Good Bit of the time=0  Some of the time =0  A tittle of the time=0  None of the time=0  Not at all=0  Several days=1  More than half the days=1  Nearly every day=1 | 1993, 1997, 2001  2013, 2017 |

# CII among UKB women and in the NHS2 cohort

### **Table N2:** Baseline characteristics of **N=164,462** **genotyped, European ancestry women from the UK Biobank** with available CII assessments, by CII value and CII components.

|  | | **CII value** | | | | | |  |
| --- | --- | --- | --- | --- | --- | --- | --- | --- |
|  |  | **0** | **1** | **2** | **3** | **4** | **5** | **Total** |
| **N (%)** | | 13,728 (8.3) | 43,775 (26.6) | 55,061 (33.5) | 35,986 (21.9) | 13,473 (8.2) | 2,439 (1.5) | 164,462 (100.0) |
| **Age*** | | 57.46 (7.77) | 56.69 (7.93) | 56.23 (7.98) | 55.68 (8.03) | 55.20 (7.98) | 54.67 (7.99) | 56.2 (8.0) |
| **Average total household income before tax, N (%)** | **Less than** £**18,000** | 2,212 (16.1) | 7,375 (16.9) | 1,0347 (18.8) | 7,771 (21.6) | 3,534 (26.4) | 794 (32.8) | 32,033 (19.5) |
|  | £**18,000 to** £**31,000** | 3,000 (21.9) | 9,740 (22.3) | 12,095 (22.0) | 8,099 (22.6) | 2,980 (22.2) | 508 (21.0) | 36,422 (22.2) |
|  | £**31,000 to** £**52,000** | 3,116 (22.7) | 9,904 (22.7) | 12,407 (22.6) | 7,851 (21.9) | 2,765 (20.6) | 455 (18.8) | 36,498 (22.2) |
|  | £**52,000 to** £**100,000** | 2,537 (18.5) | 8,016 (18.3) | 9,669 (17.6) | 5,684 (15.8) | 1,764 (13.2) | 251 (10.4) | 27,921 (17.0) |
|  | **Greater than** £**100,000** | 831 ( 6.1) | 2,364 ( 5.4) | 2,499 ( 4.5) | 1,232 ( 3.4) | 386 ( 2.9) | 50 ( 2.1) | 7,362 ( 4.5) |
|  | **Unknown** | 2021 (14.7) | 6316 (14.4) | 7,944 (14.5) | 5,259 (14.7) | 1,981 (14.8) | 364 (15.0) | 23,885 (14.6) |
| **Education, N (%)** | **College or University degree** | 4,688 (34.1) | 14,664 (33.5) | 17,705 (32.2) | 10,495 (29.2) | 3,500 (26.0) | 554 (22.7) | 51,606 (31.4) |
|  | **Other** | 7,092 (51.7) | 22,541 (51.5) | 28,444 (51.7) | 18,991 (52.8) | 7,086 (52.6) | 1,323 (54.2) | 85,477 (52.0) |
|  | **Unknown** | 1,948 (14.2) | 6,570 (15.0) | 8,912 (16.2) | 6,500 (18.1) | 2,887 (21.4) | 562 (23.0) | 27379 (16.6) |
| **BMI*** | | 25.71 (4.13) | 26.29 (4.58) | 26.99 (5.10) | 27.63 (5.49) | 28.36 (5.91) | 29.37 (6.40) | 27.0 (5.1) |
| **Shift workers, N (%^(a)^)** | | 779 (11.1) | 2,929 (12.0) | 4,277 (13.4) | 3,206 (15.4) | 1,338 (17.8) | 276 (23.0) | 12,805 (7.8%) |
|  | | **CII components** | | | | | |  |
|  |  | **Evening Person** | **Short Or Long Sleep** | **High Neuroticism** | **No Or High Caffeine** | **Low Vit D** | **Total** |  |
| **N (%)** | | 55,730 (33.9) | 50,798 (30.9) | 45,764 (27.8) | 88,703 (53.9) | 86,947 (52.9) | 164,462 (100.0) |  |
| **Age*** | | 55.49 (8.16) | 57.06 (7.72) | 55.18 (7.94) | 55.85 (8.02) | 55.84 (8.03) | 56.22 (7.99) |  |
| **Average total household income before tax, N (%)** | **Less than £18,000** | 10,810 (19.4) | 12,320 (24.3) | 10,455 (22.9) | 17,921 (20.3) | 17,982 (20.7) | 32,033 (19.5) |  |
|  | **£18,000 to £31,000** | 12,287 (22.1) | 11,370 (22.5) | 10,160 (22.3) | 19,761 (22.3) | 19,109 (22.0) | 36,422 (22.2) |  |
|  | **£31,000 to £52,000** | 12,640 (22.7) | 10,174 (20.1) | 9,870 (21.6) | 19,521 (22.1) | 19,401 (22.4) | 36,498 (22.2) |  |
|  | **£52,000 to £100,000** | 9,781 (17.6) | 6,772 (13.4) | 6,925 (15.2) | 14,518 (16.4) | 14,721 (17.0) | 2,7921 (17.0) |  |
|  | **Greater than £100,000** | 2,564 ( 4.6) | 1,667 ( 3.3) | 1,460 ( 3.2) | 3,508 ( 4.0) | 3,653 ( 4.2) | 7,362 ( 4.5) |  |
|  | **Unknown** | 7,518 (13.5) | 8,332 (16.5) | 6,760 (14.8) | 13,262 (15.0) | 11,853 (13.7) | 23,885 (14.6) |  |
| **Education, N (%)** | **College or University degree** | 18,147 (32.6) | 13,152 (25.9) | 12,330 (26.9) | 25,622 (28.9) | 29,078 (33.4) | 51,606 (31.4) |  |
|  | **Other** | 29,377 (52.7) | 26,710 (52.6) | 24,353 (53.2) | 47,259 (53.3) | 43,662 (50.2) | 85,477 (52.0) |  |
|  | **Unknown** | 8,206 (14.7) | 10,936 (21.5) | 9,081 (19.8) | 15,822 (17.8) | 14,207 (16.3) | 27,379 (16.6) |  |
| **Shift workers, N (%^(a)^)** | | 4,933 (15.2) | 4,444 (17.2) | 3,881 (15.0) | 7,316 (14.4) | 7,259 (14.1) | 12,805 (13.8) |  |
| **BMI*** | | 27.29 (5.30) | 27.60 (5.47) | 27.16 (5.39) | 27.15 (5.24) | 27.79 (5.59) | 26.98 (5.13) |  |

N: sample size; BMI: Body mass index;

*Values are **means** ± **standard deviations;**

**^(a)^** Percentages among N=92,838 genotyped European ancestry female UKB participants with all CII components available, who replied to the baseline questionnaire (2006-2010) and answered to the question if their primary job involved shift work (defined as work schedules outside 9am-5pm). Shift workers are those who answered with ‘sometimes’, ‘usually’ or ‘always’. Participants who answered with ‘prefer not to answer’ and ‘do not know’ were excluded from this analysis

### **Table N3:** Characteristics of N=11,351 **genotyped, European ancestry women from the NHS2** with available CII assessments, by CII value and CII components.

|  | **CII value** | | | | | |  |
| --- | --- | --- | --- | --- | --- | --- | --- |
|  | **0** | **1** | **2** | **3** | **4** | **5** | **Total** |
| **N (%)** | 801(7.1) | 2,739(24.1) | 3,633(32.0) | 2,748(24.2) | 1,195(10.5) | 235(2.1) | 11,351 |
| **Age*** | 55.64(4.24) | 55.51(4.45) | 55.31(4.44) | 55.38(4.35) | 55.36(4.36) | 54.65(4.49) | 55.39(4.40) |
| **Socio-economic status*^(a)^** | 0.81(4.12) | 0.40 (3.98) | -0.13 (3.78) | -0.56 (3.64) | -0.81 (3.44) | -0.80 (3.30) | -0.12 (3.81) |
| **Shift workers, N (%^(b)^)** | 537 (67.04) | 1,900 (69.37) | 2,589 (71.30) | 2,021(73.54) | 889 (74.58) | 188 (80.69) | 8,124 (71.6) |
| **BMI^*^** | 24.09(3.25) | 25.23(4.68) | 27.12(5.96) | 28.86(6.77) | 30.40(6.92) | 33.12(7.36) | 27.34(6.22) |
|  | **CII components** | | | | | |  |
|  | **Evening Person** | **Short Or Long Sleep** | **High Neuroticism** | **No Or High Caffeine** | **Low Vit D** | **Total** |  |
| **N (%)** | 3,862 (34.0) | 4,425 (39.0) | 2,960 (26.1) | 7,281(64.1) | 5,676 (50.0) | 11,351 |  |
| **Age*** | 55.35(4.43) | 55.32(4.38) | 55.11(4.36) | 55.28(4.47) | 55.51(4.30) | 55.39(4.40) |  |
| **Socio-economic status*^(a)^** | -0.25 (3.80) | -0.29 (3.66) | -0.27 (3.74) | -0.27 (3.79) | -0.75 (3.44) | -0.12 (3.81) |  |
| **Shift workers, N (%^(b)^)** | 2,821 (34.72) | 3,323 (40.90) | 2,224 (27.38) | 5,155 (63.45) | 4,114 (50.64) | 8,124 (71.6) |  |
| **BMI*** | 28.28(6.59) | 27.79(6.57) | 28.05(6.65) | 27.54(6.38) | 30.39 (6.75) | 27.34(6.22) |  |

N: sample size; BMI: Body mass index;

*Values are **means** (**standard deviations);**

**^(a)^** Socio economic status in the NHS2 was evaluated as in DeVille et.al.^2^ using a composite neighborhood SES (nSES) index derived from nine census tract–level variables capturing education, employment, housing, wealth, racial/ethnic, and population composition. For each participant address, variables were obtained from the U.S. Census (1990, 2000, or 2010) corresponding to the address period, z-standardized, and summed to generate the nSES score, with higher values indicating higher neighborhood SES.

**^(b)^** Percentages among N=11,344 genotyped European ancestry NHS2 women with all CII components available, with shift work assessment. Shift workers in the NHS2 were defined as ever performing shift work (before assessment year 2009). Among 11,344 women with shift work assessments N=8,124 (71.6%) were shift workers and N=3,220 (28.4%) never performed shift work (before year 2009

# Age adjusted odds ratios for estimated cardiovascular-kidney-metabolic (eCKM) risk in the Nurses’ Health Study II

### **Table N4:** Age adjusted odds ratios (ORs) with 95% CI for estimated cardiovascular kidney-metabolic (eCKM) risk among N= 50,900 European ancestry female nurses from the Nurses’ Health Study II (NHS2) stratified by Circadian Imbalance Index (CII)

| **CII** | **N** | **Cases (%of N)** | **OR (95%CI)** | ***P*_trend_** |
| --- | --- | --- | --- | --- |
| 0-1 | 15,672 | 894 (5.7) | Reference | <.0001 |
| 2 | 16,407 | 1,529 (9.3) | 1.71 (1.57;1.87) |  |
| 3 | 12,421 | 1,625 (13.1) | 2.52 (2.31;2.74) |  |
| 4 | 5,368 | 895 (16.7) | 3.39 (3.07;3.74) |  |
| 5 | 1,032 | 216 (20.9) | 4.58 (3.88;5.40) |  |

### **Table N5.** Age adjusted odds ratios (ORs) with 95% CI for estimated cardiovascular-kidney-metabolic (eCKM) risk among N= 50,900 European ancestry female nurses from the Nurses’ Health Study II (NHS2) stratified by Circadian Imbalance Index (CII) levels and a cumulative duration of shift work (never, less than 20 years or 20+years of shift work).

| **Characteristic** | **N** | **Cases (%)** | **OR (95% CI)** | ***P_trend_*** |
| --- | --- | --- | --- | --- |
| **Low CII (0-1)** |  |  |  |  |
| Day workers | 4900 | 276 (5.6) | Reference | 0.3441 |
| Night shift work< 20 years | 10663 | 608 (5.7) | 1.05 (0.91;1.22) |  |
| Night shift work>= 20 years | 109 | 10 (9.2) | 1.56 (0.81;3.04) |  |
| **Middle CII (2-3)** |  |  |  |  |
| Day workers | 8279 | 858 (10.4) | 1.97 (1.71;2.26) | 0.0027 |
| Night shift work< 20 years | 20220 | 2246 (11.1) | 2.19 (1.93;2.49) |  |
| Night shift work>= 20 years | 329 | 50 (15.2) | 2.82 (2.03;3.90) |  |
| **High CII (4-5)** |  |  |  |  |
| Day workers | 1614 | 267 (16.5) | 3.44 (2.88;4.12) | 0.0423 |
| Night shift work< 20 years | 4660 | 808 (17.3) | 3.73 (3.23;4.31) |  |
| Night shift work>= 20 years | 126 | 36 (28.6) | 6.42 (4.28;9.64) |  |

^*^Model adjusted for age at CII and eCKM assessment (2009);

### **Table N6.** Age adjusted^*^ odds ratios (ORs) with 95% CI for estimated cardiovascular-kidney-metabolic (eCKM) risk among N= 50,900 European ancestry female nurses from the Nurses’ Health Study II (NHS2) stratified by Circadian Imbalance Index (CII) levels and a cumulative duration of shift work (never, less than 5 years or 5+ years of shift work).

| **Characteristic** | **N** | **Cases** | **OR (95% CI)** | ***P_trend_*** |
| --- | --- | --- | --- | --- |
| **Low CII (0-1)** |  |  |  |  |
| Day workers | 4900 | 276 (5.6) | Reference | 0.0086 |
| Night shift work< 5 years | 7825 | 410 (5.2) | 0.96 (0.82;1.12) |  |
| Night shift work>= 5 years | 2947 | 208 (7.1) | 1.34 (1.11;1.61) |  |
| **Middle CII (2-3)** |  |  |  |  |
| Day workers | 8279 | 858 (10.4) | 1.97 (1.71;2.26) | <.0001 |
| Night shift work< 5 years | 13806 | 1448 (10.5) | 2.05 (1.79;2.34) |  |
| Night shift work>= 5 years | 6743 | 848 (12.6) | 2.53 (2.20; 2.91) |  |
| **High CII (4-5)** |  |  |  |  |
| Day workers | 1614 | 267 (16.5) | 3.45 (2.88;4.12) | 0.013 |
| Night shift work< 5 years | 3023 | 500 (16.5) | 3.53 (3.02;4.12) |  |
| Night shift work>= 5 years | 1763 | 344 (19.5) | 4.29 (3.62;5.09) |  |

^*^Model adjusted for age at CII and eCKM assessment (2009);

### **Table N7.** Age adjusted^*^ odds ratios (ORs) with 95% CI for estimated cardiovascular-kidney-metabolic (eCKM) risk among N=11,344 European ancestry female nurses from the Nurses’ Health Study II (NHS2) stratified by cumulative duration of shift work (never, less than 5 years or 5+ years of shift work).

| **Characteristic** | **N** | **Cases** | **OR (95% CI)** | ***P_trend_*** |
| --- | --- | --- | --- | --- |
| **Low CII (0-1)** |  |  |  |  |
| Day workers | 4900 | 276 (5.6) | Reference | 0.0086 |
| Night shift work< 5 years | 7825 | 410 (5.2) | 0.96 (0.82;1.12) |  |
| Night shift work>= 5 years | 2947 | 208 (7.1) | 1.34 (1.11;1.61) |  |
| **Middle CII (2-3)** |  |  |  |  |
| Day workers | 8279 | 858 (10.4) | 1.97 (1.71;2.26) | <.0001 |
| Night shift work< 5 years | 13806 | 1448 (10.5) | 2.05 (1.79;2.34) |  |
| Night shift work>= 5 years | 6743 | 848 (12.6) | 2.53 (2.20; 2.91) |  |
| **High CII (4-5)** |  |  |  |  |
| Day workers | 1614 | 267 (16.5) | 3.45 (2.88;4.12) | 0.013 |
| Night shift work< 5 years | 3023 | 500 (16.5) | 3.53 (3.02;4.12) |  |
| Night shift work>= 5 years | 1763 | 344 (19.5) | 4.29 (3.62;5.09) |  |

^*^Model adjusted for age at CII and eCKM assessment (2009);

### Distributions of the CII and its components in the UKB women and NHS2

**CII in the UKB EUR women	CII in the NHS2 :**

### Figure N1. Correlation between the CII and its components in the NHS2

# References

1.	Smith, D. J. *et al.* Prevalence and Characteristics of Probable Major Depression and Bipolar Disorder within UK Biobank: Cross-Sectional Study of 172,751 Participants. *PLoS ONE* **8**, e75362 (2013).

2.	DeVille, N. V. *et al.* Neighborhood socioeconomic status and mortality in the nurses’ health study (NHS) and the nurses’ health study II (NHSII). *Environ. Epidemiol.* **7**, e235 (2023).
